# INsPECT, an Open-Source and Versatile Software for Automated Quantification of (*Leishmania*) Intracellular Parasites

**DOI:** 10.1371/journal.pntd.0002850

**Published:** 2014-05-15

**Authors:** Ehsan Yazdanparast, Antonio Dos Anjos, Deborah Garcia, Corinne Loeuillet, Hamid Reza Shahbazkia, Baptiste Vergnes

**Affiliations:** 1 Universidade do Algarve, DEEI-FCT, Faro, Portugal; 2 MIVEGEC (UM1-CNRS 5290-IRD 224), Institut de Recherche pour le Développement (IRD), Montpellier, France; 3 UMR 5163, Centre National de la Recherche Scientifique (CNRS), Université Grenoble 1, Grenoble, France; National Institutes of Health, United States of America

## Abstract

Intracellular protozoan parasites are causative agents of infectious diseases that constitute major health problems for developing countries. *Leishmania* sp., *Trypanosoma cruzi or Toxoplasma gondii* are all obligate intracellular protozoan parasites that reside and multiply within the host cells of mammals, including humans. Following up intracellular parasite proliferation is therefore an essential and a quotidian task for many laboratories working on primary screening of new natural and synthetic drugs, analyzing drug susceptibility or comparing virulence properties of natural and genetically modified strains. Nevertheless, laborious manual microscopic counting of intracellular parasites is still the most commonly used approach. Here, we present INsPECT (Intracellular ParasitE CounTer), an open-source and platform independent software dedicated to automate infection level measurement based on fluorescent DNA staining. It offers the possibility to choose between different types of analyses (fluorescent DNA acquisitions only or in combination with phase contrast image set to further separate intra- from extracellular parasites), and software running modes (automatic or custom). A proof-of-concept study with intracellular *Leishmania infantum* parasites stained with DAPI (4′,6-diamidino-2-phenylindole) confirms a good correspondence between digital results and the “gold standard” microscopic counting method with Giemsa. Interestingly, this software is versatile enough to accurately detect intracellular *T. gondii* parasites on images acquired with High Content Screening (HCS) systems. In conclusion, INsPECT software is proposed as a new fast and simple alternative to the classical intracellular *Leishmania* quantification methods and can be adapted for mid to large-scale drug screening against different intracellular parasites.

## Author Summary

Research on intracellular parasites require using non-invasive technologies to follow up parasite proliferation inside their natural host cells by staying in the more physiological conditions as possible. High Content Screening (HCS) technology has recently emerged as a powerful image-based approach to screen new anti-parasitic compounds or to test parasite susceptibility to existing drugs *in vitro*. Nevertheless, such equipments will remain poorly accessible for most of academic and clinical diagnostic laboratories that mostly use more affordable, but laborious, microscopic counting procedures. The current work proposes new image-based, open-source software which provides a fast and accurate solution for investigating intracellular parasite quantification. Through an easy-to-use interface, cells' and parasites' information are dug out from DNA fluorescent images, and host cells' boundaries are extracted from corresponding phase contrast image set. Parasites are then reassigned to their related cells and intra/extracellular parasites are discriminated for each cell. The software further automatically calculates all data required for most of experimental infection studies. INsPECT software is proposed as a free substitute or complement to the available quantification methods for measuring *Leishmania* infection level *in vitro*. It may be enlarged, however, to different intracellular trypanosomatids or unrelated parasites such as *T. gondii*.

## Introduction

Intracellular protozoan parasites are responsible for worldwide infectious diseases with a high impact in public health for developing countries. Among them, *Leishmania* parasites are causative agents of leishmaniasis, a worldwide endemic disease with diverse clinical manifestations ranging from self-healing skin ulcers to fatal outcome, depending on parasite species and host immune status or genetics [1]. In mammals, *Leishmania* parasites replicate as amastigotes within parasitophorous vacuoles (PVs) of macrophages. Without a vaccine and taking into account the limited number of existing drugs, the screening of new anti-leishmanial compounds or characterization of new drug target candidates is therefore a research priority. Drug resistance to available treatments has also been well documented in certain areas, and partially explained in natural or experimentally resistant laboratory strains [2], [3]. Parasites isolated from patients refractory to treatment confirm the presence of circulating resistant parasites [4] and imply a careful surveillance of parasite drug susceptibility. All these research activities require the use of common calibrated and reliable procedures to monitor parasite proliferation inside host cell, maintaining physiological conditions as much as possible [5]. The most popular method still consists of manually counting intracellular parasites after Giemsa staining [6]. This direct counting approach is largely employed because Giemsa stain is cheap and only requires basic equipment (i.e light microscope). Microscopic counting is, however, a laborious task merely providing a global estimation of the parasite burden that is further highly prone to operator experience and subjectivity. Numerous alternative indirect approaches have been proposed in *Leishmania* [7]–[11]. Most are based on reporter genes assays that increase screening capacities while limiting human intervention. Indirect reporter assays, however, average the biological response of thousands of cells without integrating critical factors such as the percentage of infection and the discrimination between intra- and extracellular parasites. Homogenous expression of reporter genes over time and biological stages in *Leishmania* further require genomic integration [7], [8]. These transformation and selection steps can have a profound impact on the phenotypic traits of the original parasite population. This is particularly relevant when testing susceptibility of parasite strains isolated from drug unresponding patients.

The development of image processing and analysis techniques (together with the improvement of fluorescence microscopy instrumentation) has recently emerged as a new powerful solution for drug screening and susceptibility tests against intracellular protozoan parasites. High Content Screening (HCS) systems have been indeed successfully adapted to major intracellular protozoan parasites and allow direct analysis of both parasite and host cell responses to drug candidates [12]–[15]. However, considering the cost of such equipment and the expertise required to manage HCS instrumentation and analysis, those approaches will remain restricted to few laboratories in the world.

Extracting and quantifying information from biological images is a well-defined and common task in image processing. Some open source solutions such as ImageJ software (http://rsb.info.nih.gov/ij) are very helpful for biologists with more than 500 plugins available. Nevertheless, mastering image processing concepts require specific competences and, to our knowledge, no versatile open-access solutions for biologists are available to automate intracellular parasite quantification.

We present INsPECT (INtracellular ParasitE CounTer), the first open-source Java based software that uses fluorescent DNA staining and image processing framework to automate infection level measurement. This can be done through a user-friendly interface, where image files obtained from any fluorescent microscope and magnification, can be processed individually or as a batch, automatically or in a custom mode, without any experience required on image analysis. The software runs either with DNA fluorescent image files alone, or in combination with the corresponding phase contrast or DIC (Differential Interference Contrast) image set. Providing this complementary information allows automatic cell boundaries detection and discrimination between intra- and extracellular parasites without any additional use of fluorescent cytoplasm/membrane marker. Output files comprise annotated images and a report table with all information needed for most of experimental infection studies *in vitro*: total number of cells, total number of parasites, percentage of infected cells, mean number of parasite/cell, parasitic index. Software robustness was validated for the calculation of Ec50 toward intramacrophagic *L. infantum* treated with pentavalent antimonials (glucantime) as a proof-of-concept study. Alternative analyses performed on *T. gondii* infected fibroblasts further confirm that INsPECT software utilization may be enlarged to unrelated intracellular parasites.

## Methods

### 1. Image processing and software development

**Figure 1:**
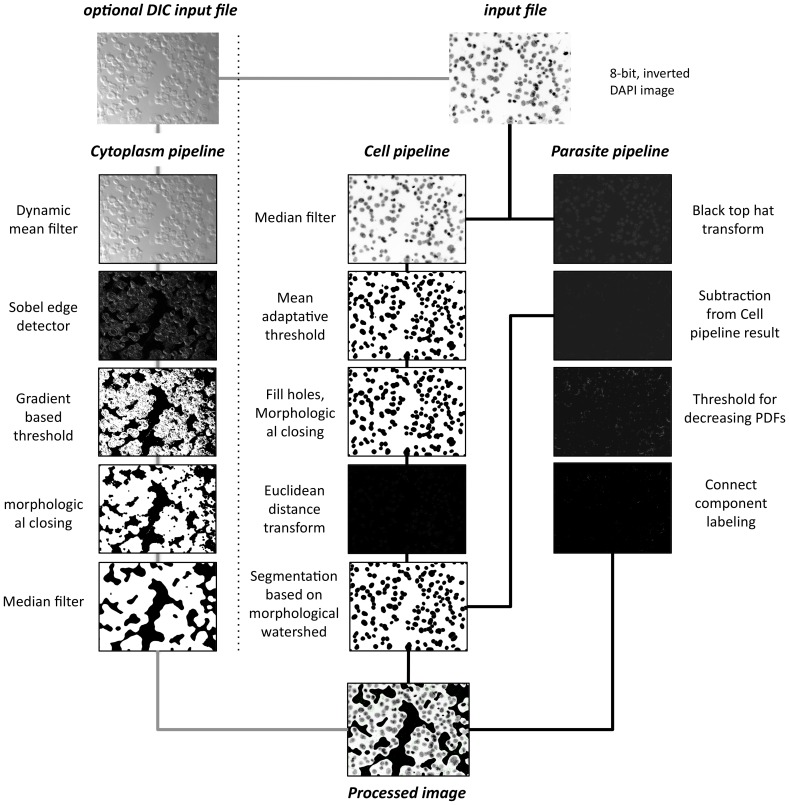
INsPECT processing pipeline. Cell pipeline. Input image is smoothed using median filter of radius 15. Smoothed image is thresholded using Mean Adaptive Threshold. Window size is assigned automatically and here it is 65. Holes are filled using Binary Fill Holes algorithm and cell scraps are removed using Morphological Closing with structure element radius of 15. Image is transformed to Euclidean Distance domain. Morphological Watershed based segmentation is used to separate overlapped cells and quantify final valid cells. **Parasite Pipeline.** Top Hat transform is applied to input image to extract tiny details. Cell Pipeline resulted image is subtracted from the current image to filter out tiny pixels belonging to cell portion. Proposed threshold is applied to filter out random noises and other by-products and keep candidates alive for real parasites. Foreground pixels are grouped using Connected Component Labeling algorithm and using minimum and maximum valid sizes taken from user, final valid parasites are extracted from images. **Cytoplasm Pipeline.** Using Dynamic Mean Filter raw, phase contrast or DIC image is smoothed. Sobel Edge Detector operator is used to extract probable edge candidates for cytoplasm traces. Proposed Cytoplasm threshold is used to discriminate weak from strong edge pixels and obtain probable cytoplasm pixel candidates. Morphological Closing with rectangular structure element of size 15 is applied to fill the holes and Merge Cytoplasm binary regions. Binary Median Filter of radius 25 smoothes cytoplasm regions. **Processed Image.** Cytoplasm pipeline result will be masked to input DAPI image and then cell and parasites are marked in the image using cell and parasite pipeline results respectively.

The image-processing pipeline for cell, parasite and cytoplasm detection is illustrated in figure 1 and detailed below. Acquired fluorescent images first need to be saved in tiff format, 8bit type and inverted to obtain dark objects in white backgrounds. These steps can be performed directly from the acquisition software, or lately by different image analyzing softwares. A simple procedure with ImageJ software is described in INsPECT user manual (File S1).

#### 1.1 Cell pipeline

Using Median Filter [16] raw input images are smoothed to remove possible noises. Kernel size is chosen small enough (15) to preserve cells' edge data. The next step is binary thresholding cells' areas. Since input images of this step still suffer from illumination inhomogeneity, the local mean adaptive threshold [17] is an appropriate method. Furthermore, considering the fact that the bright spots in such images have similar intensities and at the same time very different ones from cell portions, as a model, we choose value 1.5 times global standard deviation as a representative kernel window size. Under such assumption, subimages are fed with enough foreground and background pixels. For the illustrative figure, kernel size would be 134. The next step consists of filling small holes using binary fill algorithm [18], and cell scraps are removed using the morphological closing [19] operator. The structuring element is chosen to be a rectangular one with size 15 that is an approximation of area size where high density parasites exist. To quantify cell nuclei areas, a major issue is to detect overlapped nuclei and make borders between them clear. For this purpose, euclidean distance transform [20] is first applied to the last image. Then using the state of the art segmentation and quantification method proposed by Shahbazkia et. Al. [21], the result of pipeline is taken in which overlapped nuclei cells are well discriminated and information such as center coordinates, area and volume are extracted.

#### 1.2 Parasite pipeline

Parasites nuclei are very small particles frequently concentrated around nucleus of infected cells. Their shape and size makes them very similar to random generated noises through raw images. Considering that investigating small details such as parasites in literature are seldom or not precise enough, we propose a new method here, which thresholds parasites with maximum accuracy and we called it Threshold for images with Decreasing Probability Density Function. First, Black Top Hat transform [22] is applied to extract tiny details. Structuring Element size is chosen to be 2, which is in harmony with small size of parasites we want to suppress. The outcome will be a dark image with potential candidates of parasites as brighter pixels. The resulting image will then be subtracted from the Cell Pipeline result image to filter out bright pixels which belong to cell areas. At this point, the algorithm should be able to distinguish between weak and strong parasite candidates and consequently, filter out random generated noises and keep real parasites alive. In order to accomplish this task, we execute the following steps: (i) Calculate the Global Mean Intensity (m) and Global Standard Deviation (stDev) of the input image. (ii) Pixels of the original image that have greyscale intensities equal to or below m+stDev are set to background (BG) in result image and also labeled in original image as BG. (iii) For pixels of the original image which have greyscale intensities greater than m+stDev: if such pixels in their 8-connected neighborhood have at least 8/2+1 = 5 BG pixels, then set them to background. Otherwise set them to foreground. This algorithm discriminates real parasites from random noises not only using the intensity features of each pixel but also by the organization of its neighbors. Brighter pixels with maximum likelihood to be real parasites are extracted. Using connected component labeling [23] algorithm we divide them to connected components and then, using minimum parasite size and maximum parasite size, taken from user, final parasites are extracted and recorded through the pipeline.

#### 1.3 Cytoplasm pipeline

Cell edges including cytoplasm can be easily visible from DIC and phase contrast images. We decided to use these characteristics to design an algorithm which detects cells' boundaries and discriminate intracellular from extracellular parasites. The Gaussian Noise [24] model is first assigned to raw input DIC images and using dynamic mean filter [25], the degradation process takes place to remove noises and smooth images to a certain degree. Given that membrane edges do not have well defined patterns and that illumination across the borders show high variations, global and local intensity based thresholds are not able to perform efficiently on such images. We therefore proposed our method called Gradient Based Threshold method, which produces a binary image with potential cytoplasm pixels as foreground and the remainder pixels as background. To accomplish this task, we first use Sobel Edge Detector [26], which extracts edges or equivalently pixels which have noticeable illumination variation. The algorithm uses a first estimation of binary threshold as an edge pixel strength measure. It then goes through the image, pixel by pixel. If a pixel or if at least one of its neighbor in a defined neighborhood (8-connected for our problem) has intensity above the intensity measure, then the algorithm marks the pixel as foreground, otherwise it is marked as background. The intensity measure should not be unrepresentative of the discrimination point and meanwhile it should not overestimate or underestimate the strong pixels (or foreground). Experiments showed that Global Mean Intensity value is occasionally a good choice for this parameter since strong parts of the edges usually have intensities above this value, weaker parts have some neighbors above this value and weakest parts along with its neighbors totally lie under this value. In order to complete the task of extracting cytoplasm regions, Morphological Closing (with rectangular structuring element of size 15) is used to fill and merge structures of extracted cytoplasm, then, using Median Filter [27] with a relatively big kernel size (25), small holes are filled, small detected portions are discarded and the final result is smoothed.

#### 1.4 Output data

All analyses are regrouped into an output folder that contains output annotated image(s) and a .CSV format report with all calculated infection parameters including: (i) total number of cells, (ii) total number of parasites, (iii) percentage of infected cells, (iv) mean number of parasites by cell, (v) the parasitic index (calculated as PI = percentage of infected cells x mean number of parasites by cell). Cells that have at least one intracellular parasite are considered as infected cells. To assign each detected parasite to its relevant cell, the euclidean distance between the center of that parasite to each cell is calculated and the cell with minimum distance is chosen to be the pair cell for that parasite. In the output image file, each parasite is therefore assigned with the same number as its corresponding host cell. By comparing each parasite's center with borders of extracted cytoplasmic regions, we then subdivide total parasites into intra- and extracellular parasites.

#### 1.5 Software homepage and download

INsPECT software and the associated source code can be downloaded from the IRD bioinformatics homepage: http://bioinfo.mpl.ird.fr/index.php

### 2. Biological material and infection procedures

#### 2.1 Glucantime action on THP-1 infected cells with *L. infantum* parasites

*L. infantum* (MHOM/MA/67/ITMAP-263) promastigote parasites were maintained at 26°C in SDM-79 medium [28] supplemented with 10% heat-inactivated fetal calf serum (FCS) (Lonza). A human monocyte leukemia cell line (THP-1) was cultured in RPMI 1640 medium (Lonza) supplemented with 10% FCS, 2 mM glutamine, 100 IU penicillin ml-1 and 100 mg streptomycin ml-1, and incubated at 37°C with 5% CO2. Parasite infection was performed according to the method adapted from Da Luz et al. [29]. Briefly THP-1 cells in the log phase of growth were differentiated in a 16-chamber slide (Labtek) at a concentration of 5.10^4^ cells/well by incubation for 1 day in RPMI medium containing 20 ng of phorbol myristate acetate (PMA) per ml. To optimize cell infection, five-day-old promastigote cultures were transferred to acid Schneider's medium (Lonza) (pH 5.4) supplemented with 20% FCS, and incubated for 24 h at 26°C. After one wash in RPMI, parasites were put in contact with macrophages (10 parasites for 1 macrophage ratio) for 24 h. Non-internalized parasites were removed by five washes with serum free RPMI medium. Fresh complemented medium with different amounts of glucantime (ranging from 0 to 100 µg/ml) is then added for 4 subsequent days of incubation. Cells were then fixed 2 minutes in methanol and slides were stained for 20 minutes in the dark with Giemsa (20% in H20) or DAPI nucleic acid stain (300 nM in PBS). For DAPI, slides were washed twice with PBS, mounted with Prolong (Molecular Probes) antifade reagent and stored in the dark at 4°C. Images were captured on a Zeiss Axio Imager AX10 microscope equipped for DAPI (405 nm) excitation and ×20, ×40, ×63 objectives. Ec50 was calculated using Graphpad Prismv5 software (sigmoidal dose-response model with a variable slope).

#### 2.2 HFF fibroblasts infected by *T. gondii*

*T. gondii* proliferation was analyzed as described hereafter. 5.10^4^ YFP-expressing RH parasites/well (48 well plate) were centrifuged on confluent HFF monolayers for 30 sec at 1300 rpm and incubated for 30 min in a water bath at 37°C for invasion. Wells were then washed three times with PBS to eliminate extracellular parasites and medium. After 24 h at 37°C in a humidified atmosphere containing 5% CO_2_, cells were fixed in 2.5% formaldehyde (methanol free)/PBS for 30 min at room temperature. Nuclei were stained with Hoechst 33258 (2 µg/ml) for 20 min and then washed three times 10 min with water. Image acquisition was performed on Olympus Scan∧R microscope using Scan∧R software and ×20 objective.

## Results

### 1. Software presentation

**Figure 2:**
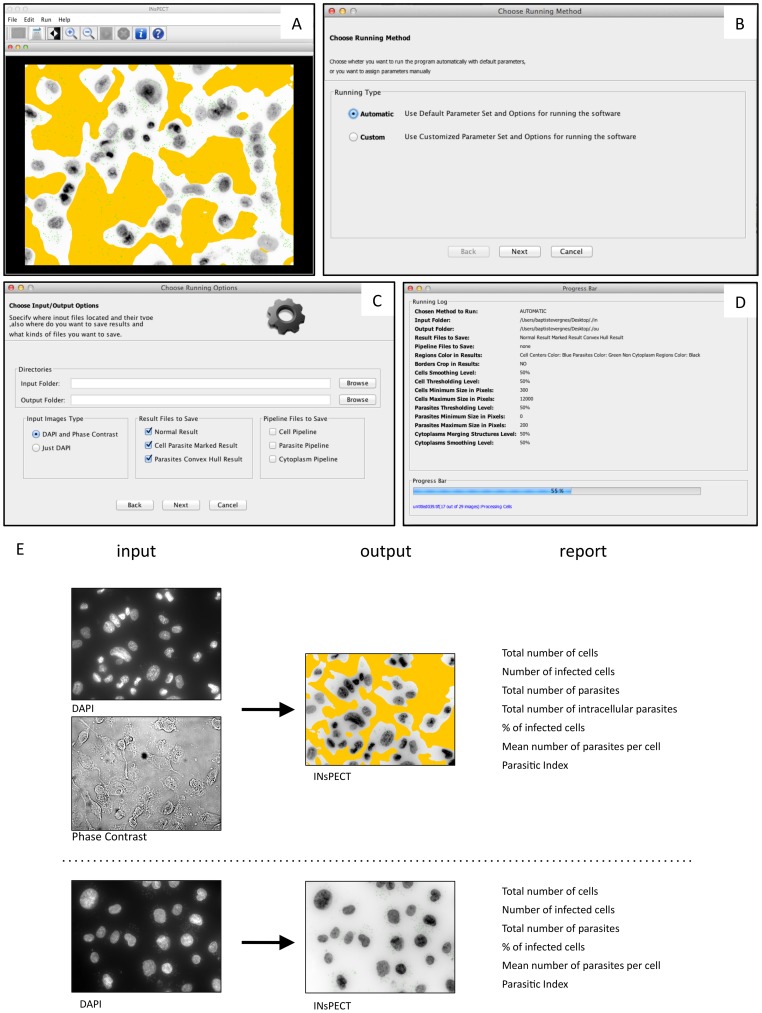
INsPECT presentation: Screen shots of the software interface and graphical abstract of possible analyzes. (A) Main software's frame to control flow of analyzes. Software comes with a basic functional image viewer. (B) Software can be used in automatic or custom fashion. In the former case, all pipeline parameters and options are assigned automatically to image sets based on prior good experimental results while in the latter case, user guides the program with his intended parameters and options. (C) Input/Output window to specify intended options to save the results and reports. (D) Running log and progress bar sample. (E) Graphical abstract of the different type of analyses and the corresponding output image and text results.

Screen shots of the INsPECT interface are shown in Figure 2, all frames are designed in such a way that a non-experimented user in image processing can easily work with them. A complete explanation of the software utilization can be found in the INsPECT user manual (File S1). The software comes with a basic functional image viewer that helps to see particles in images easily (Figure 2A). Software can run either in an automatic or in a custom mode (Figure 2B). In the former case, parameters and options are set to some default values that are in agreement with the nature of most input images; however, end users can also adapt their intended parameters and options for cell, parasite and cytoplasm pipelines manually (see details in user manual). Users should then indicate input and output folders used for the rest of the analysis, and the nature of the images to analyze (Figure 2C). In the same window, users further specify which result files need to be saved. A running log and progress bar is displayed during the analysis (Figure 2D). Basically, the software takes images from the input folder, applies proposed algorithmic pipeline to each image, extracts needed information and finally saves the results in both text and visual forms in the specified output folder. If DAPI and phase contrast image pairs are available, the software extracts cell outlines to distinguish intra from extracellular parasites (Figure 2E). An example of the accuracy of the proposed cell edges detection algorithm is illustrated in figure S1 for both phase contrast and DIC microscopic images. Alternatively, when DIC images are not available or of minor importance in final results, the software can deal with DAPI images only (Figure 2E). The processing of each image takes less than 30 seconds, without any need of user interaction.

### 2. INsPECT software validation *versus* Giemsa stain: Susceptibility of intramacrophagic *L. infantum* toward glucantime

**Figure 3:**
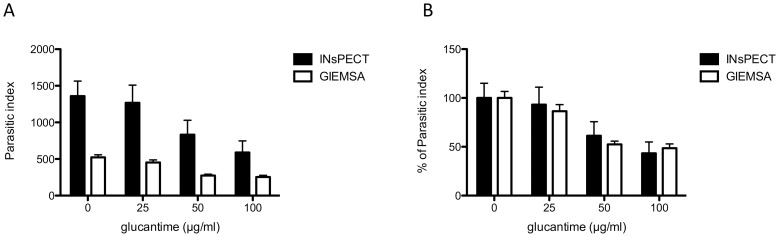
Comparison of parasitic index values obtained for both INsPECT and Giemsa methods. (A) Histogram of parasitic index values obtained in table 1. (B) Normalization of graphic in (A) representing the percent reduction of the total parasite burden compared to the non-treated infected control.

To validate the software accuracy in detecting intracellular *Leishmania* parasites, we performed two parallel experiments with *L. infantum* infected macrophage THP-1 cell line treated by increasing concentration of glucantime (0-25-50-100 µg/ml), the mainstay treatment for leishmaniasis. After infection, drug incubation and cell fixation, one series was stained with Giemsa for manual counting and the other with DAPI, both procedures taking approximately similar time (30 minutes). In the manual counting, 300 cells (100 for each triplicate) were counted under light microscope for each drug concentration to determine the parasitic burden (parasitic index), calculated as the percentage of infected macrophages x the mean number of amastigotes per macrophage (Table 1). In parallel, random DAPI images were acquired with fluorescent microscope (40× objective) until approximately 300 cells were reached by condition (corresponding to 31 images and 1402 cells in total), together with their respective phase contrast images for cytoplasm detection (File S2). A batch analysis of all images was performed with INsPECT software (automatic parameters) and was completed in less than 7 minutes (Table 1). Direct comparison of output results shows that the number of detected intracellular parasites, and the subsequent calculated parasitic index (PI), is markedly higher with INsPECT software than with Giemsa method (Figure 3A). Verification of the INsPECT analyzed images in the output folder allows us to confirm that the detected particles accurately correspond to true parasites, excluding therefore an overestimation of the parasite load by the software. Conversely, the human operator naturally tends to select fields and cells offering the best visual resolution with Giemsa, generally excluding highly infected cells in which parasites cannot be well discriminated. The analysis of the “Cell Parasites Report” generated by INsPECT software confirms that highly infected cells are indeed frequently observed in untreated or low glucantime treatment conditions, and that this category of cells is mainly responsible for the differences observed between the two methods (Figure S2).

**Table 1:** Infection report obtained with INsPECT software (L1–L31) and Giemsa counts (bold) toward *Leishmania* infected THP-1 macrophages treated with increasing concentrations of glucantime (25-50-100 µg/ml).

Glucantime (µg/ml)	Total number of cells	Number of infected cells	Total number of parasites	Number of intracellular parasites	% of infected cells	Mean number of parasite/cell	Parasitic index (PI)	File name^a^
**0**	58	52	595	562	89.66	10.81	969.22	L1
	50	47	607	551	94.0	11.72	1101.68	L2
	70	68	844	774	97.14	11.38	1105.45	L3
	38	29	683	637	76.32	21.97	1676.75	L4
	48	48	844	830	100.0	17.29	1729.0	L5
	36	34	479	469	94.44	13.79	1302.33	L6
	33	33	501	437	100.0	13.24	1324.0	L7
	**100**	**75**	**-**	**686**	**75**	**6,9**	**515**	-
	**100**	**81**	**-**	**693**	**81**	**6,9**	**561**	-
	**100**	**73**	**-**	**675**	**73**	**6,8**	**493**	-
**25**	68	66	876	840	97.06	12.73	1235.57	L8
	61	59	657	649	96.72	11.0	1063.92	L9
	74	70	799	788	94.59	11.26	1065.08	L10
	40	37	407	345	92.5	9.32	862.1	L11
	66	61	887	883	92.42	14.48	1338.24	L12
	58	53	633	615	91.38	11.6	1060.01	L13
	48	47	771	761	97.92	16.19	1585.32	L14
	47	44	569	537	93.62	12.2	1142.16	L15
	**100**	**71**	**-**	**693**	**71**	**6,93**	**492,03**	-
	**100**	**65**	**-**	**654**	**65**	**6,54**	**425,1**	-
	**100**	**70**	**-**	**628**	**70**	**6,28**	**439,6**	-
**50**	28	27	297	244	96.43	9.04	871.73	L16
	22	21	184	161	95.45	7.67	732.1	L17
	44	40	648	423	90.91	10.58	961.83	L18
	27	24	428	310	88.89	12.92	1148.46	L19
	36	28	211	205	77.78	7.32	569.35	L20
	27	23	277	177	85.19	7.7	655.96	L21
	30	26	190	165	86.67	6.35	550.35	L22
	49	45	458	399	91.84	8.87	814.62	L23
	**100**	**58**	**-**	**469**	**58**	**4,69**	**272,02**	-
	**100**	**60**	**-**	**429**	**60**	**4,29**	**257,4**	-
	**100**	**63**	**-**	**465**	**63**	**4,65**	**292,95**	-
**100**	60	52	419	364	86.67	7.0	606.69	L24
	40	38	372	262	95.0	6.89	654.55	L25
	19	16	142	133	84.21	8.31	699.79	L26
	43	35	235	216	81.4	6.17	502.24	L27
	49	44	315	294	89.8	6.68	599.86	L28
	47	40	264	214	85.11	5.35	455.34	L29
	39	29	161	142	74.36	4.9	364.36	L30
	47	39	210	193	82.98	4.95	410.75	L31
	**100**	**57**	**-**	**466**	**57**	**4,66**	**265,62**	**-**
	**100**	**59**	**-**	**455**	**59**	**4,55**	**268,45**	**-**
	**100**	**56**	**-**	**406**	**56**	**4,06**	**227,36**	**-**

a Image files L1 to L31 can be found in File S2.

Once normalized to the non-treated infected control however, scaled PI values are very similar with calculated Ec50 of 73.97 µg/ml (95% confidence interval [CI], 60.31 to 90.71) and 79.53 µg/ml (95% confidence interval [CI], 54.7 to 115.6) for INsPECT and Giemsa methods, respectively (Figure 3B).

### 3. INsPECT software validation toward intracellular *T. gondii* parasite

**Figure 4:**
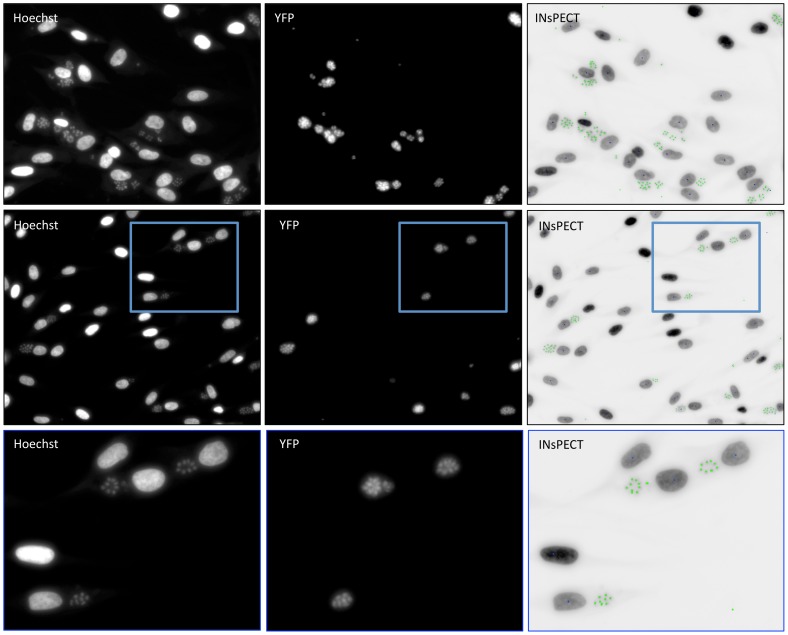
INsPECT output image analysis of *T. gondii* infected HFF cells. Representative images of *T. gondii* infected cells with Hoechst stain (left) and filter for YFP-expressing parasites detection (center). Analyze of the corresponding Hoechst images with INsPECT software (right), showing annotated parasites and cells as green and blue dots respectively. A close-up of a selected area (blue square) is shown in the lowest panel.

To further validate software robustness against unrelated intracellular parasite, we performed analyses on HFF fibroblasts labeled with Hoechst fluorescent DNA stain and infected by fluorescent YFP-expressing *T. gondii* RH parasites (File S3). All input images were obtained from HCS system designed for fully automated image acquisition and analysis (see details in Methods section). The analysis of representative images was performed with INsPECT software using only DNA fluorescent input images and choosing automatic mode. The size and distribution of *Toxoplasma* parasites strongly differ from that of *L. infantum* amastigotes, with tachyzoites parasites being mostly grouped in small aggregates inside cell cytoplasm. As shown in figure 4, comparison of visual output annotated images to YFP labeled parasites confirms, however, a good correspondence between the two methods.

## Discussion

Monitoring intracellular parasite proliferation is an essential and quotidian activity for many academic or clinical diagnostic laboratories. The majority are still using manual microscopic counting, an unpopular and time-consuming task, that is further highly dependent on operator experience and objectivity thus leading to strong inter-laboratory variability. These facts make intracellular parasites counting a natural assignment for automation. In this study, we describe an innovative image analysis software allowing automatic detection of intracellular parasites and calculation of infection parameters such as the percentage of infected cells, the mean number of parasites per cell or the parasitic index. In a concept comparative study, we show that INsPECT software performs the complete analyze of 1402 *Leishmania* infected cells in less than 7 minutes, while counting the equivalent number of cells under microscope with Giemsa staining can take hours of effective work. This timesaving is furthermore directly proportional to the number of conditions/replicates to analyze. Because images are stored annotated in the output folder, users have the possibility to verify whether recognition of parasites and cells is satisfying enough and can optimize detection parameters accordingly (see user manual, File S1).

The use of common and affordable fluorescent nucleic acid stains such as DAPI or Hoechst present the advantages to display a bright, homogenous and stable labeling of both intracellular parasite and host cell DNA. Furthermore, they allow working under physiological conditions with any wild-type parasite species, including clinical isolates. *Leishmania*, like all members of the kinetoplastid family, possess two large DNA containing organelles (kinetoplast and nucleus). Kinetoplast is an A-T rich DNA structure binding DAPI and Hoechst stains with more affinity [13], [30] and that consequently appears highly brighter than parasite nucleic DNA. Since kinetoplasts occupy very tiny portions of input images, and may be occasionally very similar to random generated noises, the task of extracting them with existing thresholding methods is complex because available methods often work efficiently for the objects with quite noticeable size and shape and investigable intensity functions. For that, we propose a new method which thresholds parasites with maximum accuracy by defining further discrimination steps than just the particle size. DNA structures of all kinetoplastids (including *T. cruzi* and other *Leishmania* species) appear very similar on images stained with DAPI [13], [14]. Accordingly, we expect INsPECT software to be as effective with any other intracellular kinetoplastid parasite as with *L. infantum*. Besides, we confirm that input images of *T. gondii* intracellular parasites acquired with HCS instrumentation could also be efficiently processed, even in automatic mode, proving therefore that INsPECT software is versatile enough to work with a variety of unrelated parasites and host cells.

Regarding host cell nuclei in DAPI images, illumination variance was handled well by firstly, fitting appropriate noise model which removes noise efficiently while preserving precious cell portions' edge data and secondly hiring adaptive threshold with logical and automatic assignment of local window size. Overlapping cells were also detected and separated using state of the art quantification and segmentation approaches. Cytoplasm boundaries of individual cells are visible structures in DIC or phase contrast images and we used these properties to create a new algorithm that automatically detects cell boundaries to discriminate intra- from extracellular parasites. This function allows considering only intracellular parasites in final calculated infection levels. This can be very useful in the case of *Leishmania* promastigotes infection since we experimented that numerous non-internalized parasites may stay attached to macrophages membrane following initial contact, even after extensive washes. Because their DNA will be as well labeled with DAPI stain, they can therefore induce a bias if analyze is based only on DAPI images. With Giemsa, these parasites are simply excluded from the counting by the human operator. In the case of indirect reporter gene assays using microplate readers however, these parasites can be the source of a false positive signal.

In conclusion, we can state that INsPECT software accuracy and effectiveness is at least as reliable as the existing global estimation methods. Considering its flexibility, simplicity and the state of the art perspectives introduced, it could compete with available commercial and academic packages to become a new helpful tool for the scientific community working with intracellular parasites.

## Supporting Information

Figure S1 Example of cell edges detection accuracy using DIC or phase contrast input images. THP-1 infected cells captured in DIC (A) or phase contrast (B) microscopy (20×). (C) and (D): Overlaps of A and B input images with their respective INsPECT output images. The software identifies the zone that is considered extracellular areas with orange color.
(PDF)

Figure S2 (A) Scatter dot plot of the “cell parasites report” from INsPECT analysis representing all recorded cells and their respective number of intracellular parasites for each image (L1–L31). The dot line on the Y axis symbolizes an arbitrary cut-off number of 20 from which enumeration of intracellular parasites by manual counting is becoming challenging. (B) Mean number of parasite per cell obtained in each condition with INsPECT, Giemsa or INsPECT after the exclusion of highly infected cells bearing more than 20 intracellular parasites (INsPECT <20).
(PDF)

File S1 INsPECT software user manual.
(PDF)

File S2 Transformed DAPI and Phase contrast (PC) image set of THP-1 macrophages infected by *L. infantum* parasites, and treated with increasing concentrations of glucantime (0-25-50-100 µg/ml).
(ZIP)

File S3 Representative images of HFF fibroblasts infected by *T. gondii* each coming as a pair with Hoechst fluorescent cell DNA stain (inverted) and the corresponding YFP-expressing RH parasites.
(ZIP)
