# Supplementary figures and images for: INsPECT, an Open-Source and Versatile Software for Automated Quantification of (Leishmania) Intracellular Parasites
Source: PLoS Negl Trop Dis. 2014 May 15;8(5):e2850. doi: 10.1371/journal.pntd.0002850 (PMC4022486; doi:10.1371/journal.pntd.0002850)

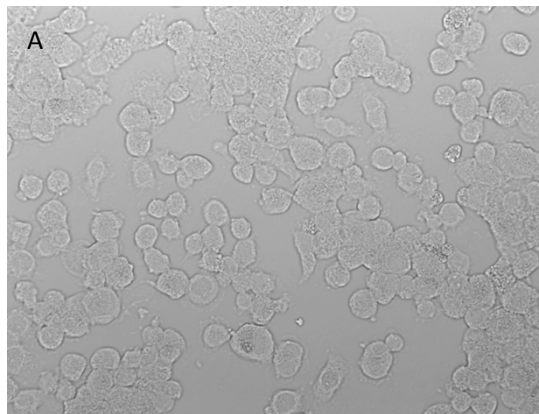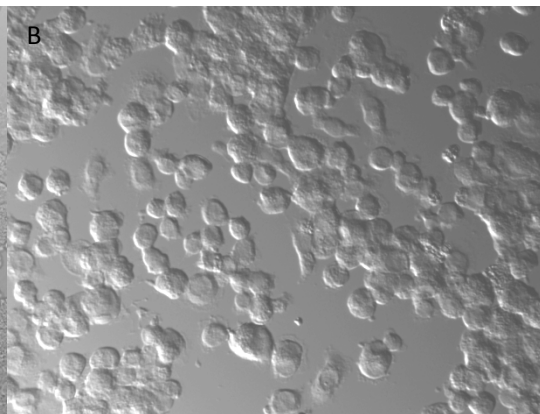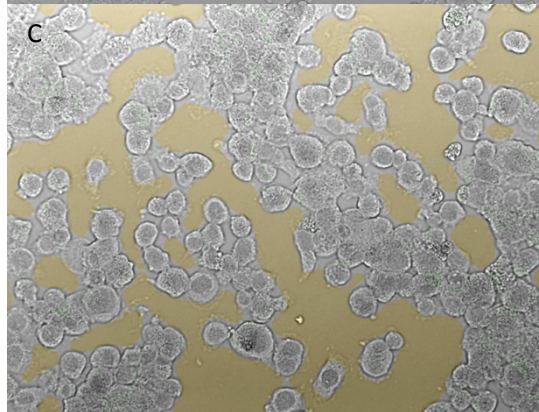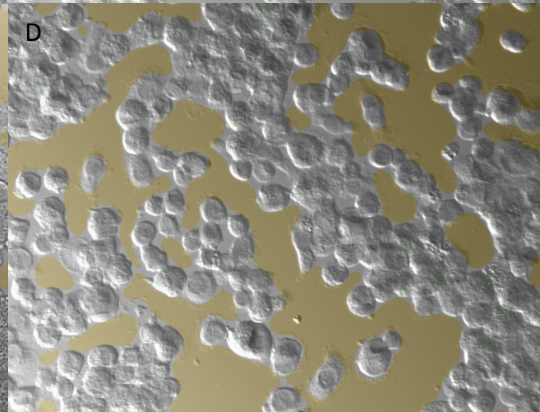

Supplement: Figure S1 — Example of cell edges detection accuracy using DIC or phase contrast input images. THP-1 infected cells captured in DIC (A) or phase contrast (B) microscopy (20×). (C) and (D): Overlaps of A and B input images with their respective INsPECT output images. The software identifies the zone that is considered extracellular areas with orange color. (PDF) [file pntd.0002850.s001.pdf]

**A**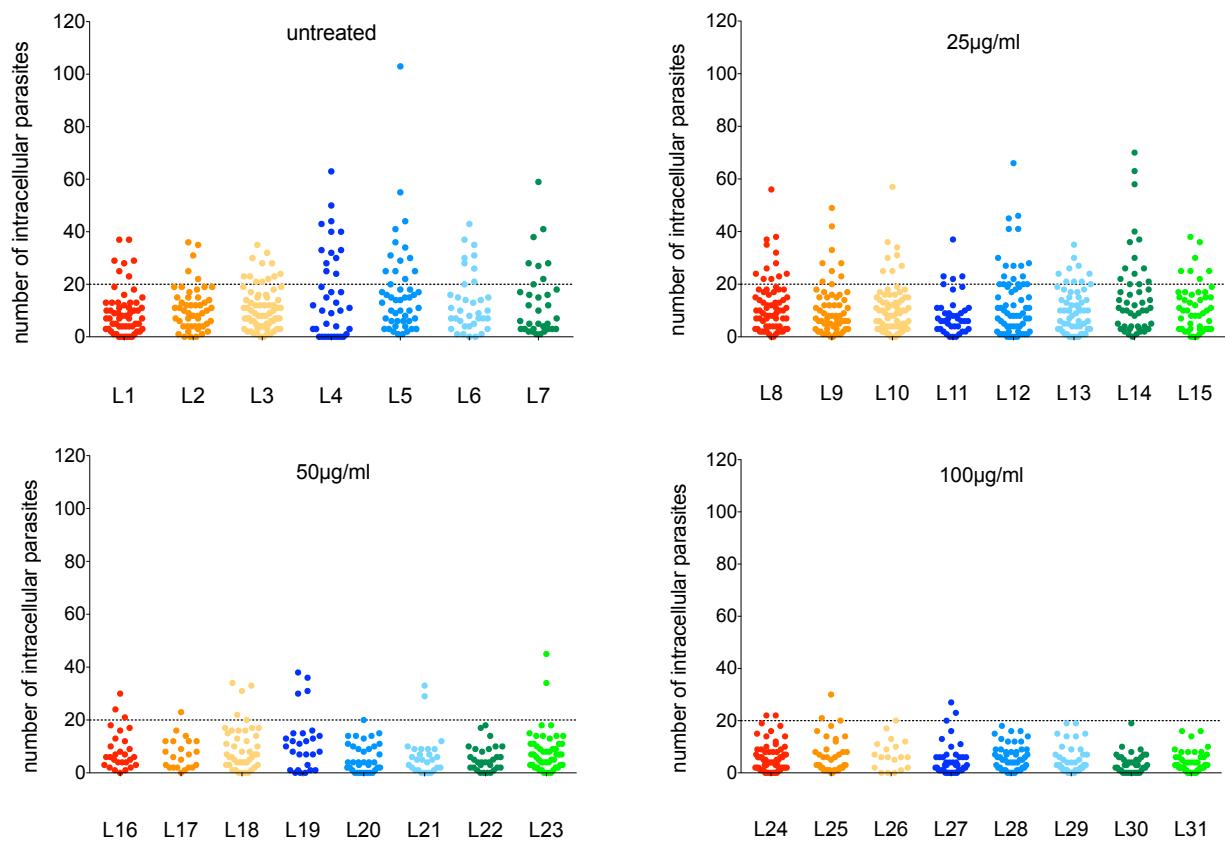**B**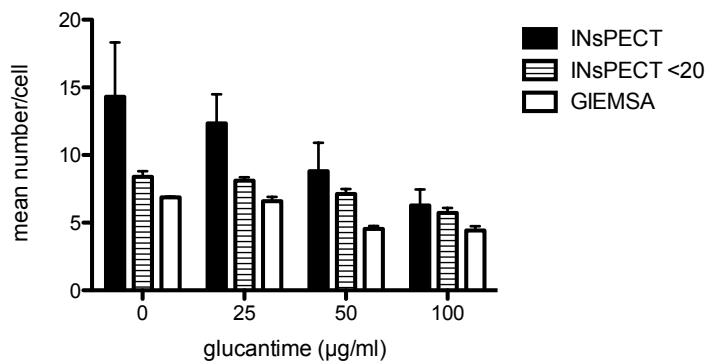

Supplement: Figure S2 — (A) Scatter dot plot of the “cell parasites report” from INsPECT analysis representing all recorded cells and their respective number of intracellular parasites for each image (L1–L31). The dot line on the Y axis symbolizes an arbitrary cut-off number of 20 from which enumeration of intracellular parasites by manual counting is becoming challenging. (B) Mean number of parasite per cell obtained in each condition with INsPECT, Giemsa or INsPECT after the exclusion of highly infected cells bearing more than 20 intracellular parasites (INsPECT <20). (PDF) [file pntd.0002850.s002.pdf]

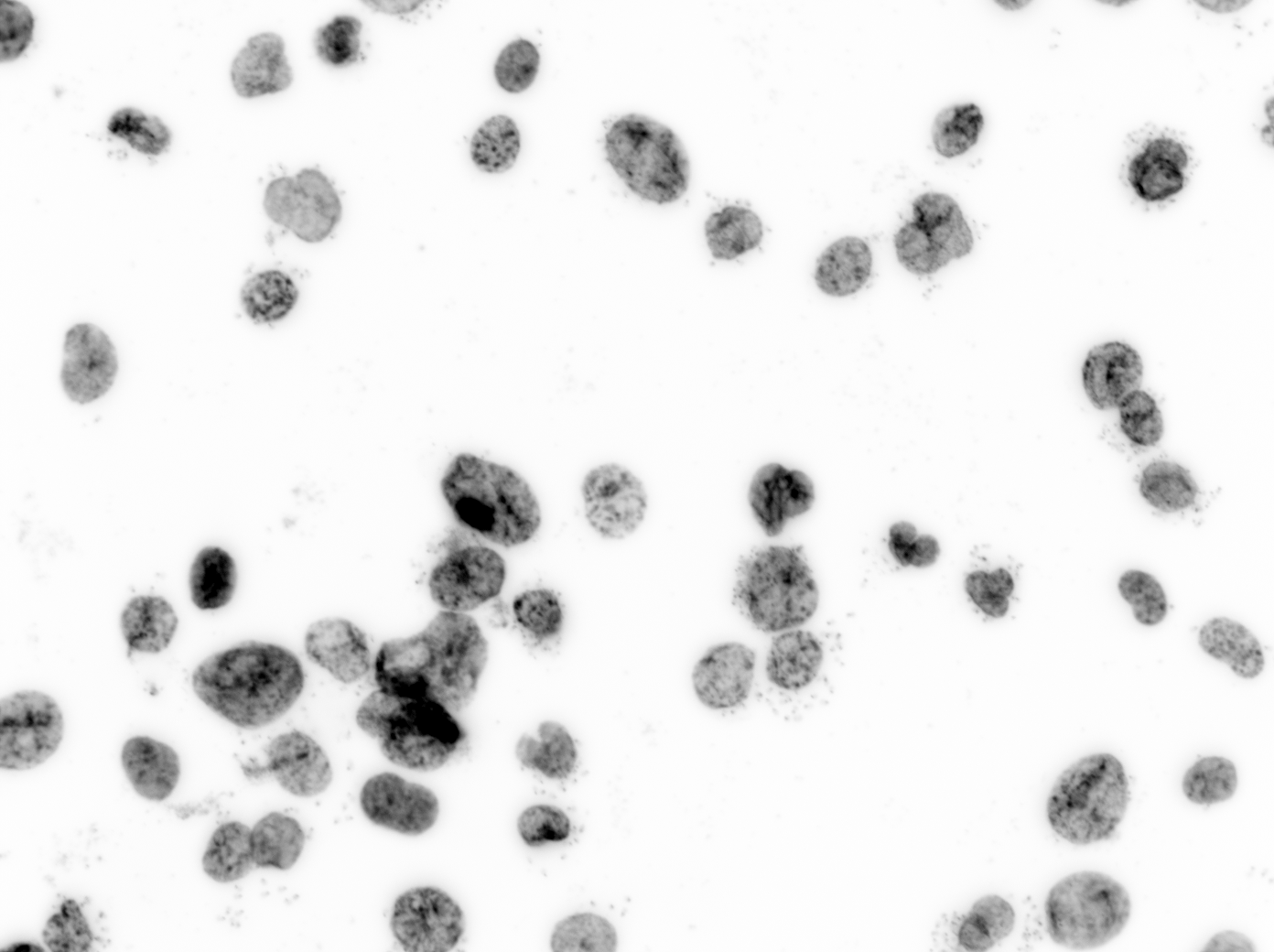

Supplement: File S2 — Transformed DAPI and Phase contrast (PC) image set of THP-1 macrophages infected by L. infantum parasites, and treated with increasing concentrations of glucantime (0-25-50-100 µg/ml). (ZIP) [file pntd.0002850.s004.zip › SI3/100/L24-DAPI.tif]

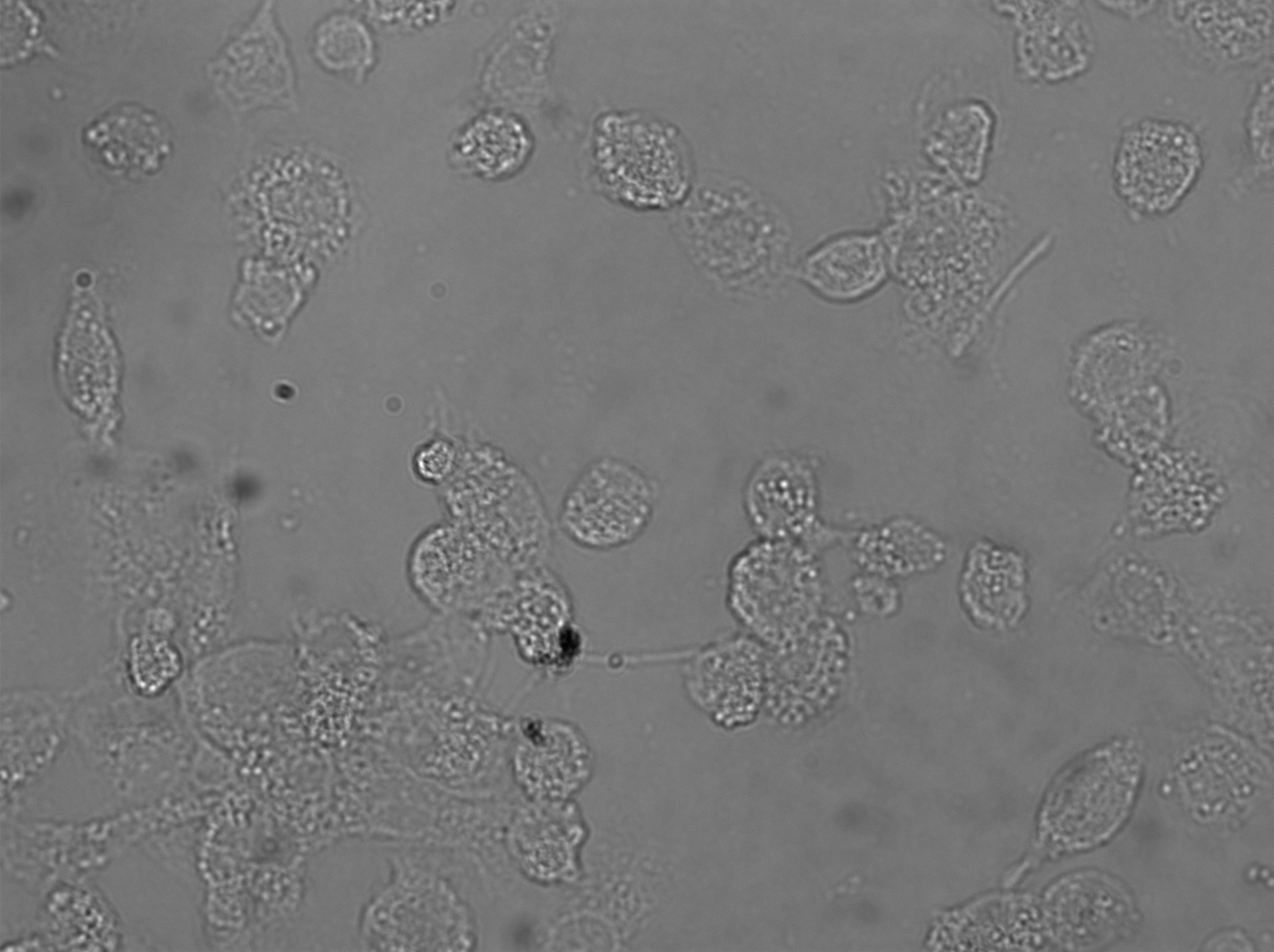

Supplement: File S2 — Transformed DAPI and Phase contrast (PC) image set of THP-1 macrophages infected by L. infantum parasites, and treated with increasing concentrations of glucantime (0-25-50-100 µg/ml). (ZIP) [file pntd.0002850.s004.zip › SI3/100/L24-PC.tif]

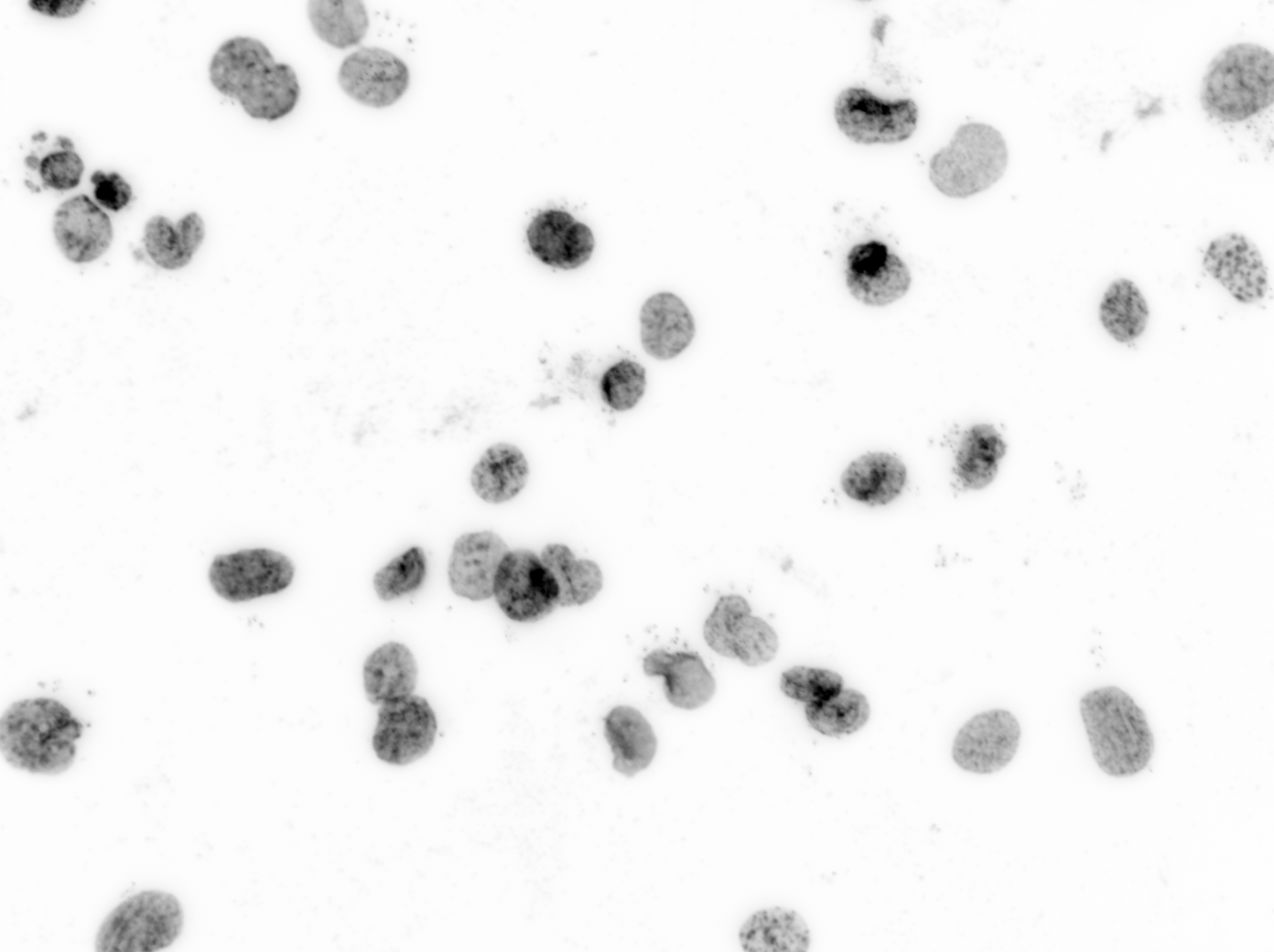

Supplement: File S2 — Transformed DAPI and Phase contrast (PC) image set of THP-1 macrophages infected by L. infantum parasites, and treated with increasing concentrations of glucantime (0-25-50-100 µg/ml). (ZIP) [file pntd.0002850.s004.zip › SI3/100/L25-DAPI.tif]

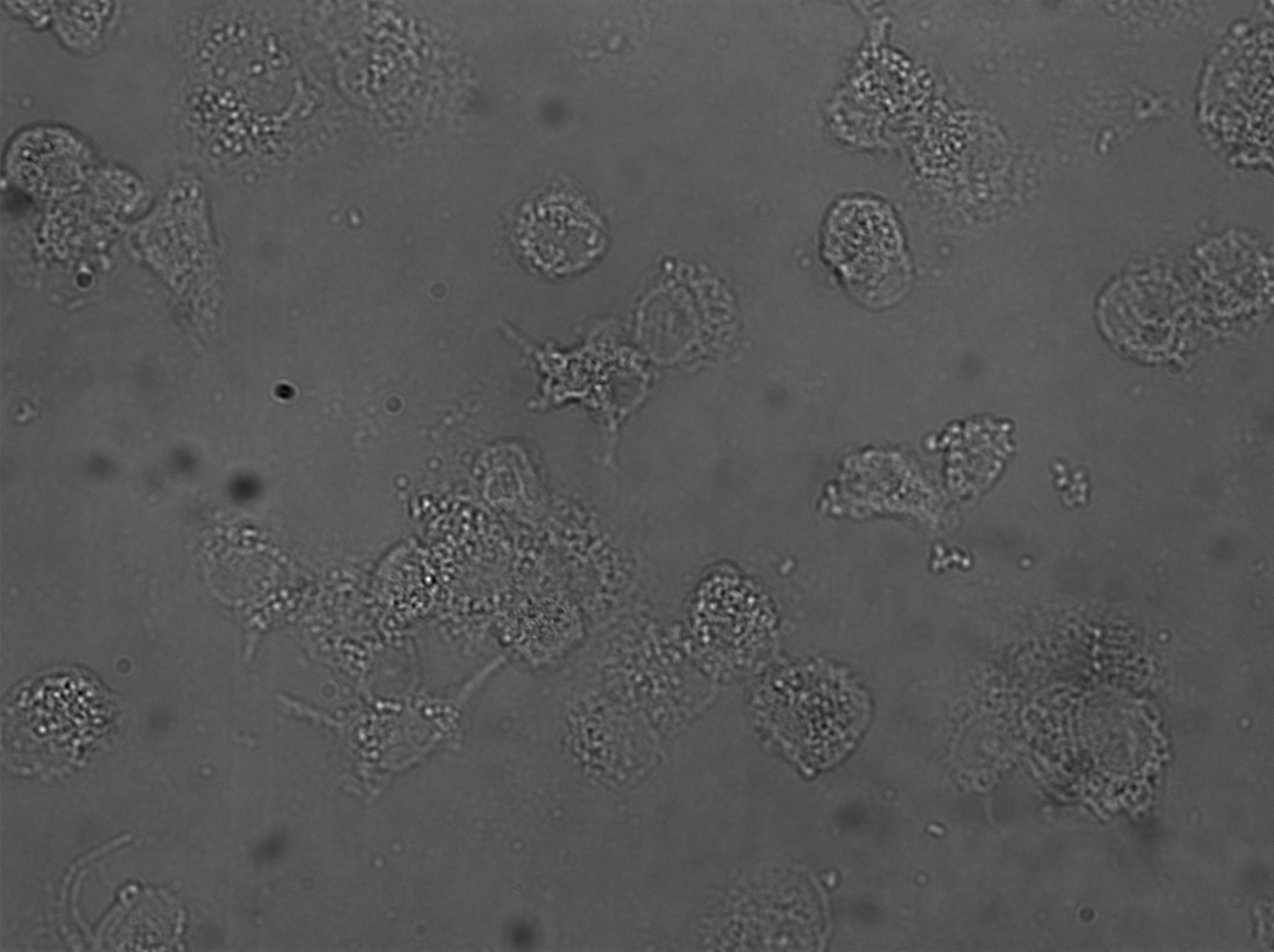

Supplement: File S2 — Transformed DAPI and Phase contrast (PC) image set of THP-1 macrophages infected by L. infantum parasites, and treated with increasing concentrations of glucantime (0-25-50-100 µg/ml). (ZIP) [file pntd.0002850.s004.zip › SI3/100/L25-PC.tif]

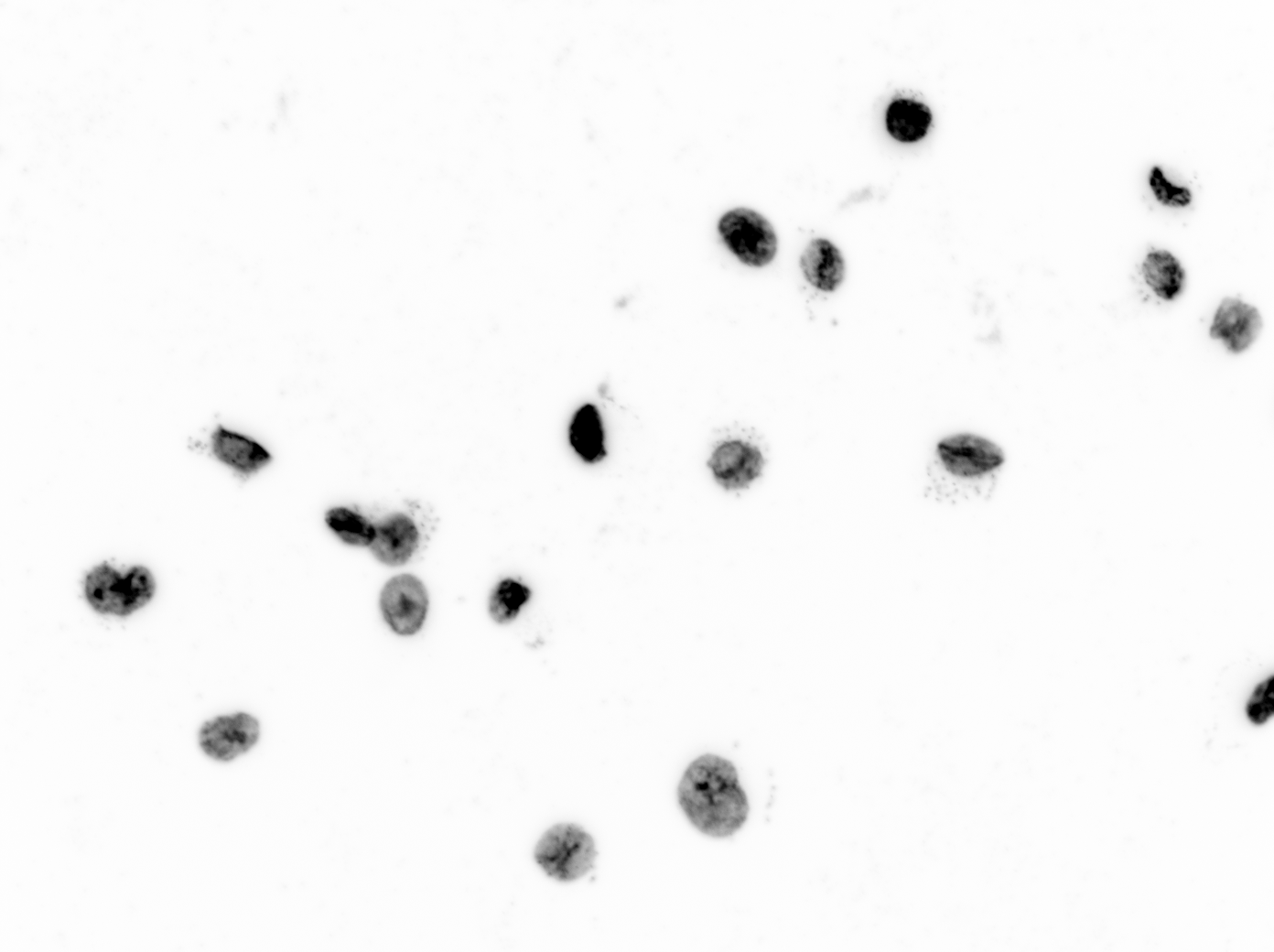

Supplement: File S2 — Transformed DAPI and Phase contrast (PC) image set of THP-1 macrophages infected by L. infantum parasites, and treated with increasing concentrations of glucantime (0-25-50-100 µg/ml). (ZIP) [file pntd.0002850.s004.zip › SI3/100/L26-DAPI.tif]

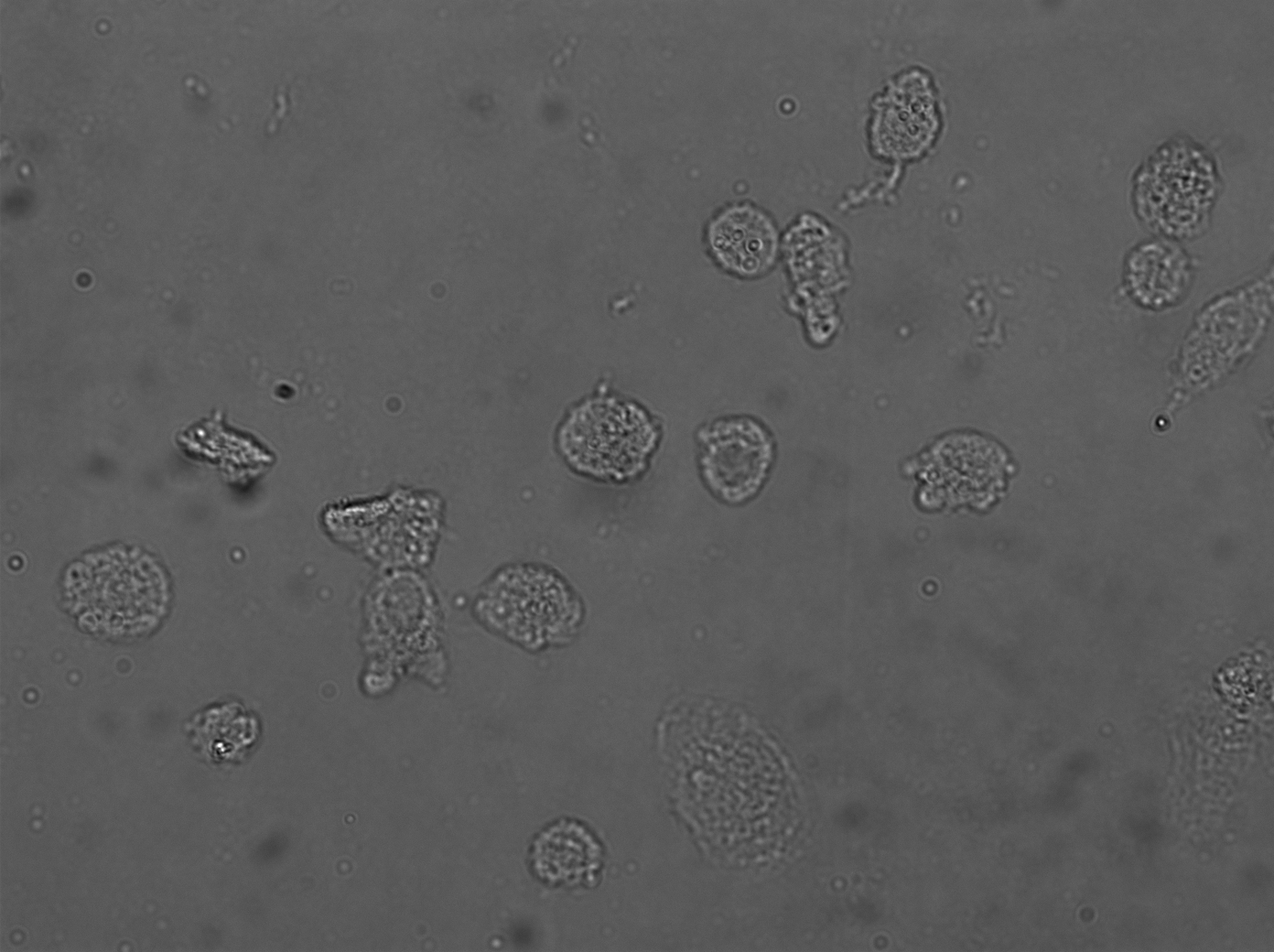

Supplement: File S2 — Transformed DAPI and Phase contrast (PC) image set of THP-1 macrophages infected by L. infantum parasites, and treated with increasing concentrations of glucantime (0-25-50-100 µg/ml). (ZIP) [file pntd.0002850.s004.zip › SI3/100/L26-PC.tif]

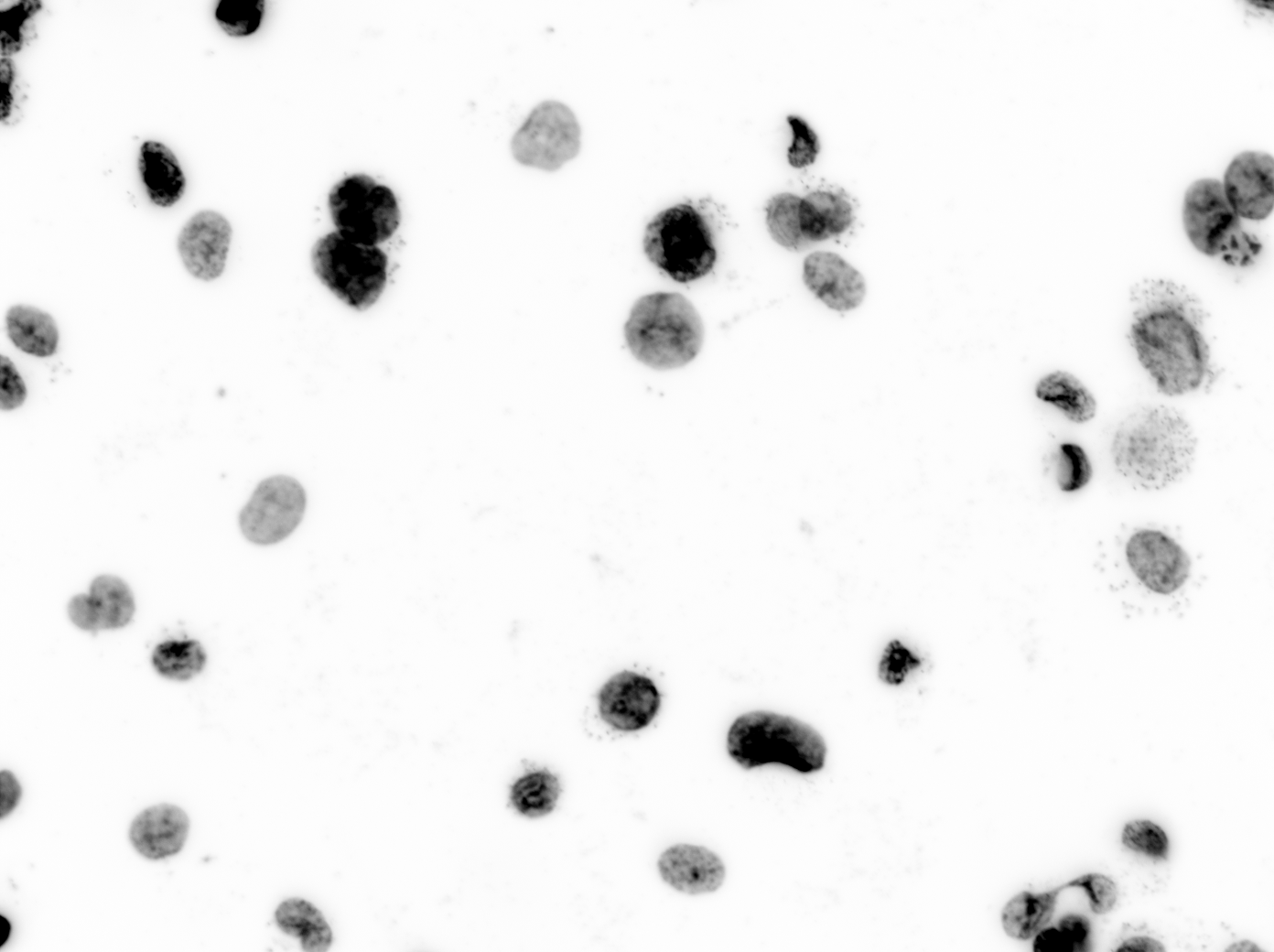

Supplement: File S2 — Transformed DAPI and Phase contrast (PC) image set of THP-1 macrophages infected by L. infantum parasites, and treated with increasing concentrations of glucantime (0-25-50-100 µg/ml). (ZIP) [file pntd.0002850.s004.zip › SI3/100/L27-DAPI.tif]

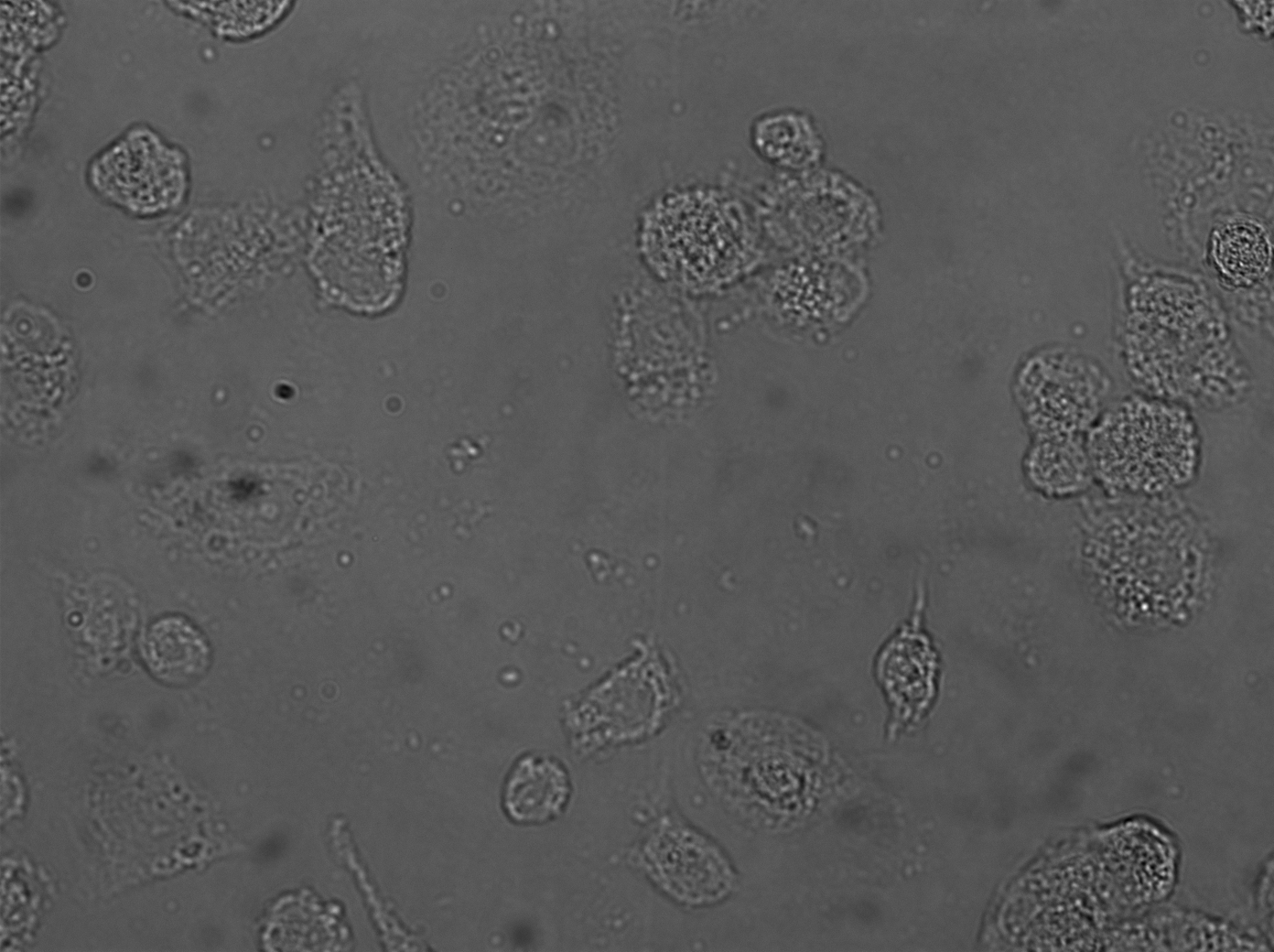

Supplement: File S2 — Transformed DAPI and Phase contrast (PC) image set of THP-1 macrophages infected by L. infantum parasites, and treated with increasing concentrations of glucantime (0-25-50-100 µg/ml). (ZIP) [file pntd.0002850.s004.zip › SI3/100/L27-PC.tif]

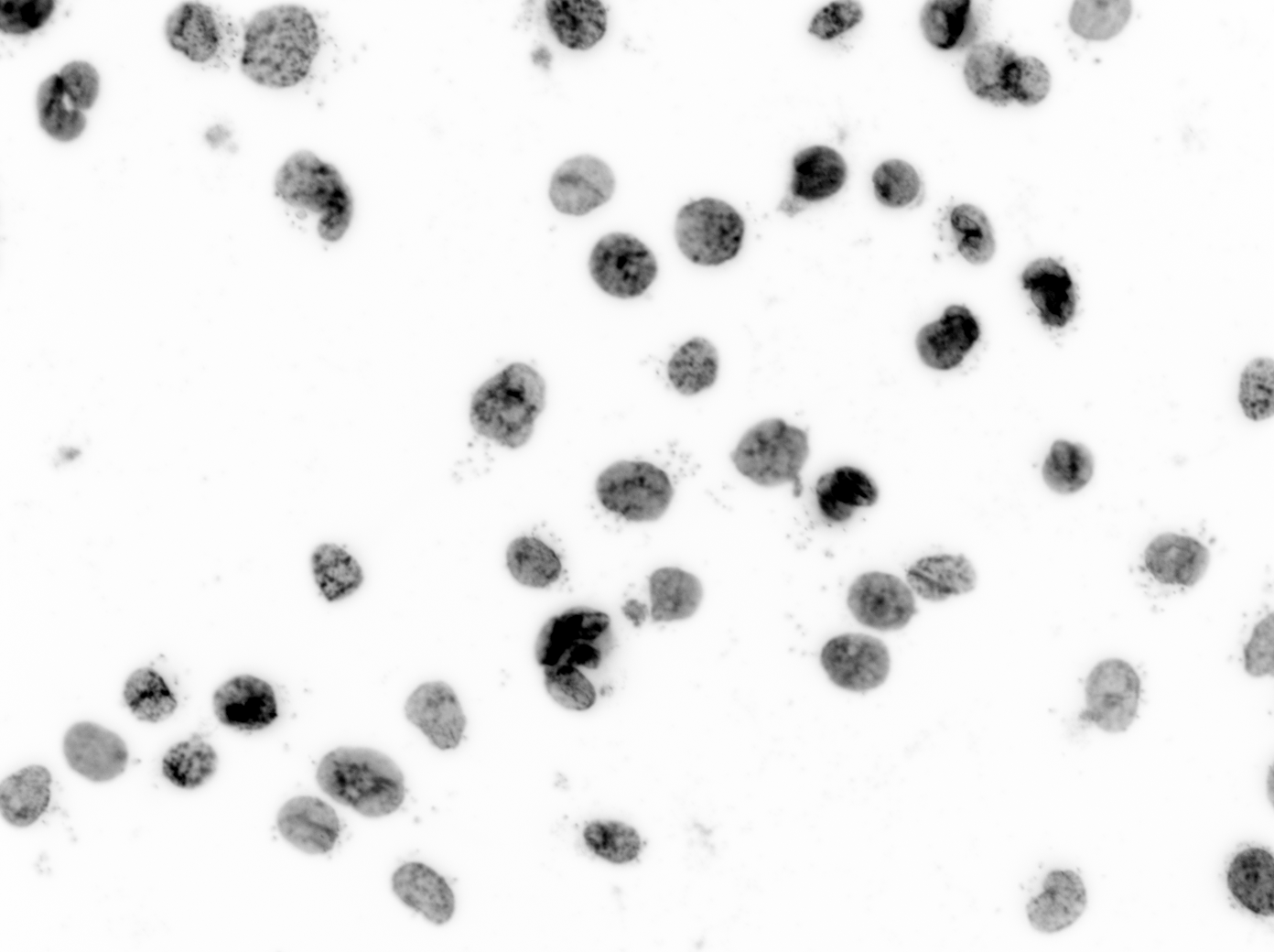

Supplement: File S2 — Transformed DAPI and Phase contrast (PC) image set of THP-1 macrophages infected by L. infantum parasites, and treated with increasing concentrations of glucantime (0-25-50-100 µg/ml). (ZIP) [file pntd.0002850.s004.zip › SI3/100/L28-DAPI.tif]

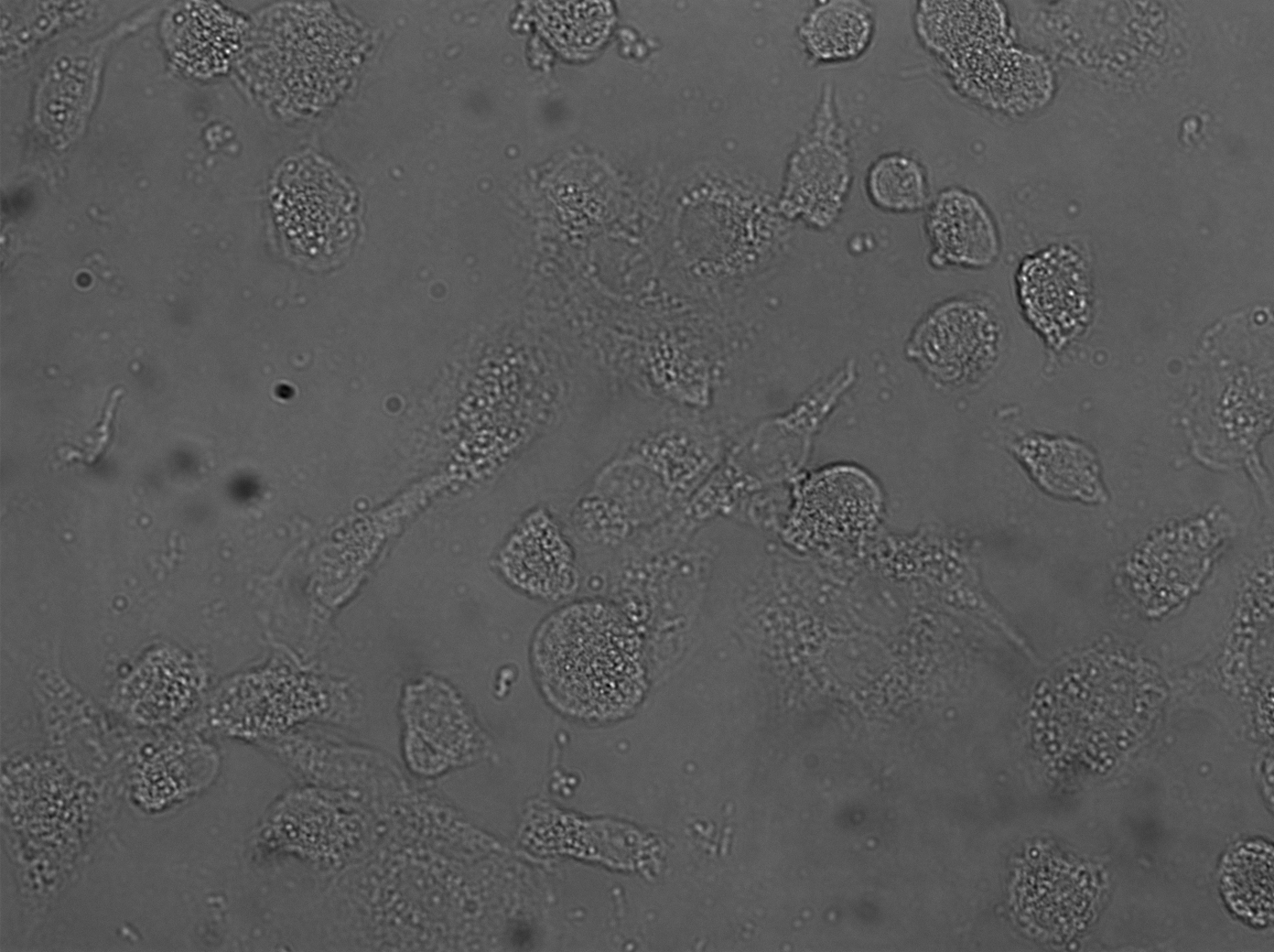

Supplement: File S2 — Transformed DAPI and Phase contrast (PC) image set of THP-1 macrophages infected by L. infantum parasites, and treated with increasing concentrations of glucantime (0-25-50-100 µg/ml). (ZIP) [file pntd.0002850.s004.zip › SI3/100/L28-PC.tif]

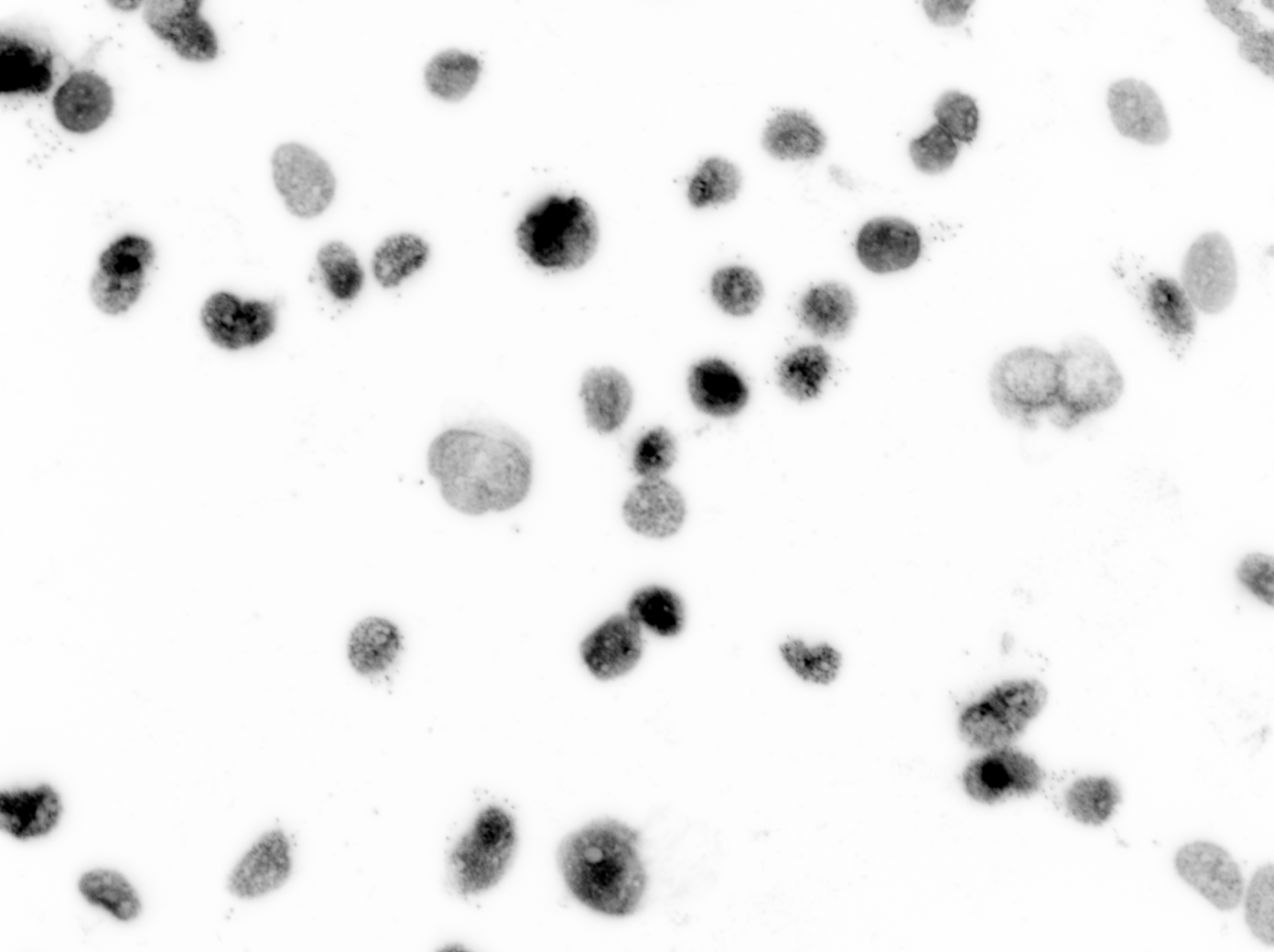

Supplement: File S2 — Transformed DAPI and Phase contrast (PC) image set of THP-1 macrophages infected by L. infantum parasites, and treated with increasing concentrations of glucantime (0-25-50-100 µg/ml). (ZIP) [file pntd.0002850.s004.zip › SI3/100/L29-DAPI.tif]

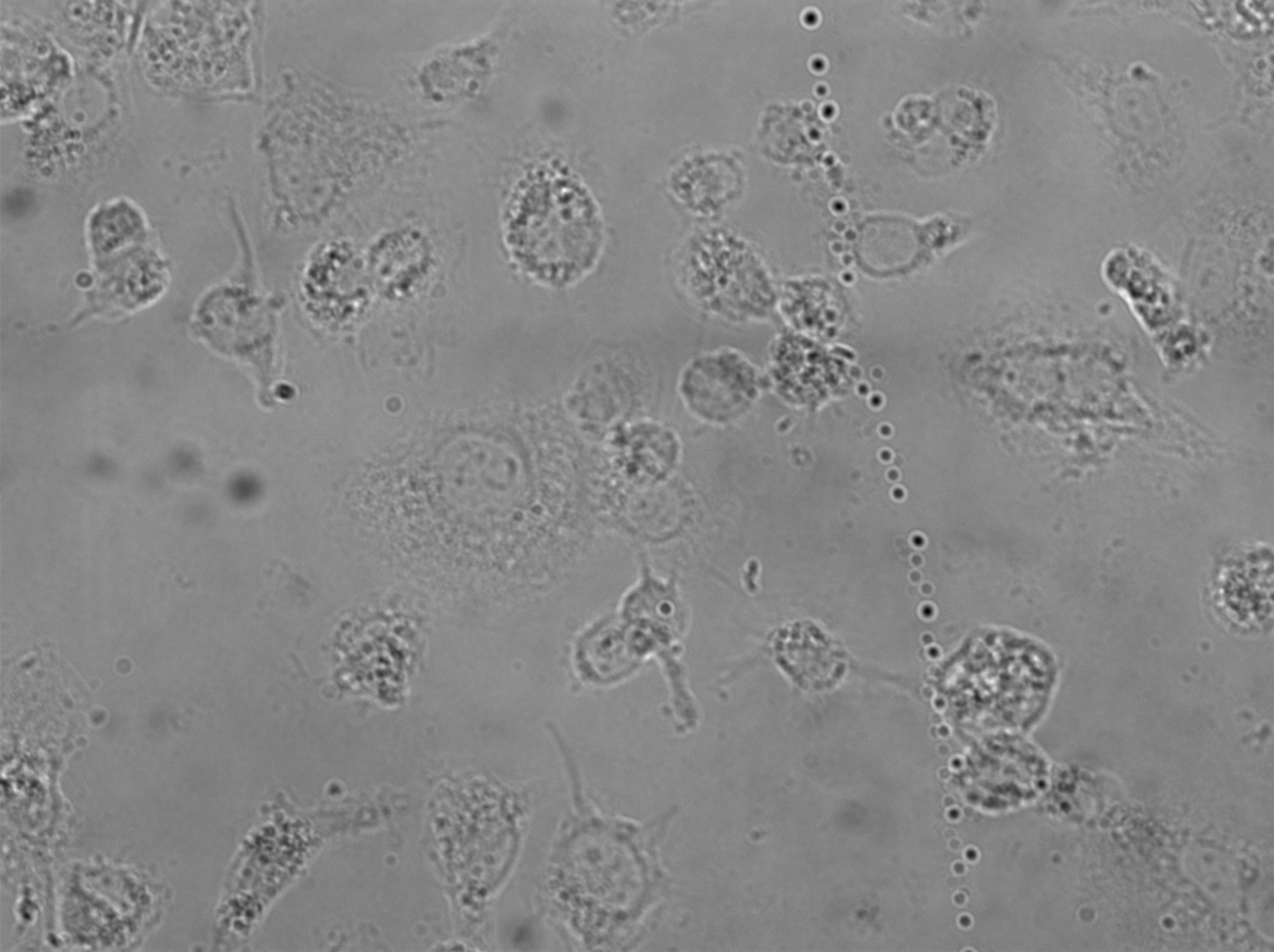

Supplement: File S2 — Transformed DAPI and Phase contrast (PC) image set of THP-1 macrophages infected by L. infantum parasites, and treated with increasing concentrations of glucantime (0-25-50-100 µg/ml). (ZIP) [file pntd.0002850.s004.zip › SI3/100/L29-PC.tif]

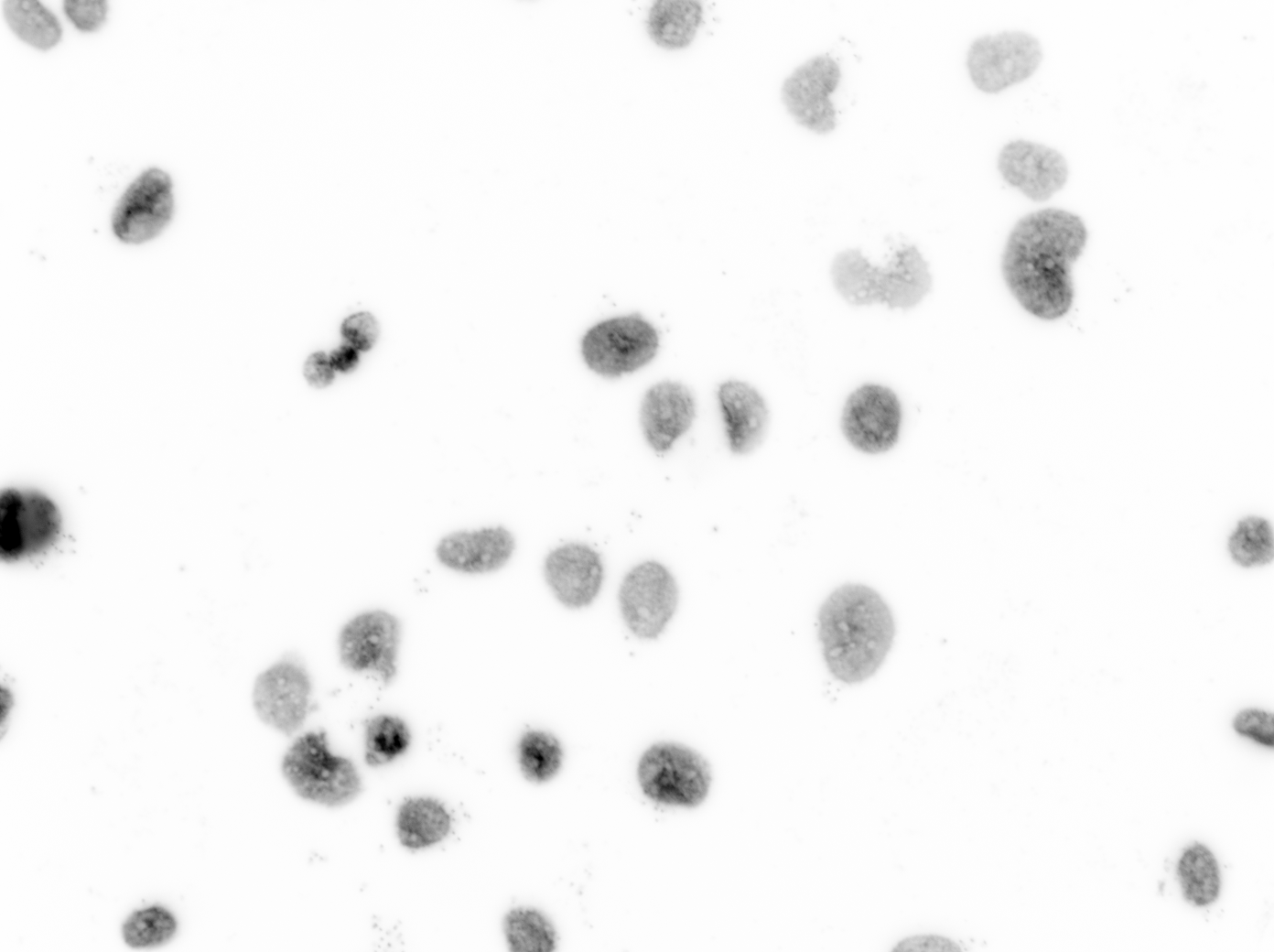

Supplement: File S2 — Transformed DAPI and Phase contrast (PC) image set of THP-1 macrophages infected by L. infantum parasites, and treated with increasing concentrations of glucantime (0-25-50-100 µg/ml). (ZIP) [file pntd.0002850.s004.zip › SI3/100/L30-DAPI.tif]

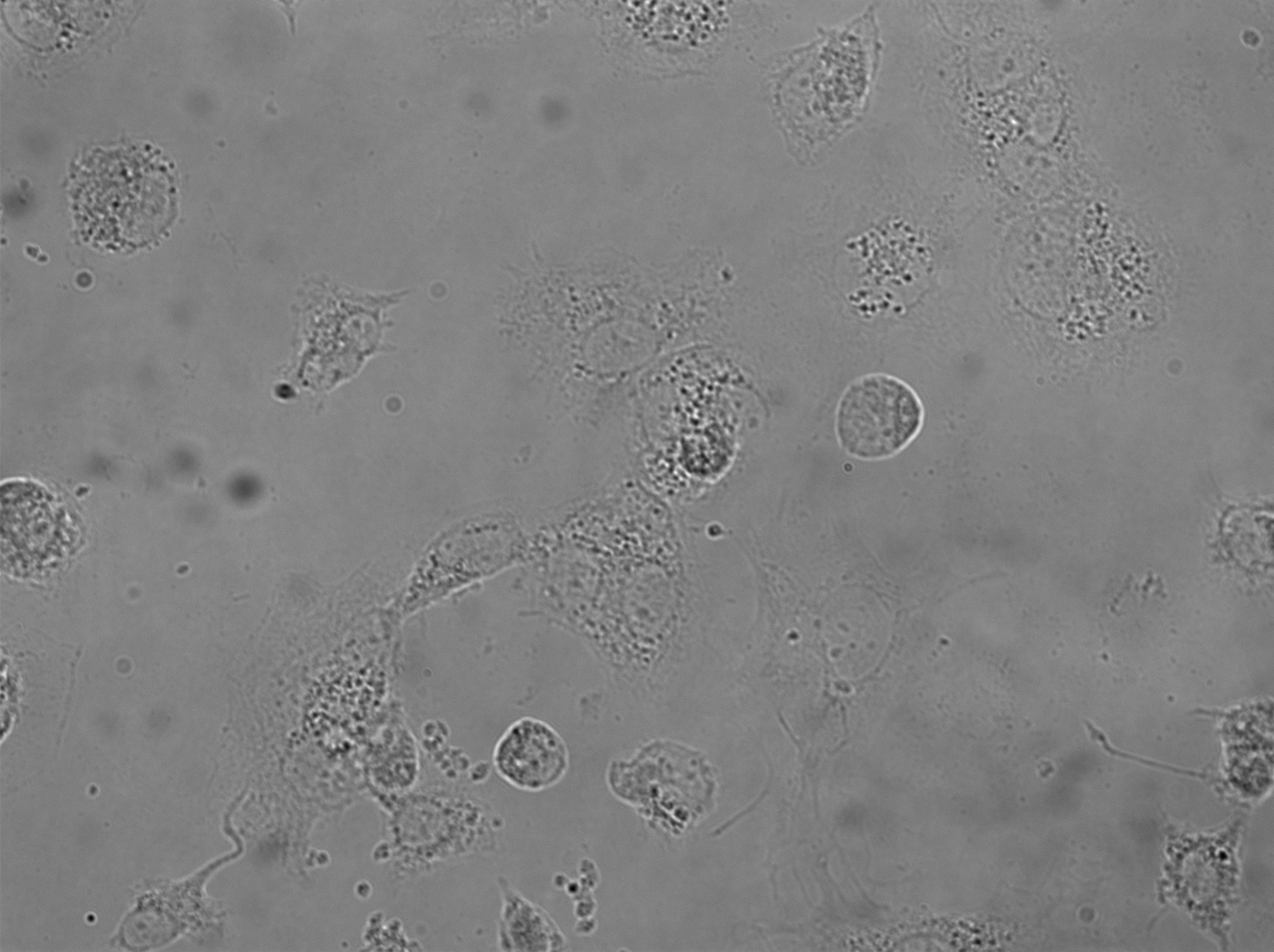

Supplement: File S2 — Transformed DAPI and Phase contrast (PC) image set of THP-1 macrophages infected by L. infantum parasites, and treated with increasing concentrations of glucantime (0-25-50-100 µg/ml). (ZIP) [file pntd.0002850.s004.zip › SI3/100/L30-PC.tif]

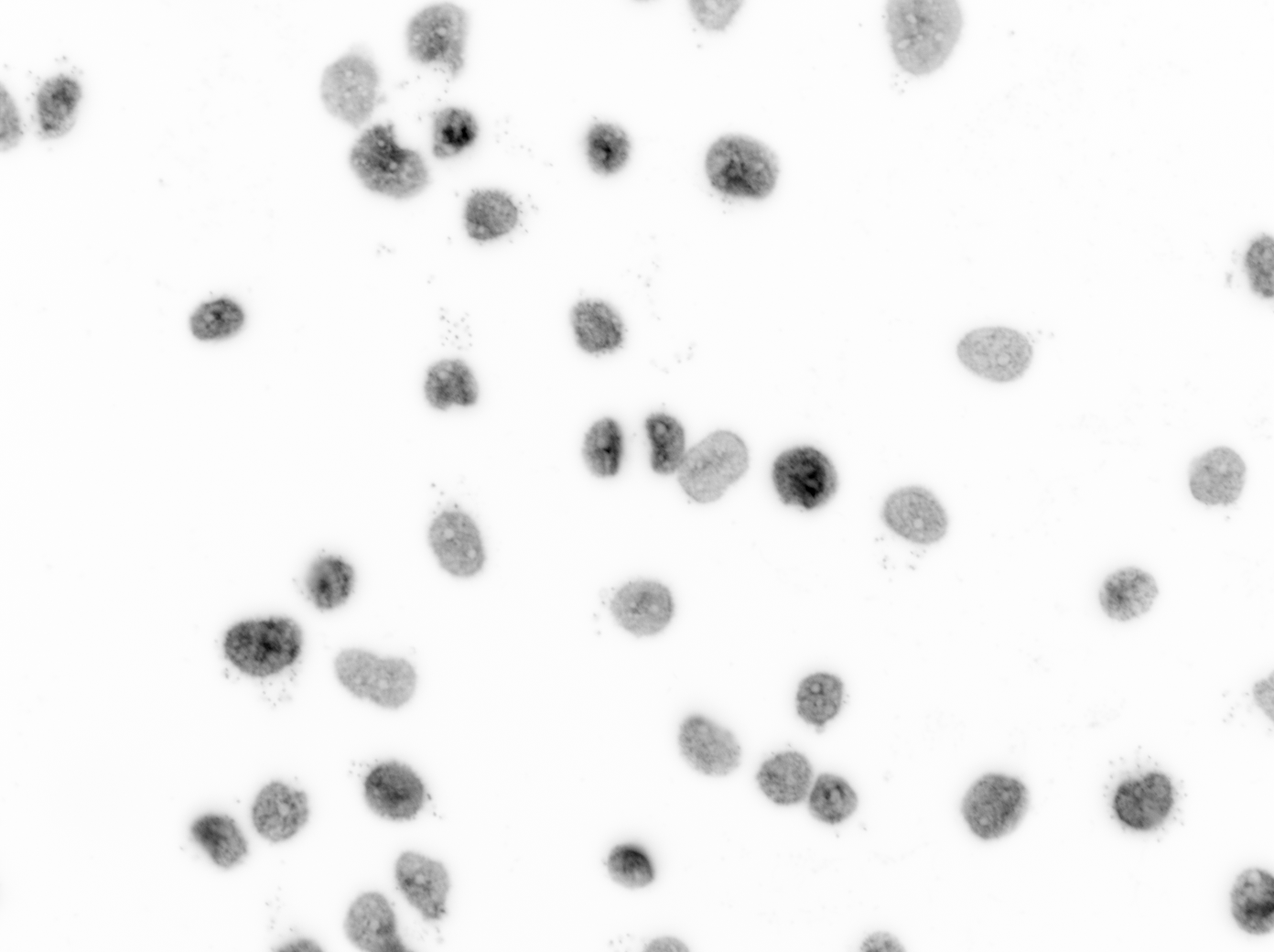

Supplement: File S2 — Transformed DAPI and Phase contrast (PC) image set of THP-1 macrophages infected by L. infantum parasites, and treated with increasing concentrations of glucantime (0-25-50-100 µg/ml). (ZIP) [file pntd.0002850.s004.zip › SI3/100/L31-DAPI.tif]

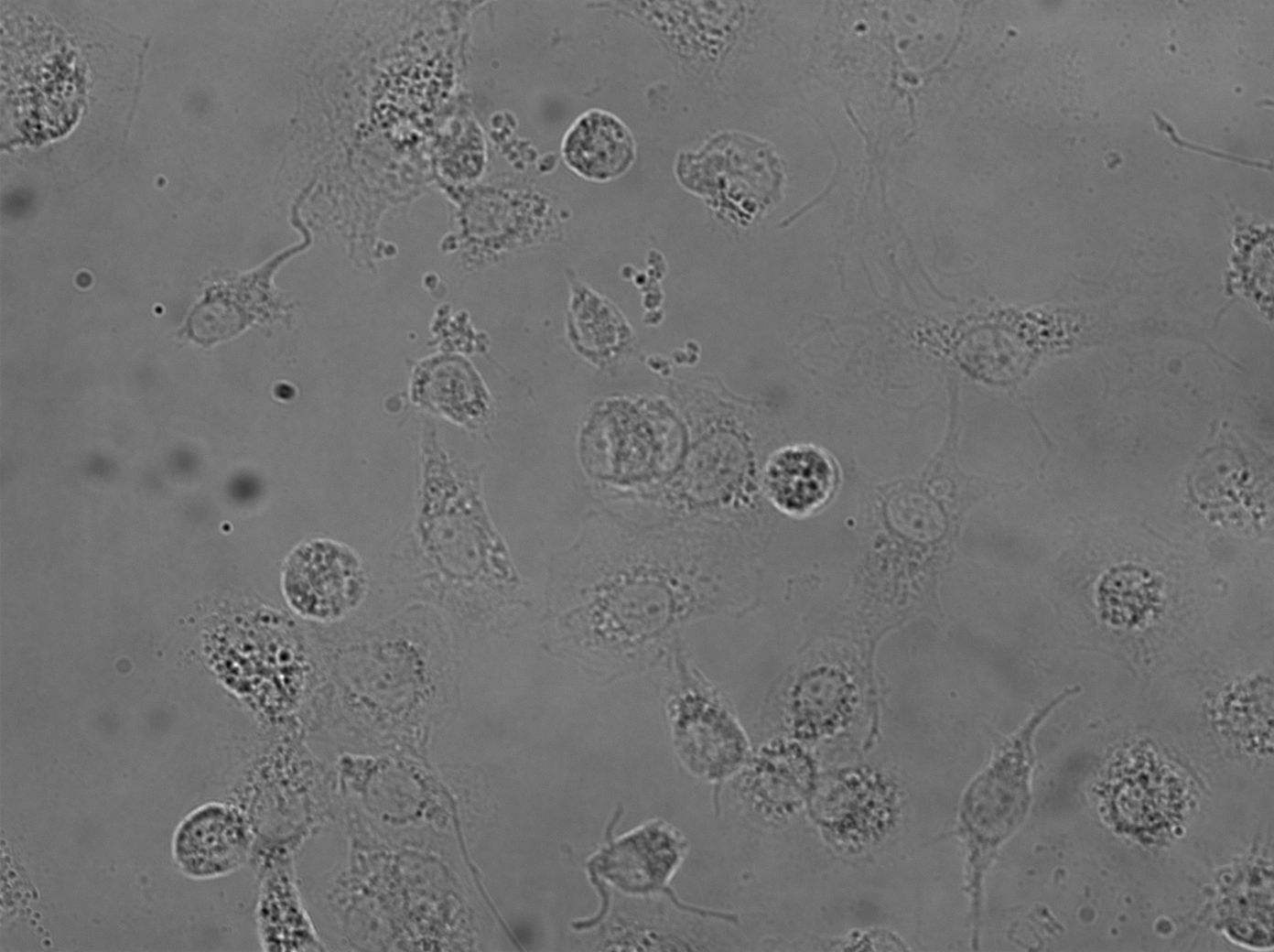

Supplement: File S2 — Transformed DAPI and Phase contrast (PC) image set of THP-1 macrophages infected by L. infantum parasites, and treated with increasing concentrations of glucantime (0-25-50-100 µg/ml). (ZIP) [file pntd.0002850.s004.zip › SI3/100/L31-PC.tif]

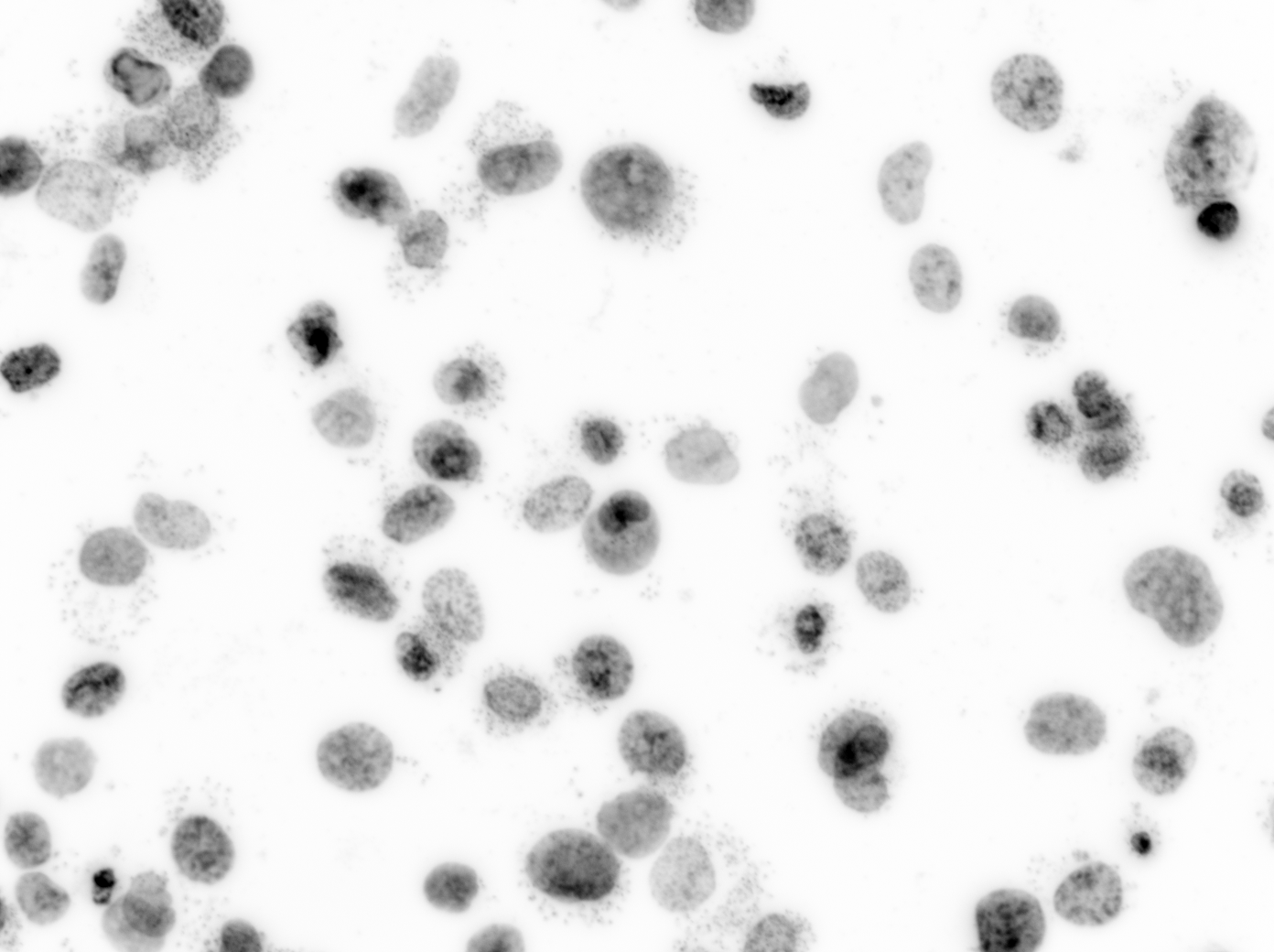

Supplement: File S2 — Transformed DAPI and Phase contrast (PC) image set of THP-1 macrophages infected by L. infantum parasites, and treated with increasing concentrations of glucantime (0-25-50-100 µg/ml). (ZIP) [file pntd.0002850.s004.zip › SI3/25/L10-DAPI.tif]

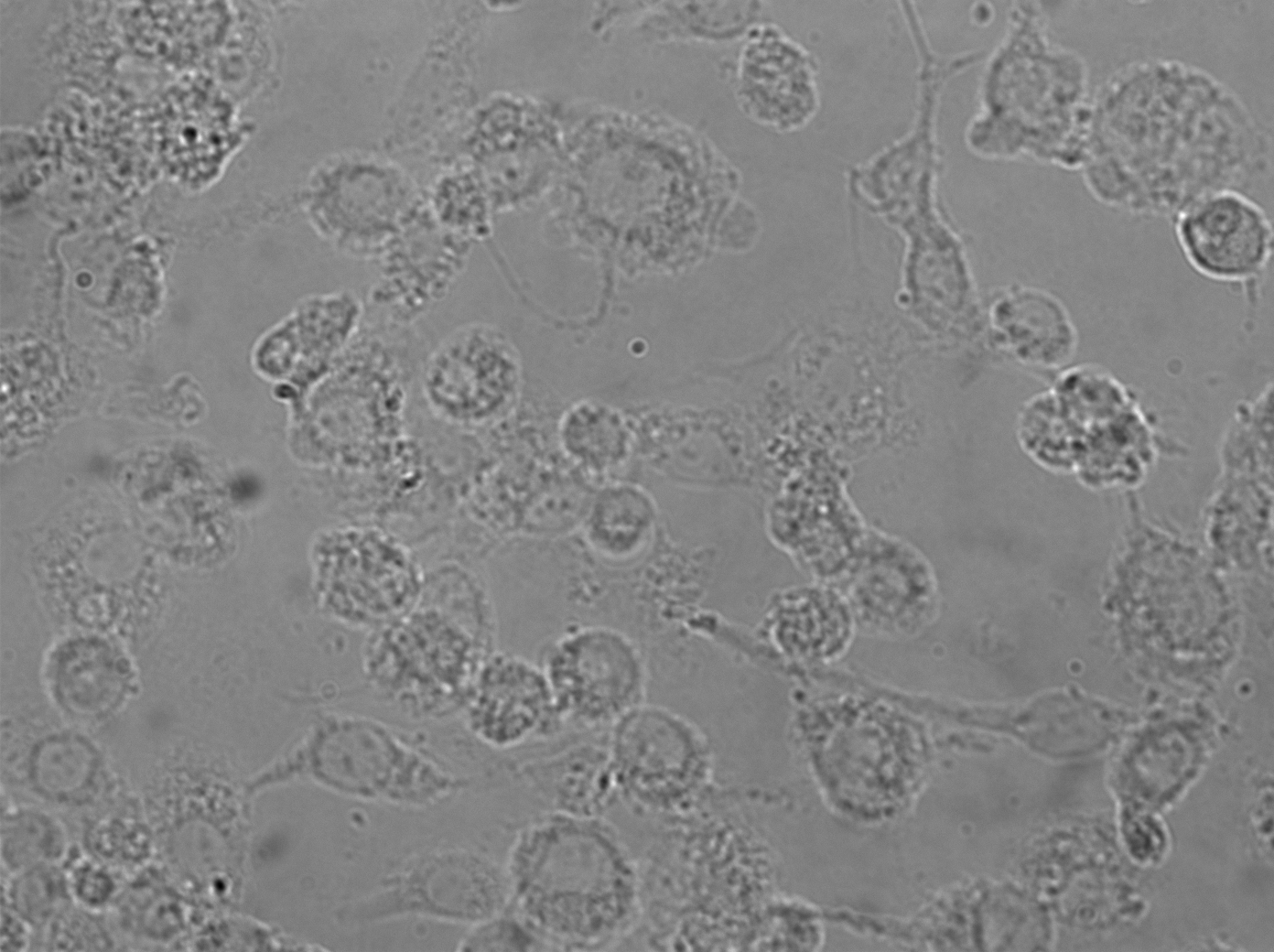

Supplement: File S2 — Transformed DAPI and Phase contrast (PC) image set of THP-1 macrophages infected by L. infantum parasites, and treated with increasing concentrations of glucantime (0-25-50-100 µg/ml). (ZIP) [file pntd.0002850.s004.zip › SI3/25/L10-PC.tif]

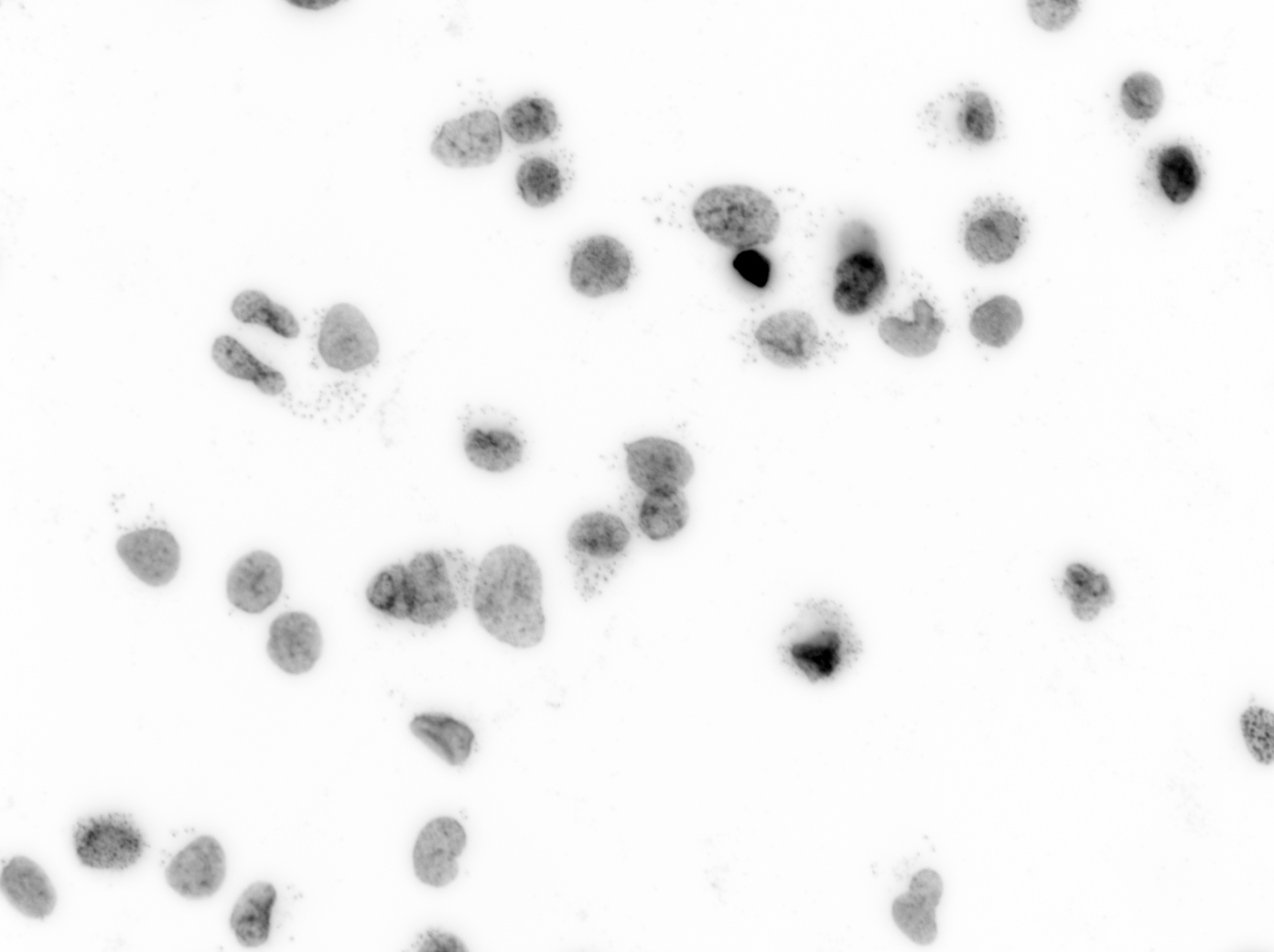

Supplement: File S2 — Transformed DAPI and Phase contrast (PC) image set of THP-1 macrophages infected by L. infantum parasites, and treated with increasing concentrations of glucantime (0-25-50-100 µg/ml). (ZIP) [file pntd.0002850.s004.zip › SI3/25/L11-DAPI.tif]

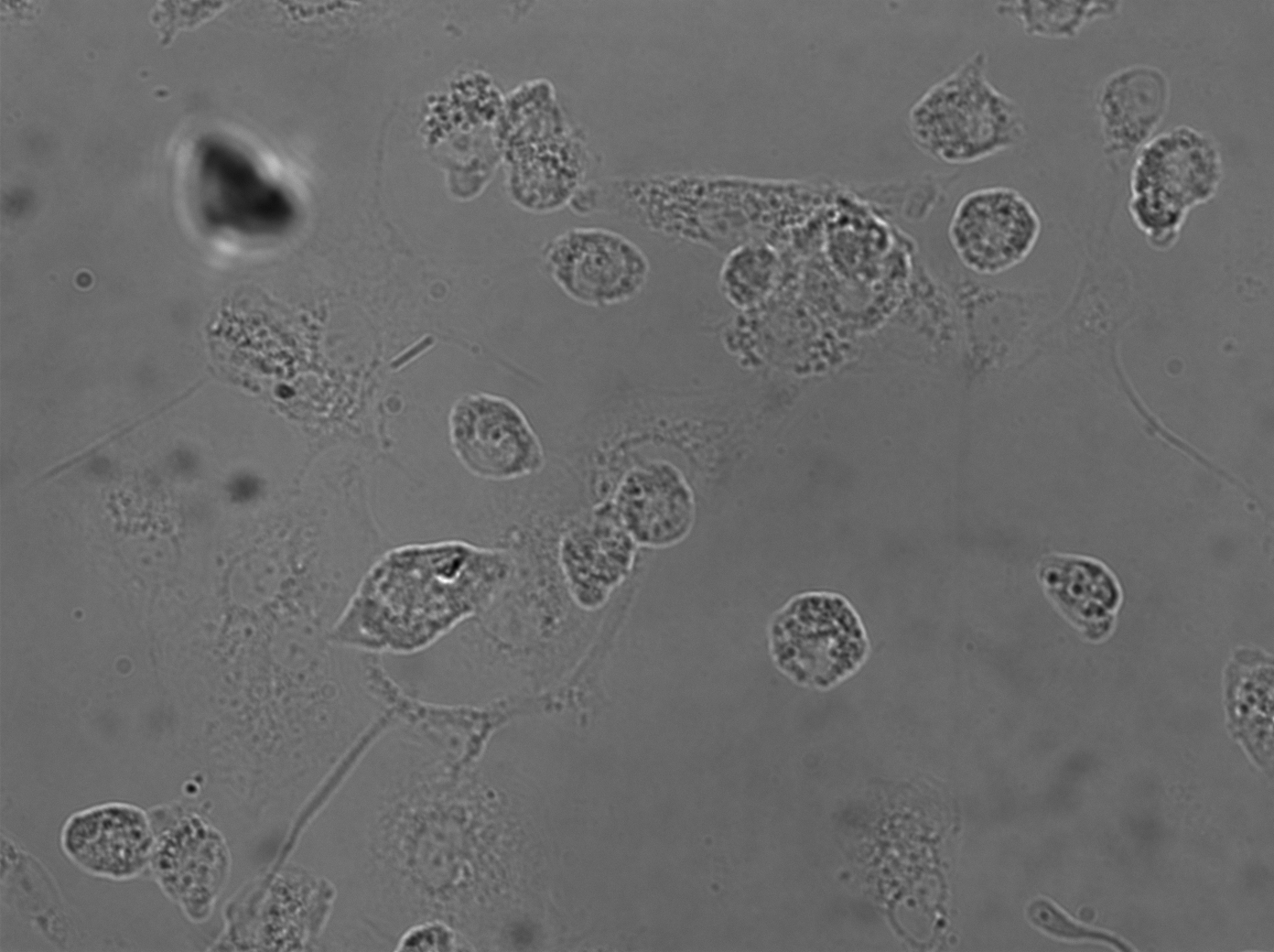

Supplement: File S2 — Transformed DAPI and Phase contrast (PC) image set of THP-1 macrophages infected by L. infantum parasites, and treated with increasing concentrations of glucantime (0-25-50-100 µg/ml). (ZIP) [file pntd.0002850.s004.zip › SI3/25/L11-PC.tif]

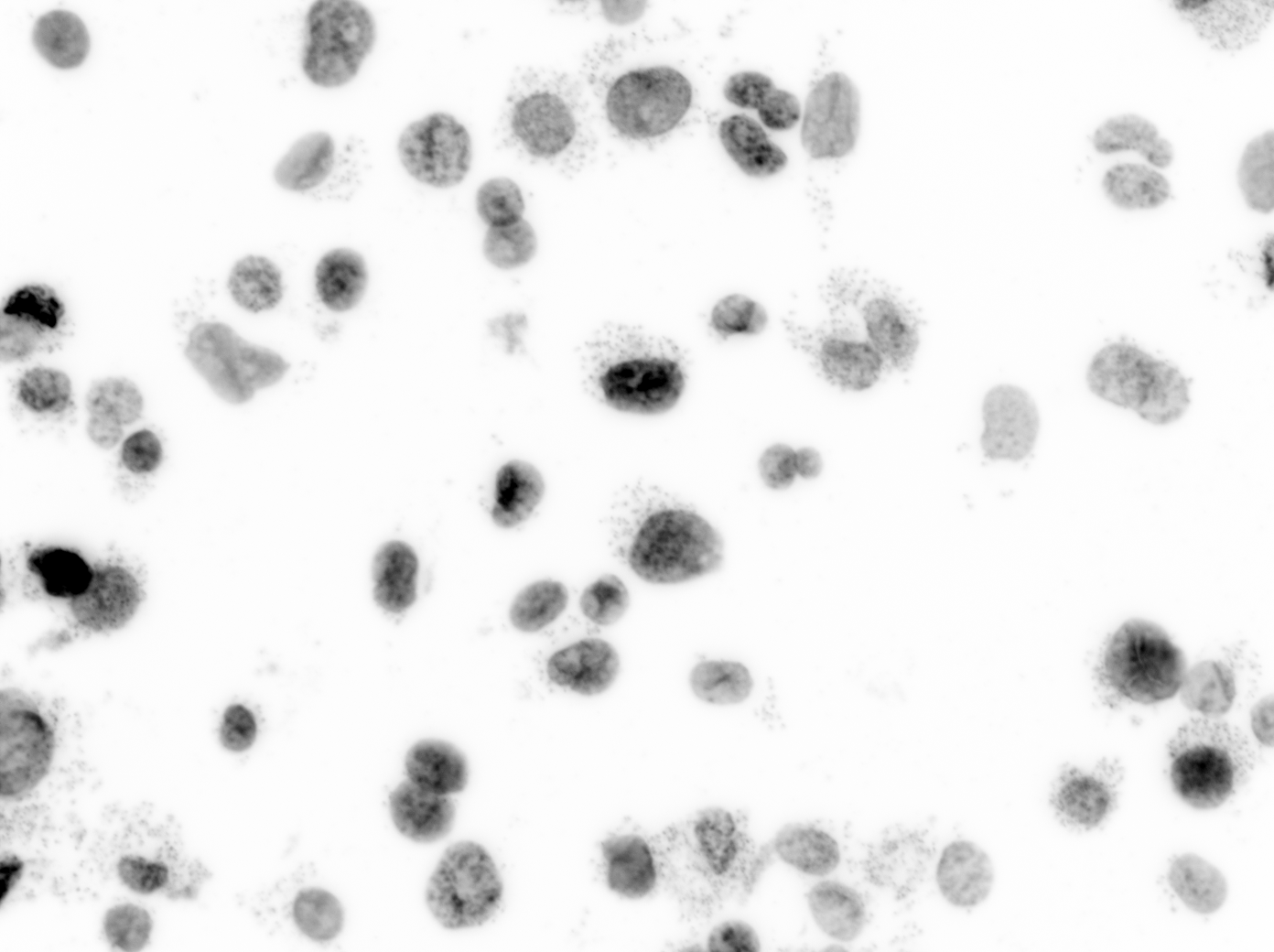

Supplement: File S2 — Transformed DAPI and Phase contrast (PC) image set of THP-1 macrophages infected by L. infantum parasites, and treated with increasing concentrations of glucantime (0-25-50-100 µg/ml). (ZIP) [file pntd.0002850.s004.zip › SI3/25/L12-DAPI.tif]

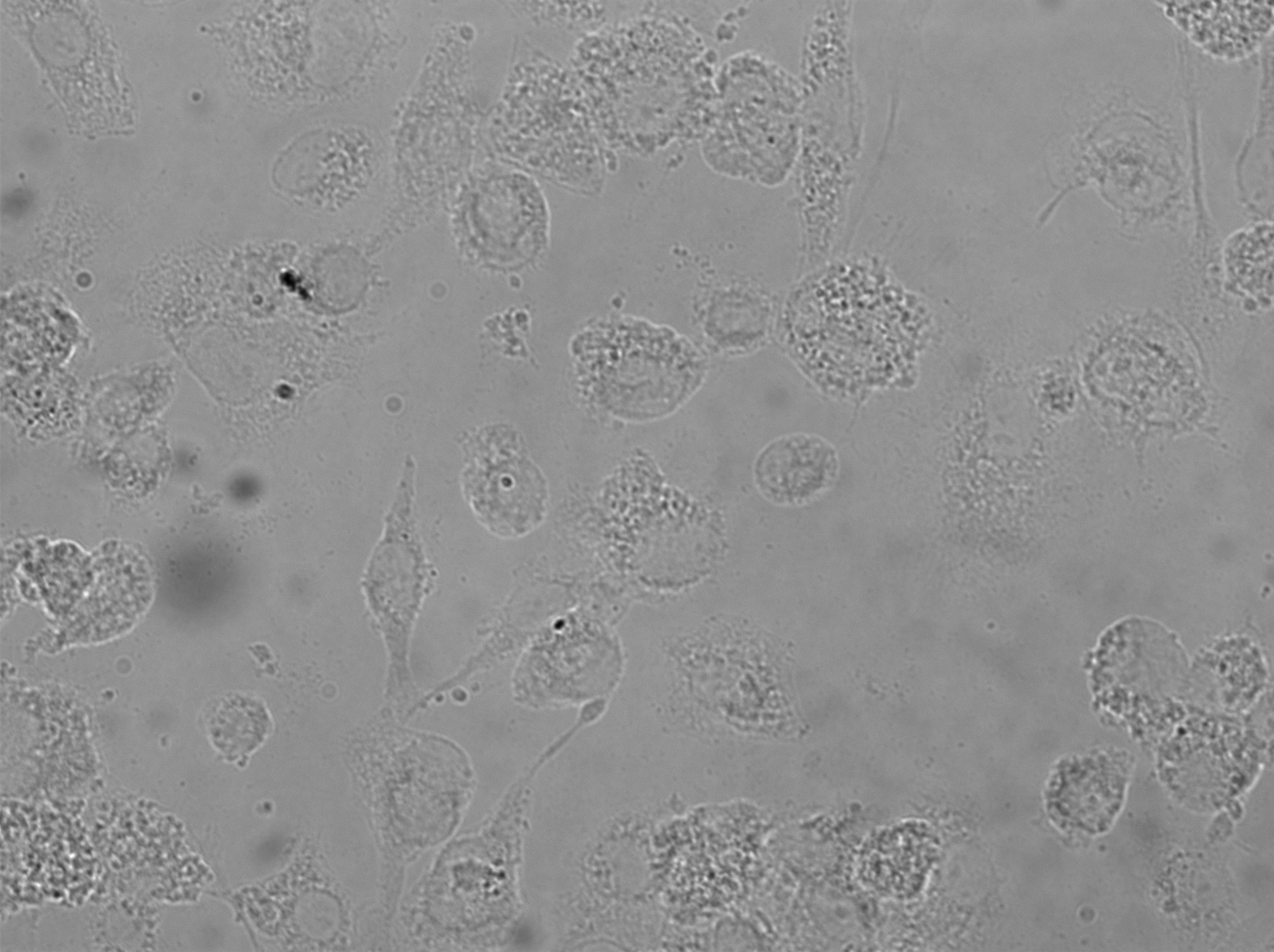

Supplement: File S2 — Transformed DAPI and Phase contrast (PC) image set of THP-1 macrophages infected by L. infantum parasites, and treated with increasing concentrations of glucantime (0-25-50-100 µg/ml). (ZIP) [file pntd.0002850.s004.zip › SI3/25/L12-PC.tif]

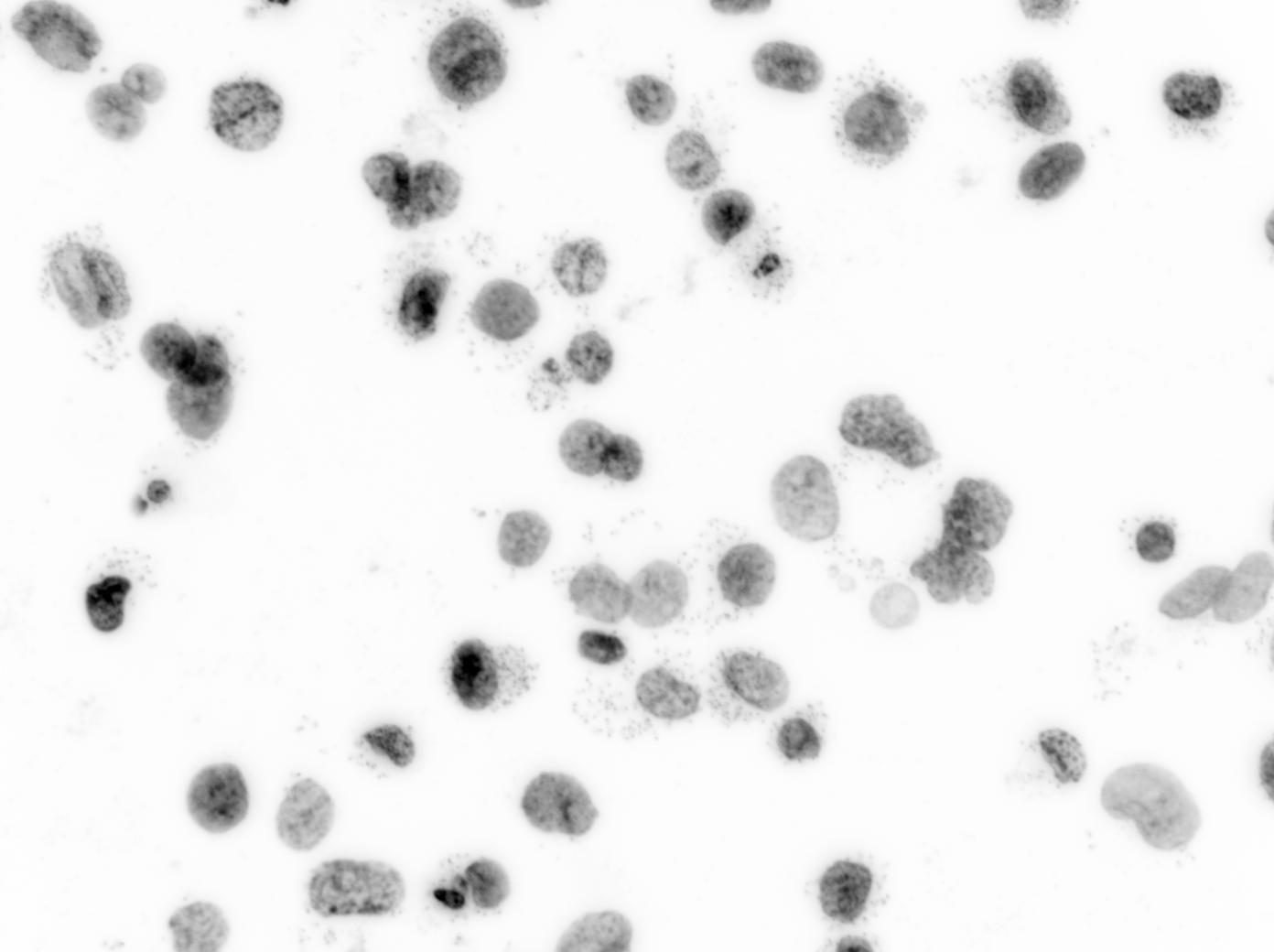

Supplement: File S2 — Transformed DAPI and Phase contrast (PC) image set of THP-1 macrophages infected by L. infantum parasites, and treated with increasing concentrations of glucantime (0-25-50-100 µg/ml). (ZIP) [file pntd.0002850.s004.zip › SI3/25/L13-DAPI.tif]

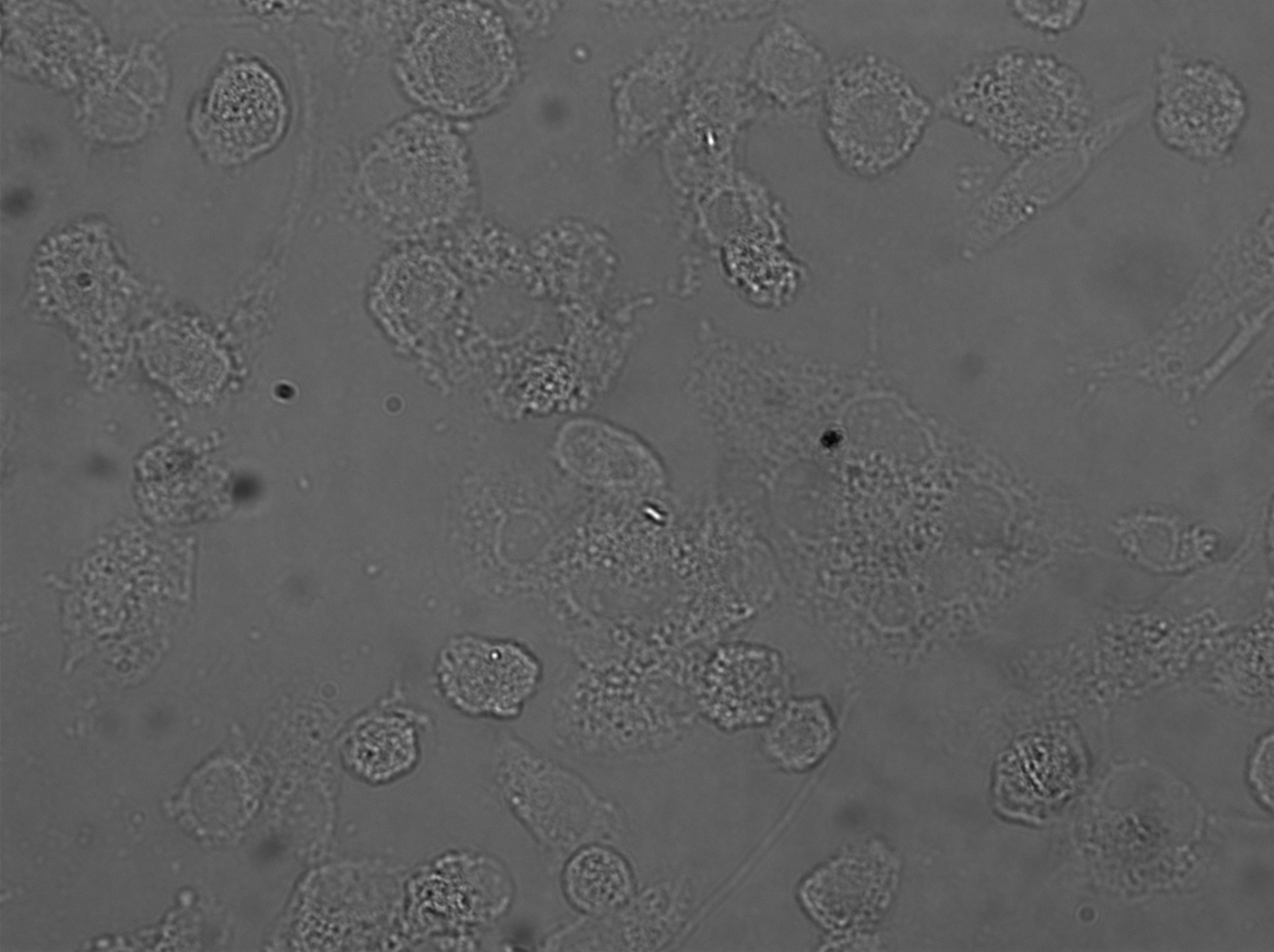

Supplement: File S2 — Transformed DAPI and Phase contrast (PC) image set of THP-1 macrophages infected by L. infantum parasites, and treated with increasing concentrations of glucantime (0-25-50-100 µg/ml). (ZIP) [file pntd.0002850.s004.zip › SI3/25/L13-PC.tif]

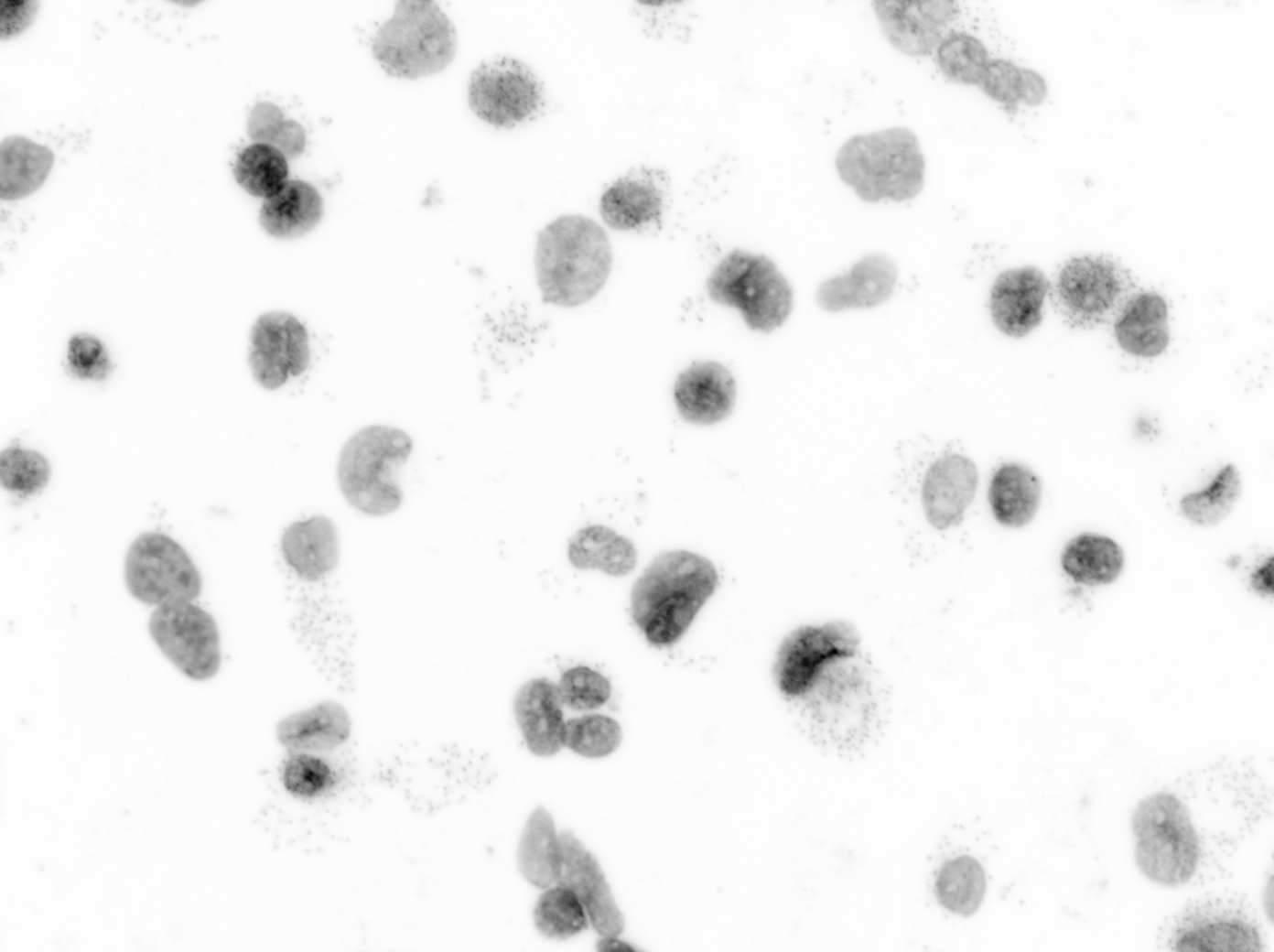

Supplement: File S2 — Transformed DAPI and Phase contrast (PC) image set of THP-1 macrophages infected by L. infantum parasites, and treated with increasing concentrations of glucantime (0-25-50-100 µg/ml). (ZIP) [file pntd.0002850.s004.zip › SI3/25/L14-DAPI.tif]

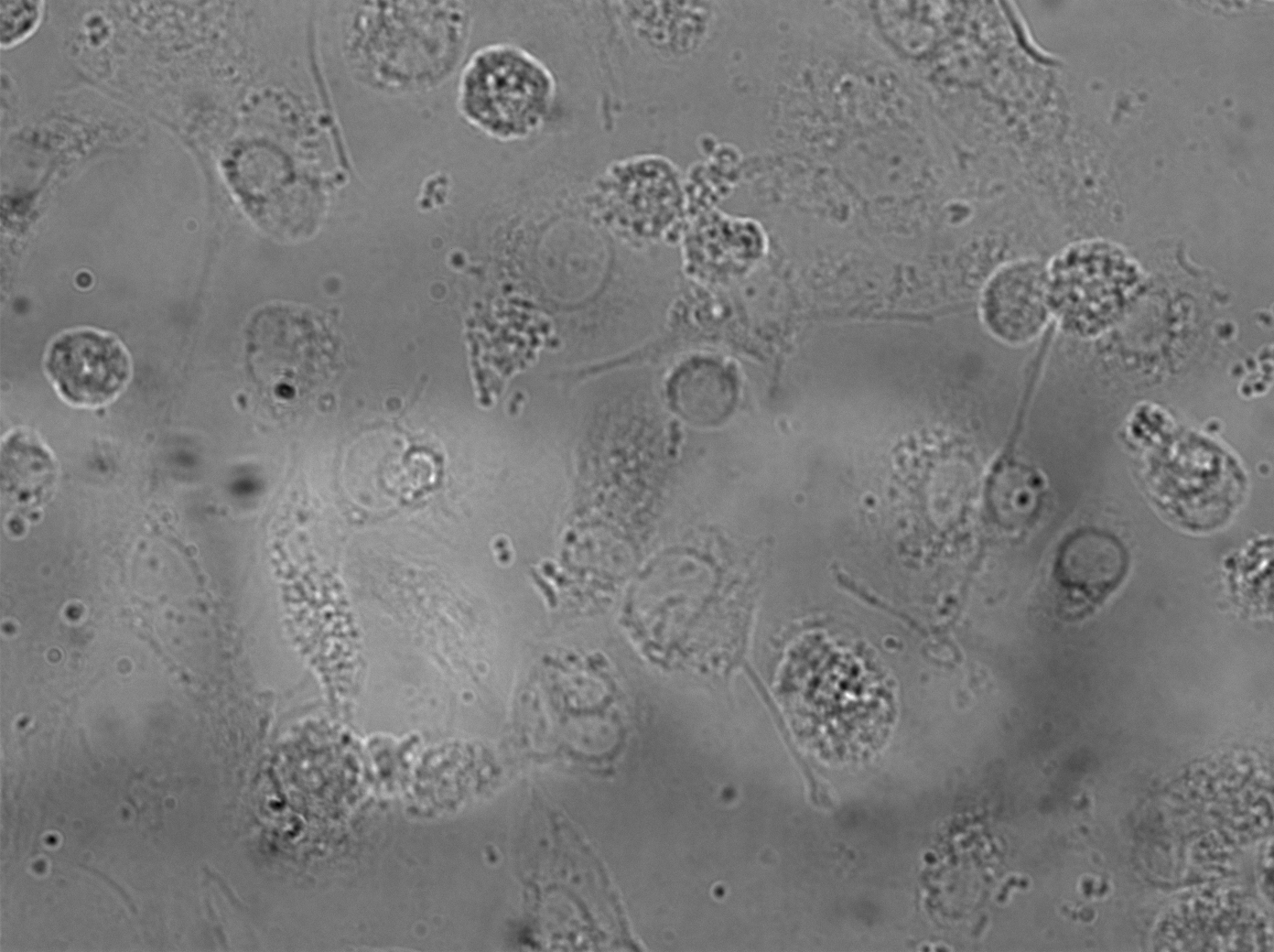

Supplement: File S2 — Transformed DAPI and Phase contrast (PC) image set of THP-1 macrophages infected by L. infantum parasites, and treated with increasing concentrations of glucantime (0-25-50-100 µg/ml). (ZIP) [file pntd.0002850.s004.zip › SI3/25/L14-PC.tif]

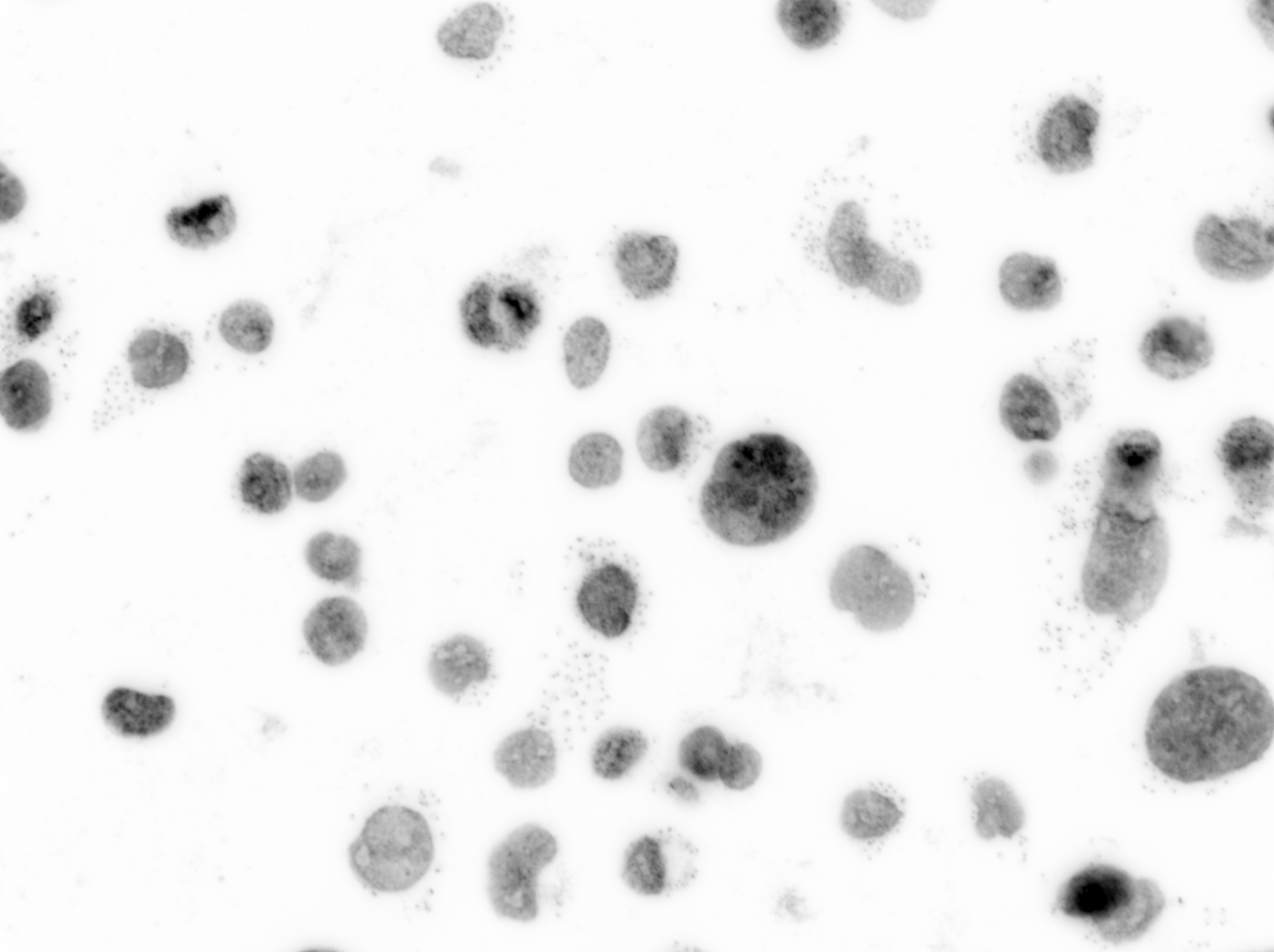

Supplement: File S2 — Transformed DAPI and Phase contrast (PC) image set of THP-1 macrophages infected by L. infantum parasites, and treated with increasing concentrations of glucantime (0-25-50-100 µg/ml). (ZIP) [file pntd.0002850.s004.zip › SI3/25/L15-DAPI.tif]

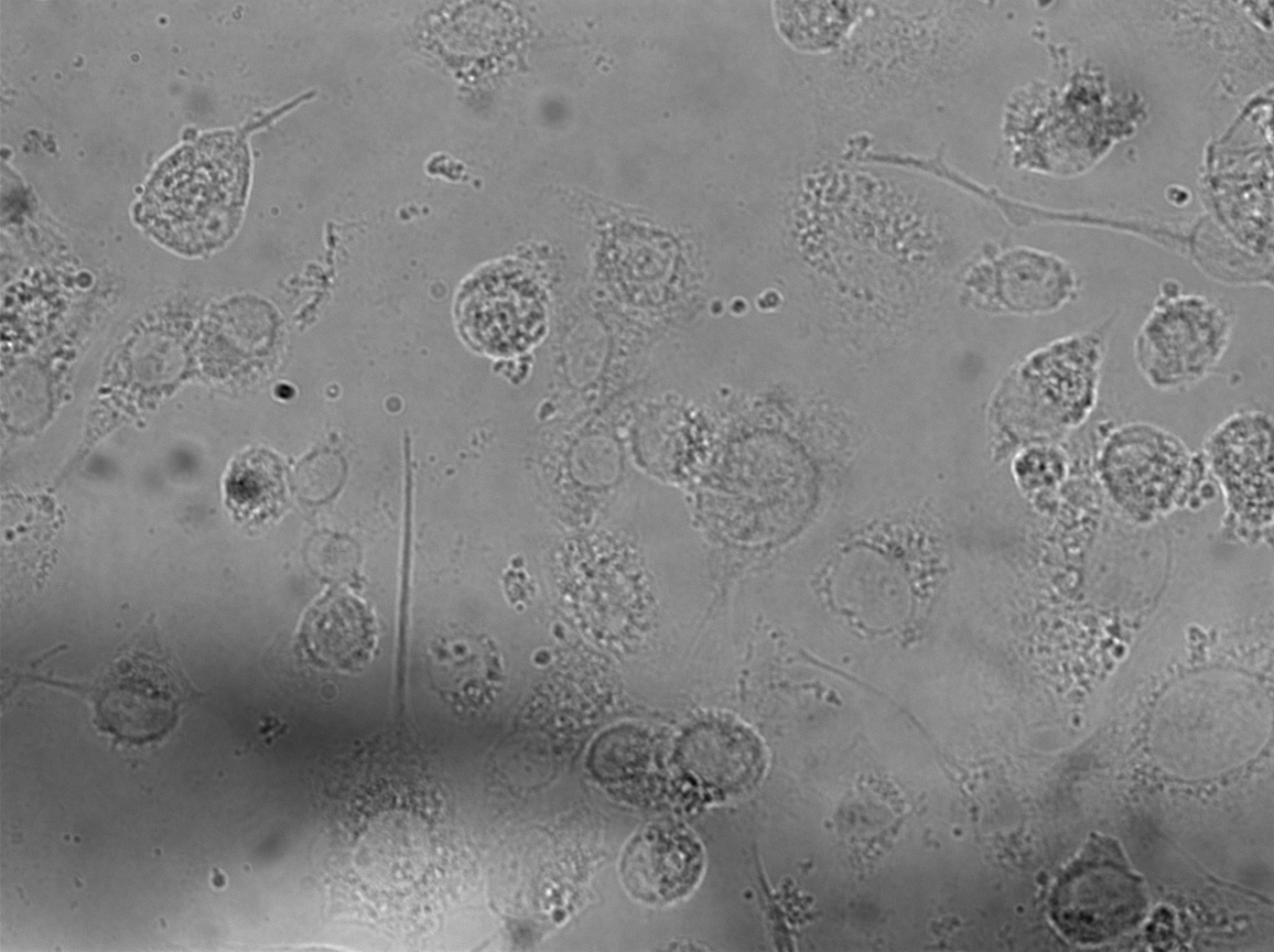

Supplement: File S2 — Transformed DAPI and Phase contrast (PC) image set of THP-1 macrophages infected by L. infantum parasites, and treated with increasing concentrations of glucantime (0-25-50-100 µg/ml). (ZIP) [file pntd.0002850.s004.zip › SI3/25/L15-PC.tif]

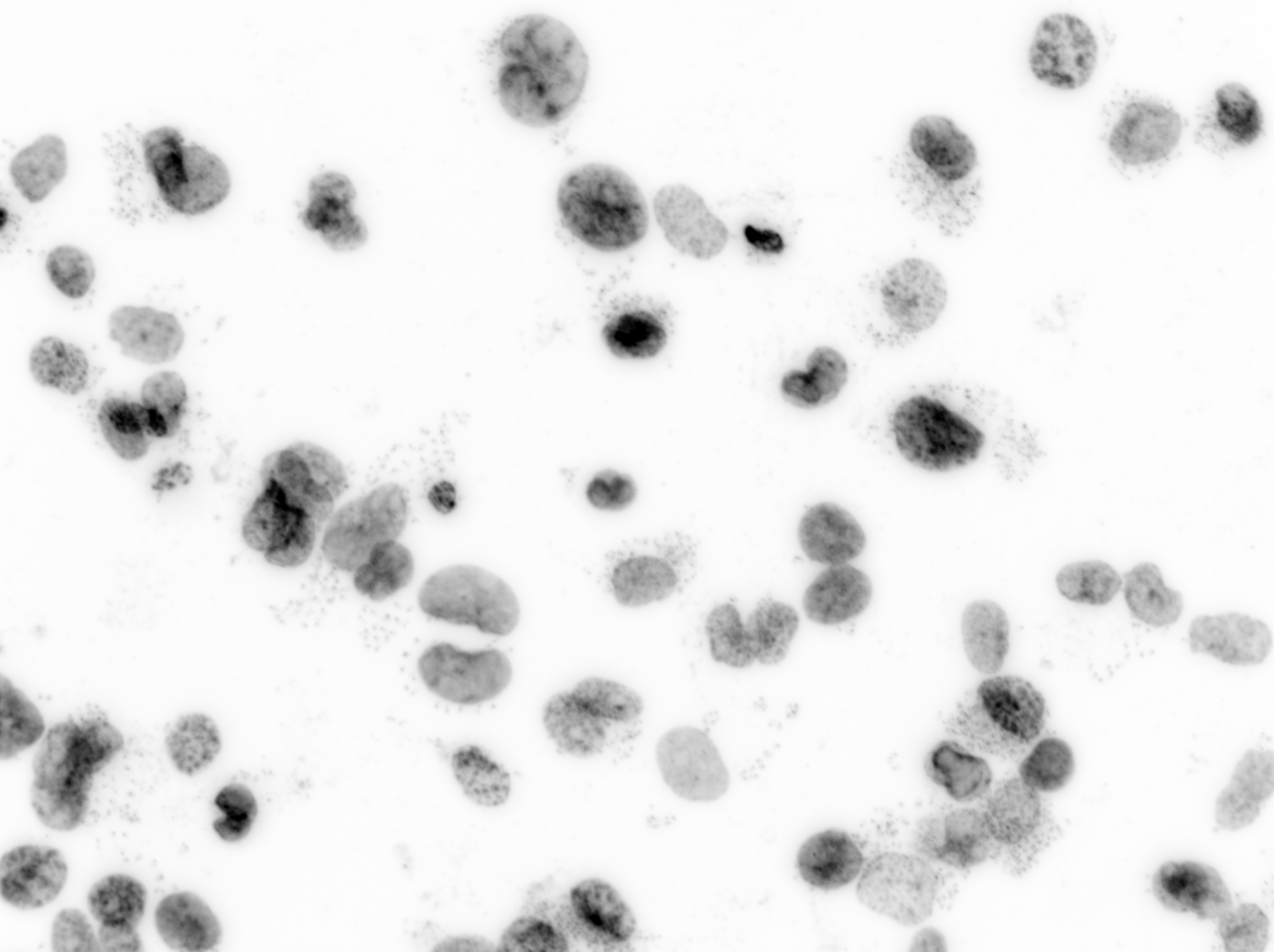

Supplement: File S2 — Transformed DAPI and Phase contrast (PC) image set of THP-1 macrophages infected by L. infantum parasites, and treated with increasing concentrations of glucantime (0-25-50-100 µg/ml). (ZIP) [file pntd.0002850.s004.zip › SI3/25/L8-DAPI.tif]

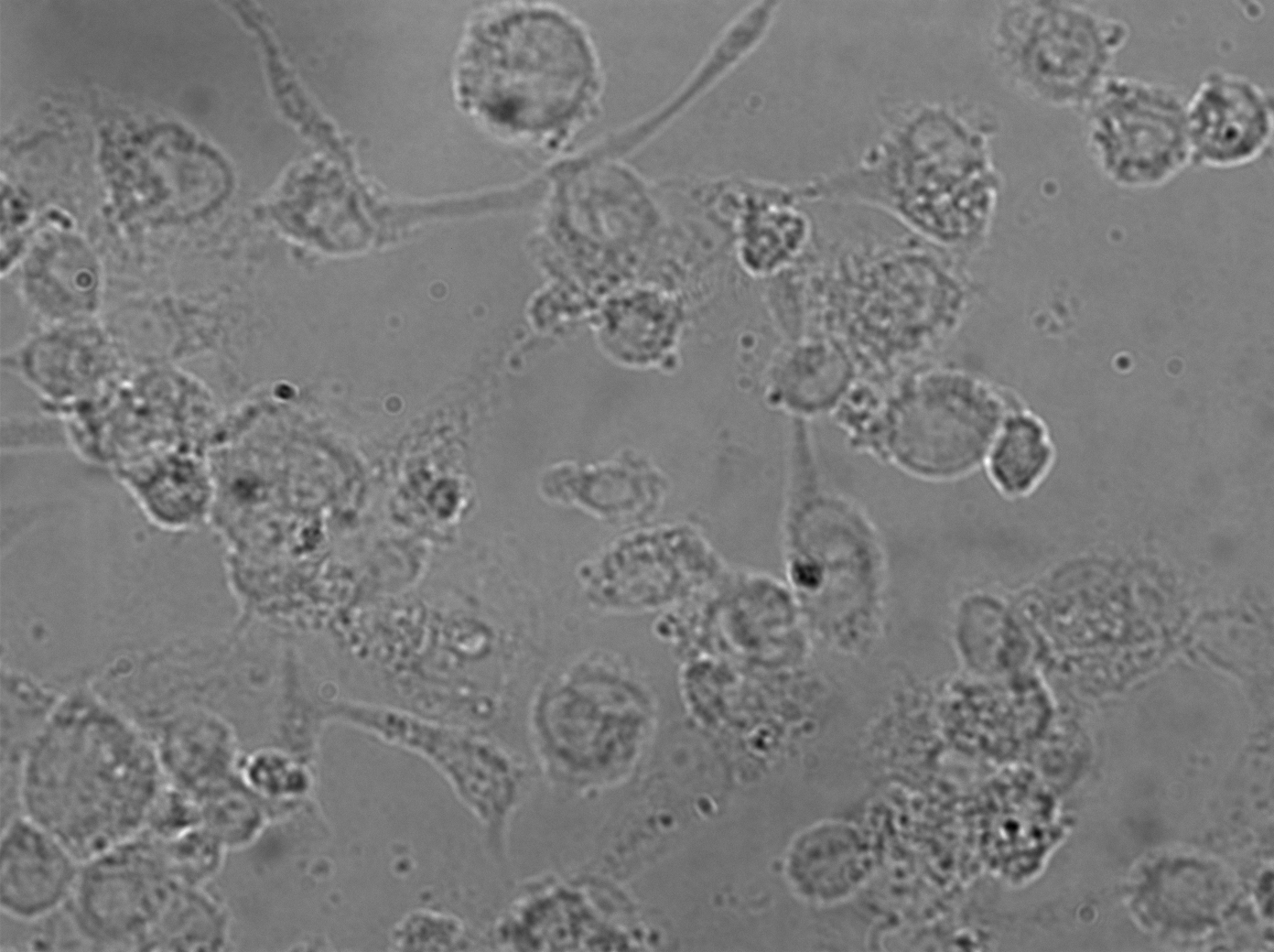

Supplement: File S2 — Transformed DAPI and Phase contrast (PC) image set of THP-1 macrophages infected by L. infantum parasites, and treated with increasing concentrations of glucantime (0-25-50-100 µg/ml). (ZIP) [file pntd.0002850.s004.zip › SI3/25/L8-PC.tif]

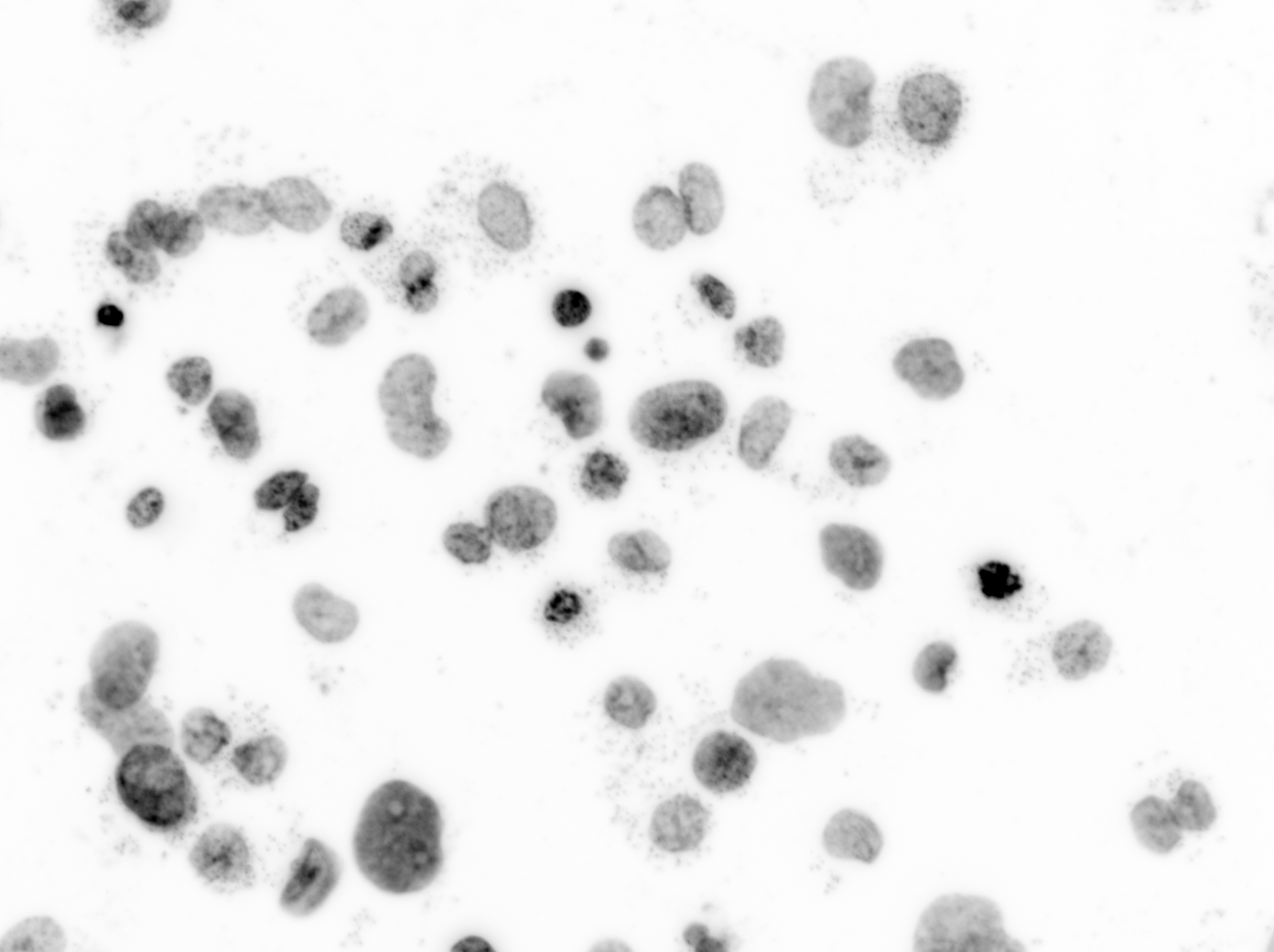

Supplement: File S2 — Transformed DAPI and Phase contrast (PC) image set of THP-1 macrophages infected by L. infantum parasites, and treated with increasing concentrations of glucantime (0-25-50-100 µg/ml). (ZIP) [file pntd.0002850.s004.zip › SI3/25/L9-DAPI.tif]

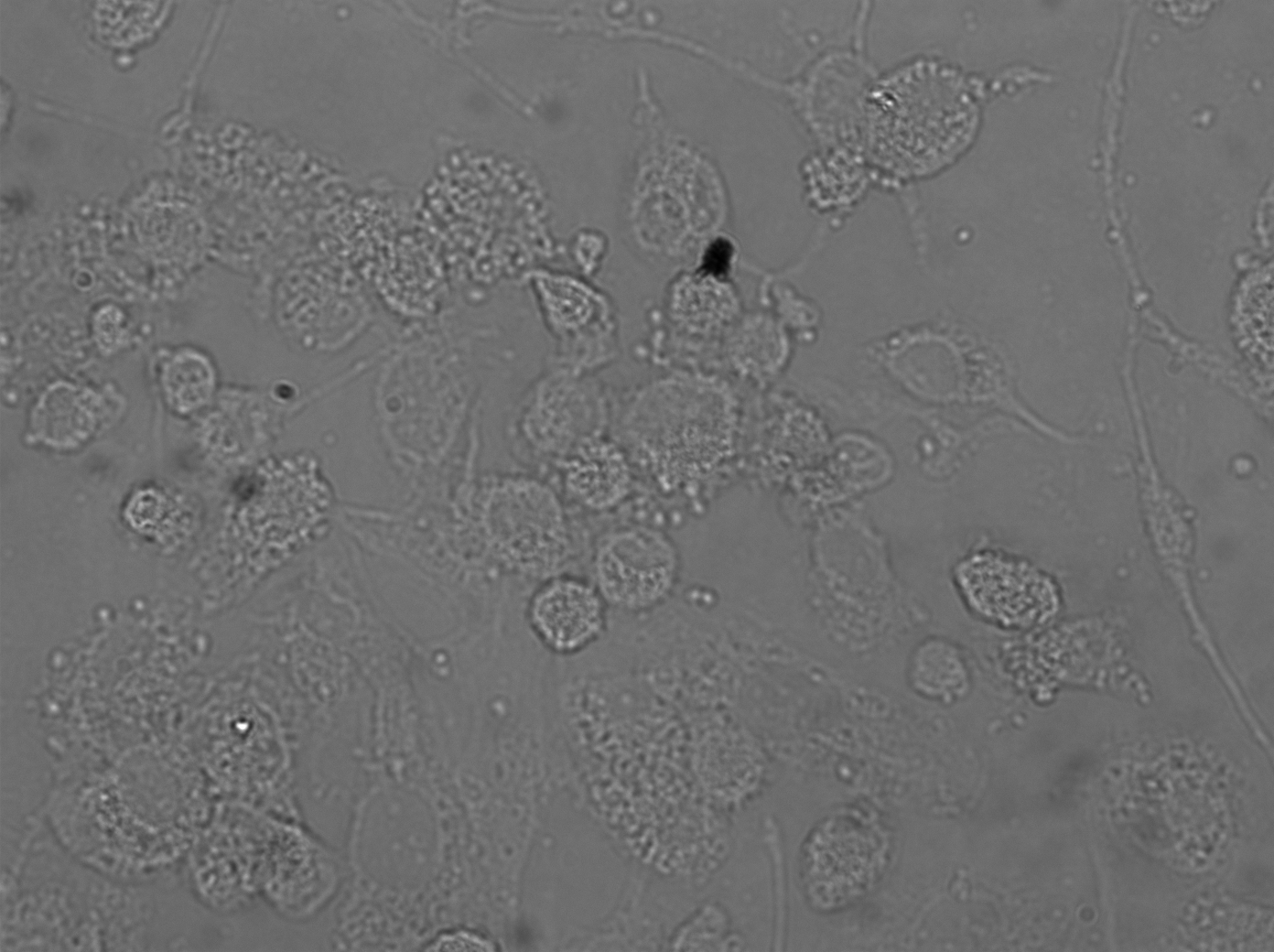

Supplement: File S2 — Transformed DAPI and Phase contrast (PC) image set of THP-1 macrophages infected by L. infantum parasites, and treated with increasing concentrations of glucantime (0-25-50-100 µg/ml). (ZIP) [file pntd.0002850.s004.zip › SI3/25/L9-PC.tif]

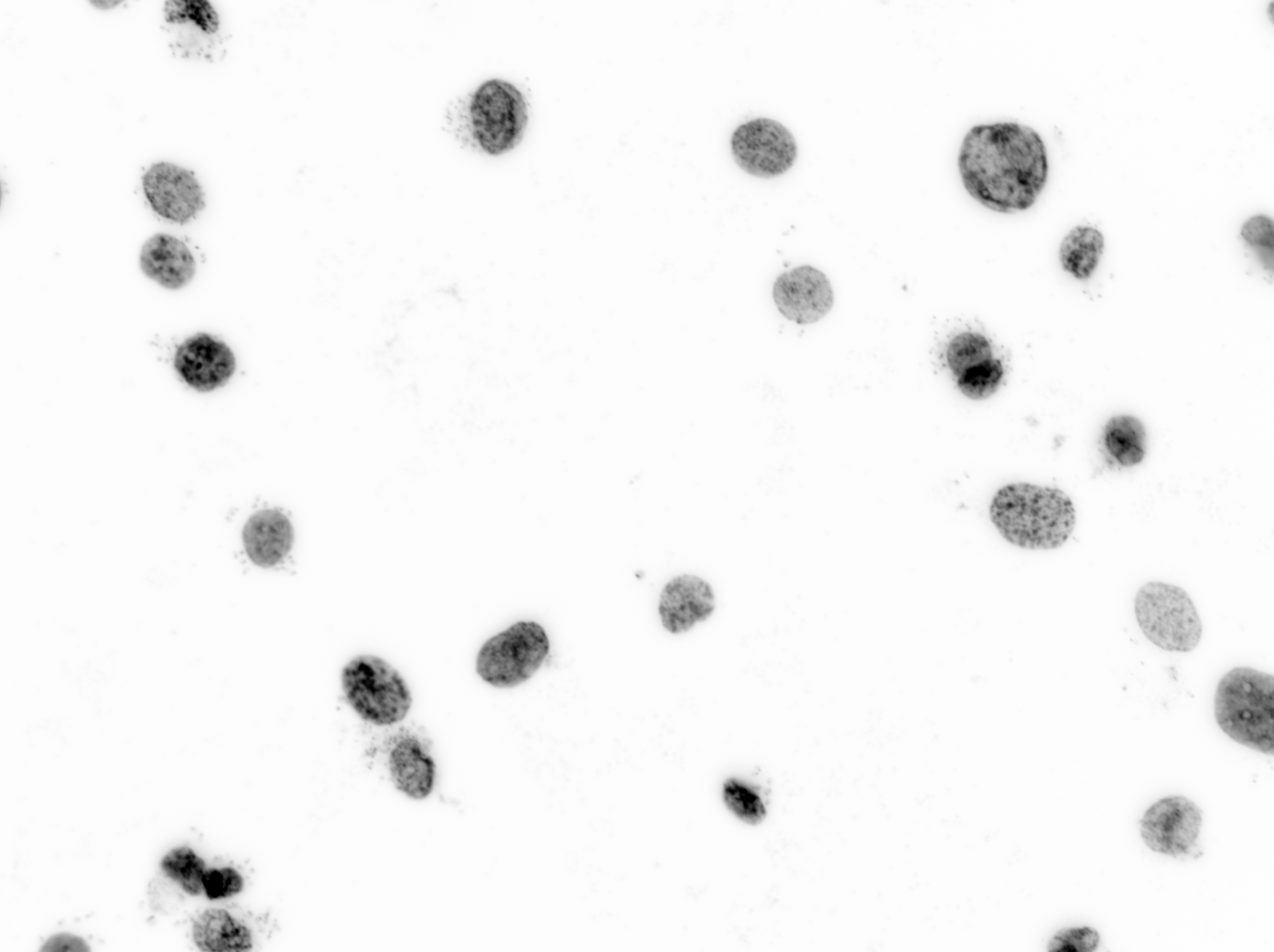

Supplement: File S2 — Transformed DAPI and Phase contrast (PC) image set of THP-1 macrophages infected by L. infantum parasites, and treated with increasing concentrations of glucantime (0-25-50-100 µg/ml). (ZIP) [file pntd.0002850.s004.zip › SI3/50/L16-DAPI.tif]

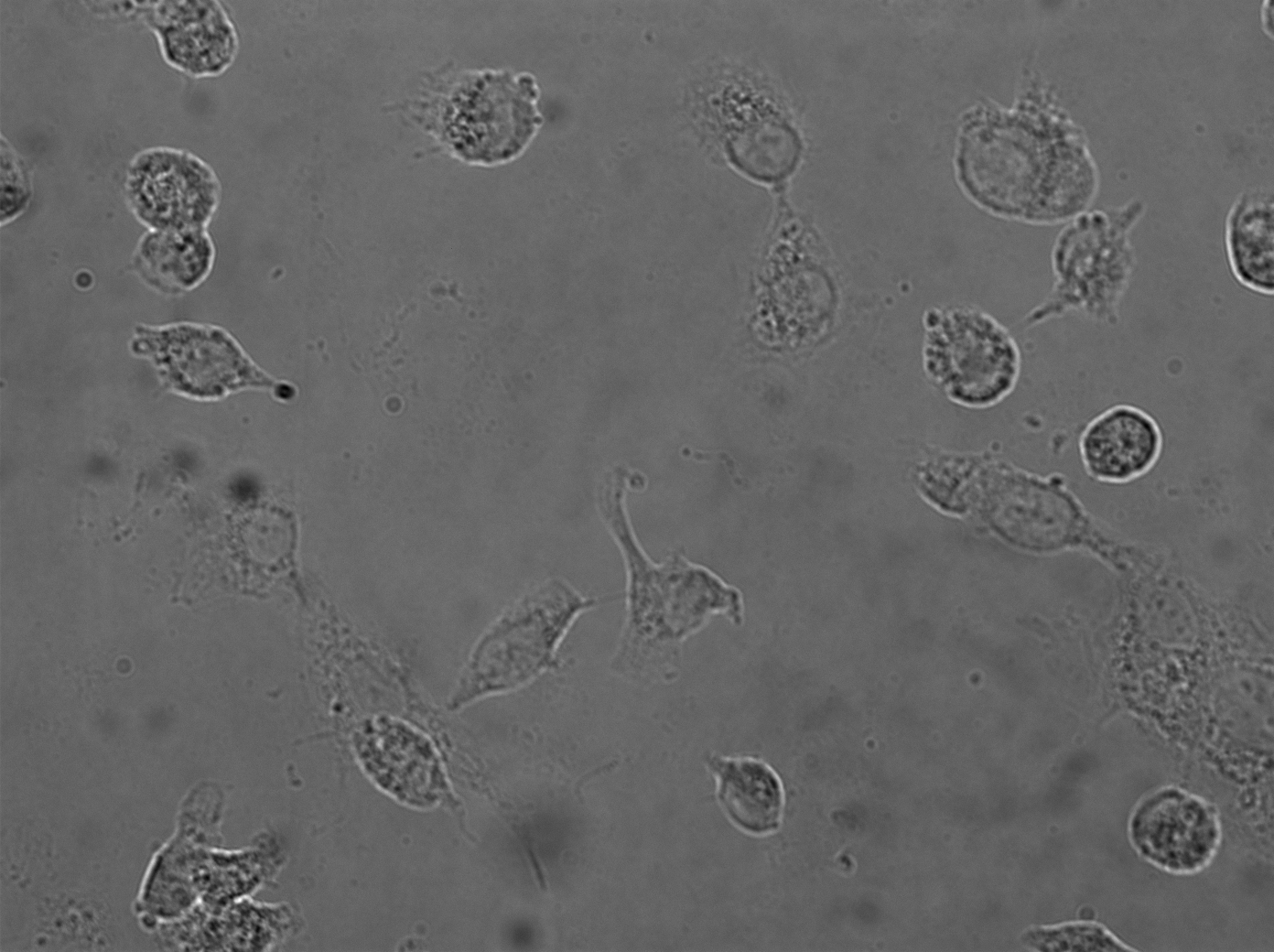

Supplement: File S2 — Transformed DAPI and Phase contrast (PC) image set of THP-1 macrophages infected by L. infantum parasites, and treated with increasing concentrations of glucantime (0-25-50-100 µg/ml). (ZIP) [file pntd.0002850.s004.zip › SI3/50/L16-PC.tif]

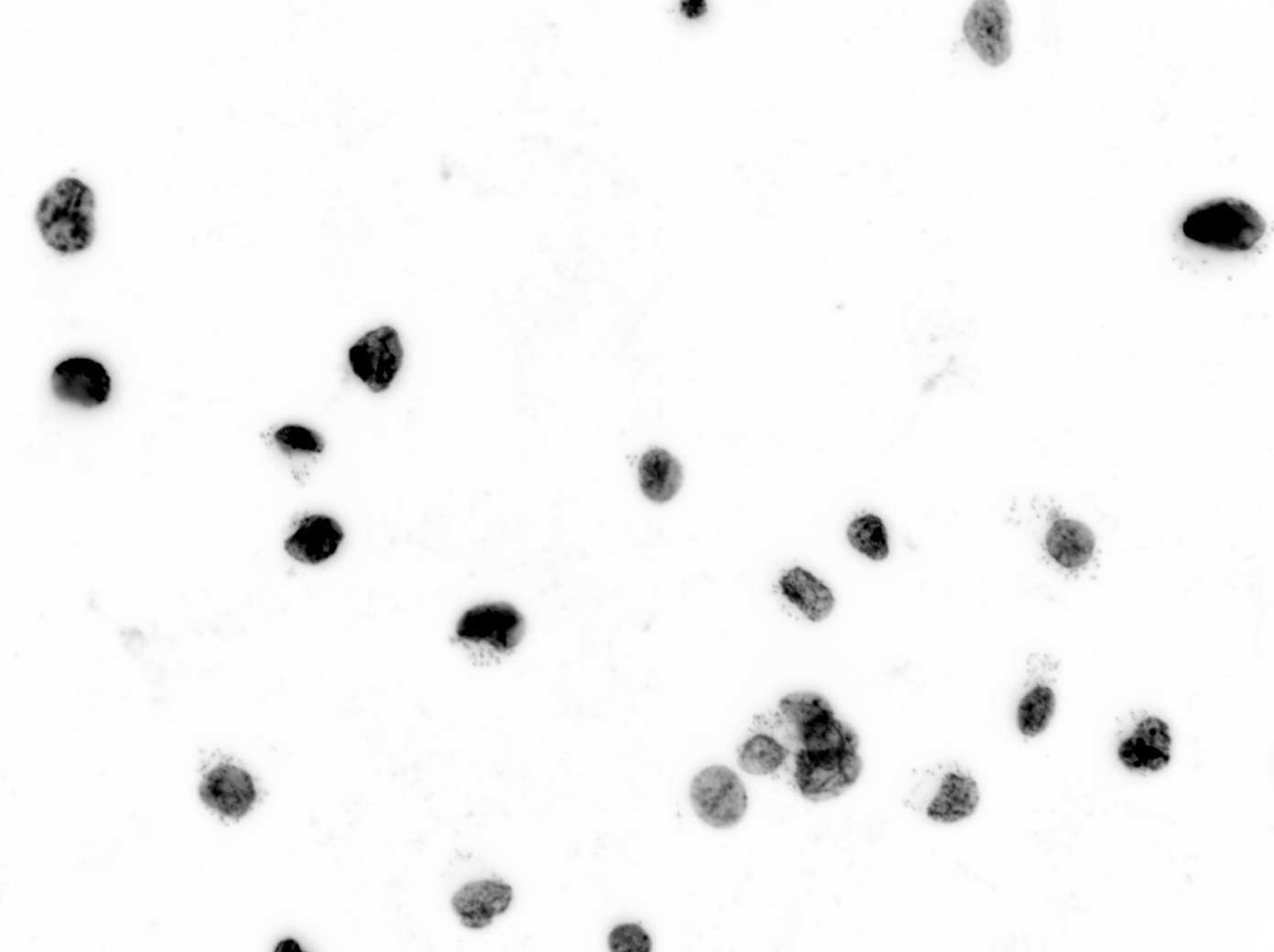

Supplement: File S2 — Transformed DAPI and Phase contrast (PC) image set of THP-1 macrophages infected by L. infantum parasites, and treated with increasing concentrations of glucantime (0-25-50-100 µg/ml). (ZIP) [file pntd.0002850.s004.zip › SI3/50/L17-DAPI.tif]

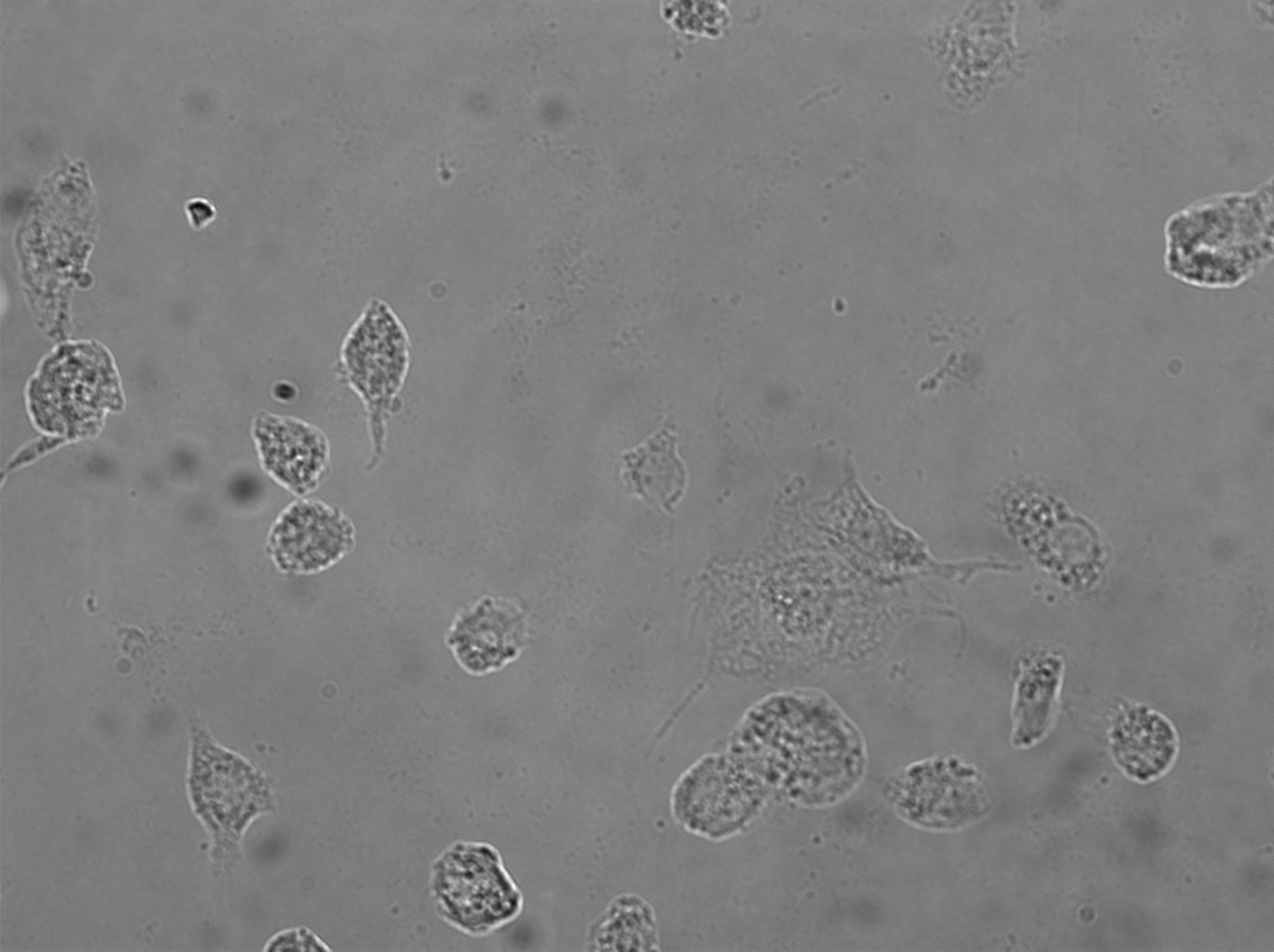

Supplement: File S2 — Transformed DAPI and Phase contrast (PC) image set of THP-1 macrophages infected by L. infantum parasites, and treated with increasing concentrations of glucantime (0-25-50-100 µg/ml). (ZIP) [file pntd.0002850.s004.zip › SI3/50/L17-PC.tif]

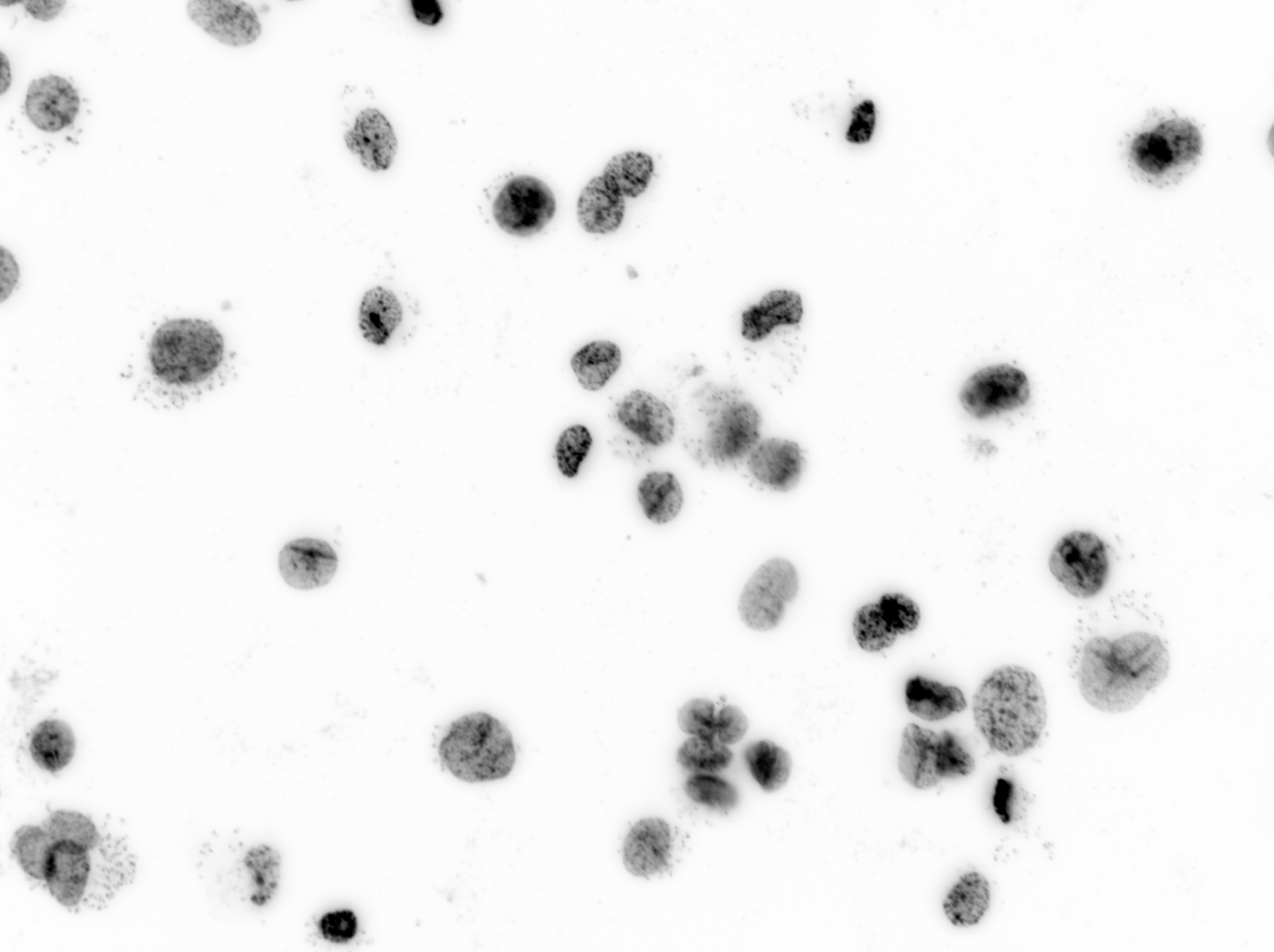

Supplement: File S2 — Transformed DAPI and Phase contrast (PC) image set of THP-1 macrophages infected by L. infantum parasites, and treated with increasing concentrations of glucantime (0-25-50-100 µg/ml). (ZIP) [file pntd.0002850.s004.zip › SI3/50/L18-DAPI.tif]

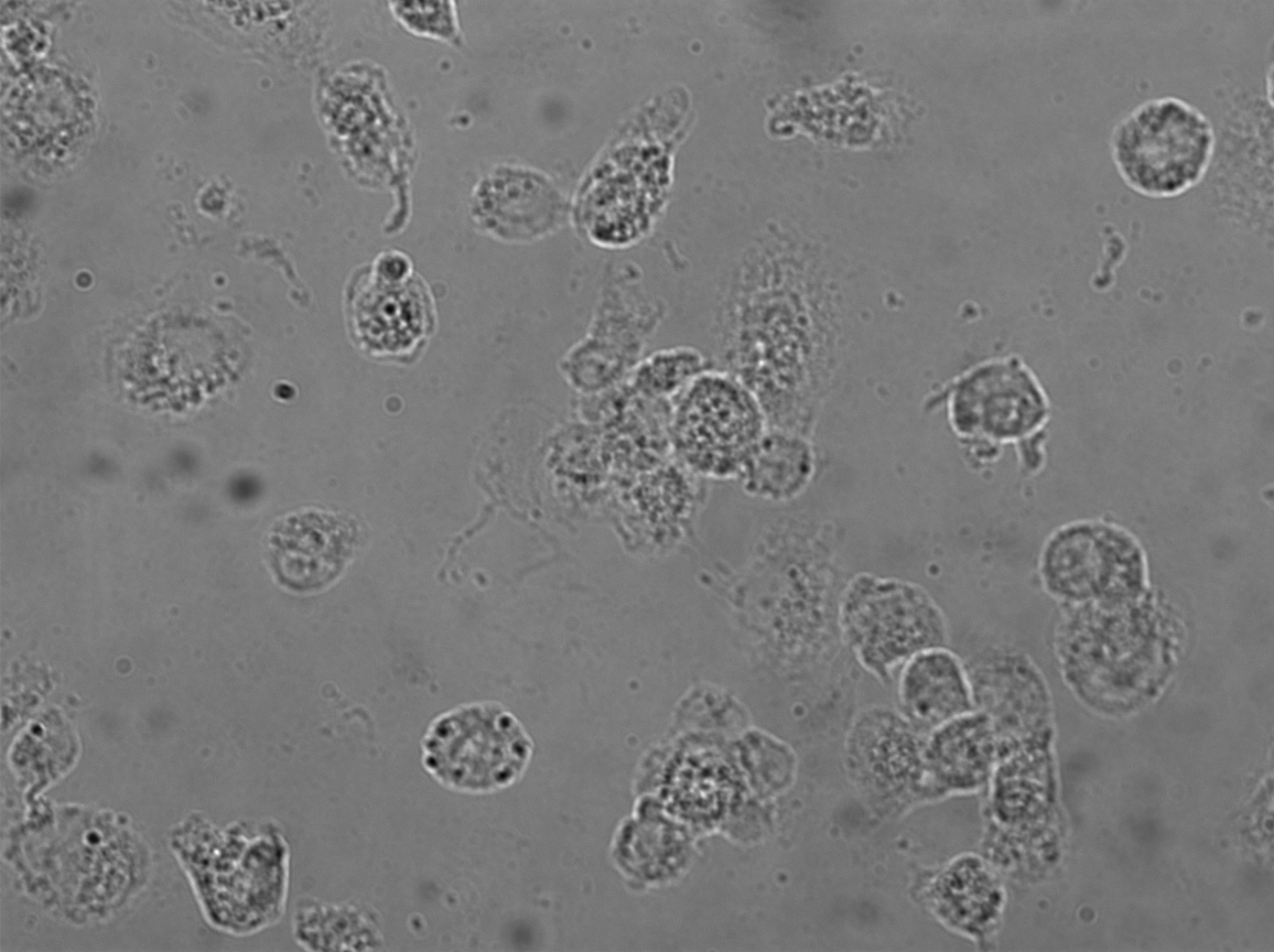

Supplement: File S2 — Transformed DAPI and Phase contrast (PC) image set of THP-1 macrophages infected by L. infantum parasites, and treated with increasing concentrations of glucantime (0-25-50-100 µg/ml). (ZIP) [file pntd.0002850.s004.zip › SI3/50/L18-PC.tif]

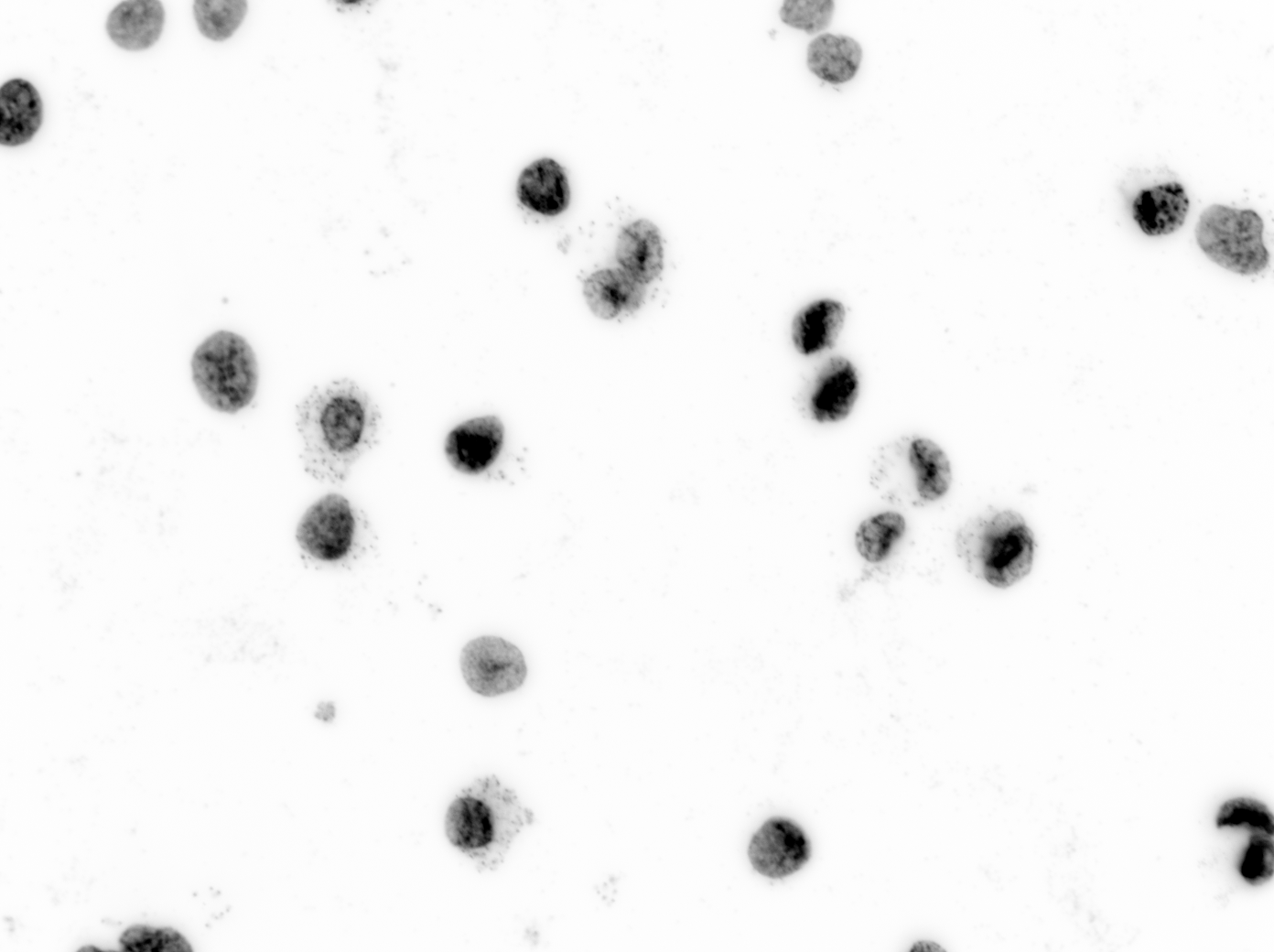

Supplement: File S2 — Transformed DAPI and Phase contrast (PC) image set of THP-1 macrophages infected by L. infantum parasites, and treated with increasing concentrations of glucantime (0-25-50-100 µg/ml). (ZIP) [file pntd.0002850.s004.zip › SI3/50/L19-DAPI.tif]

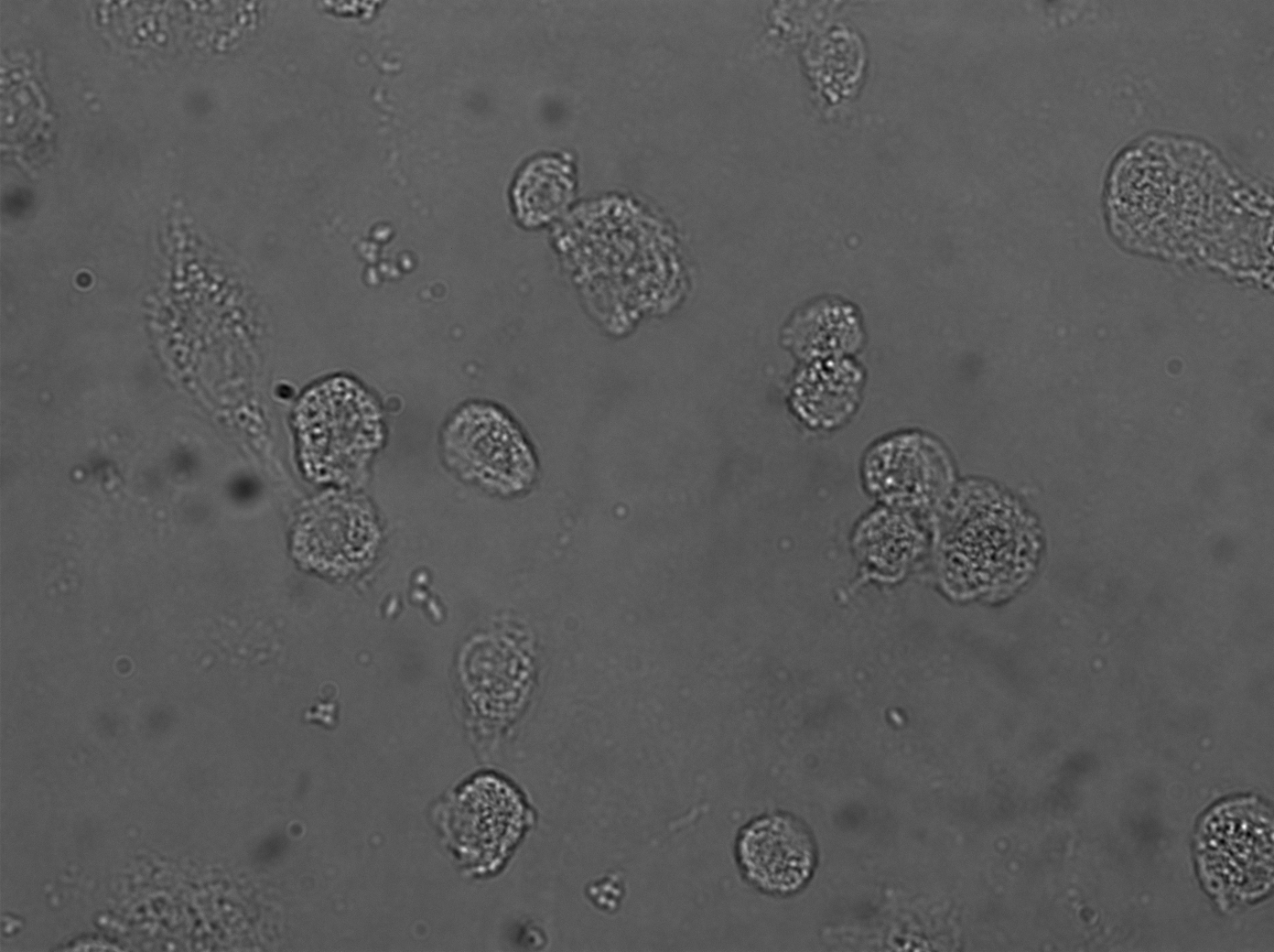

Supplement: File S2 — Transformed DAPI and Phase contrast (PC) image set of THP-1 macrophages infected by L. infantum parasites, and treated with increasing concentrations of glucantime (0-25-50-100 µg/ml). (ZIP) [file pntd.0002850.s004.zip › SI3/50/L19-PC.tif]

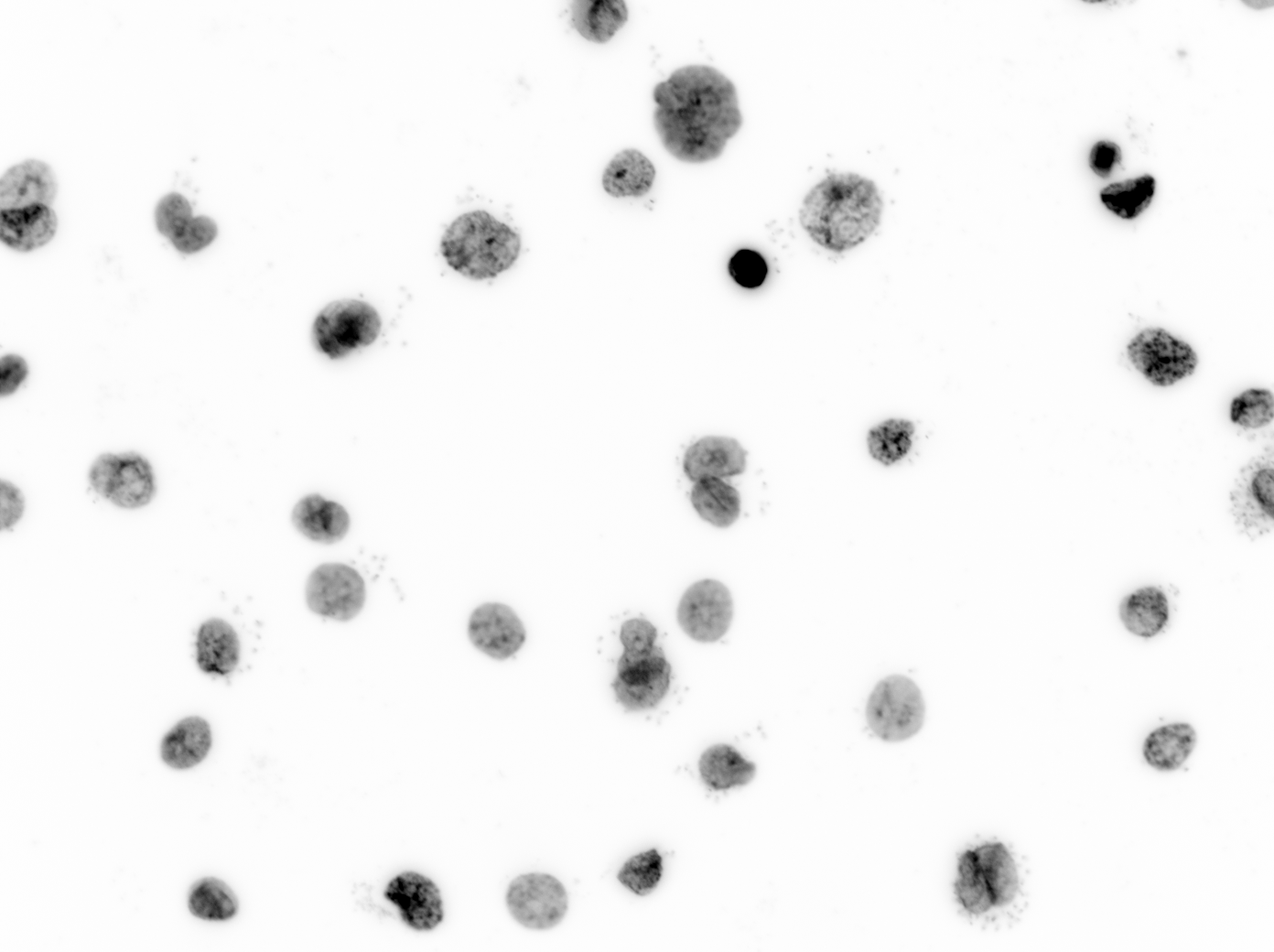

Supplement: File S2 — Transformed DAPI and Phase contrast (PC) image set of THP-1 macrophages infected by L. infantum parasites, and treated with increasing concentrations of glucantime (0-25-50-100 µg/ml). (ZIP) [file pntd.0002850.s004.zip › SI3/50/L20-DAPI.tif]

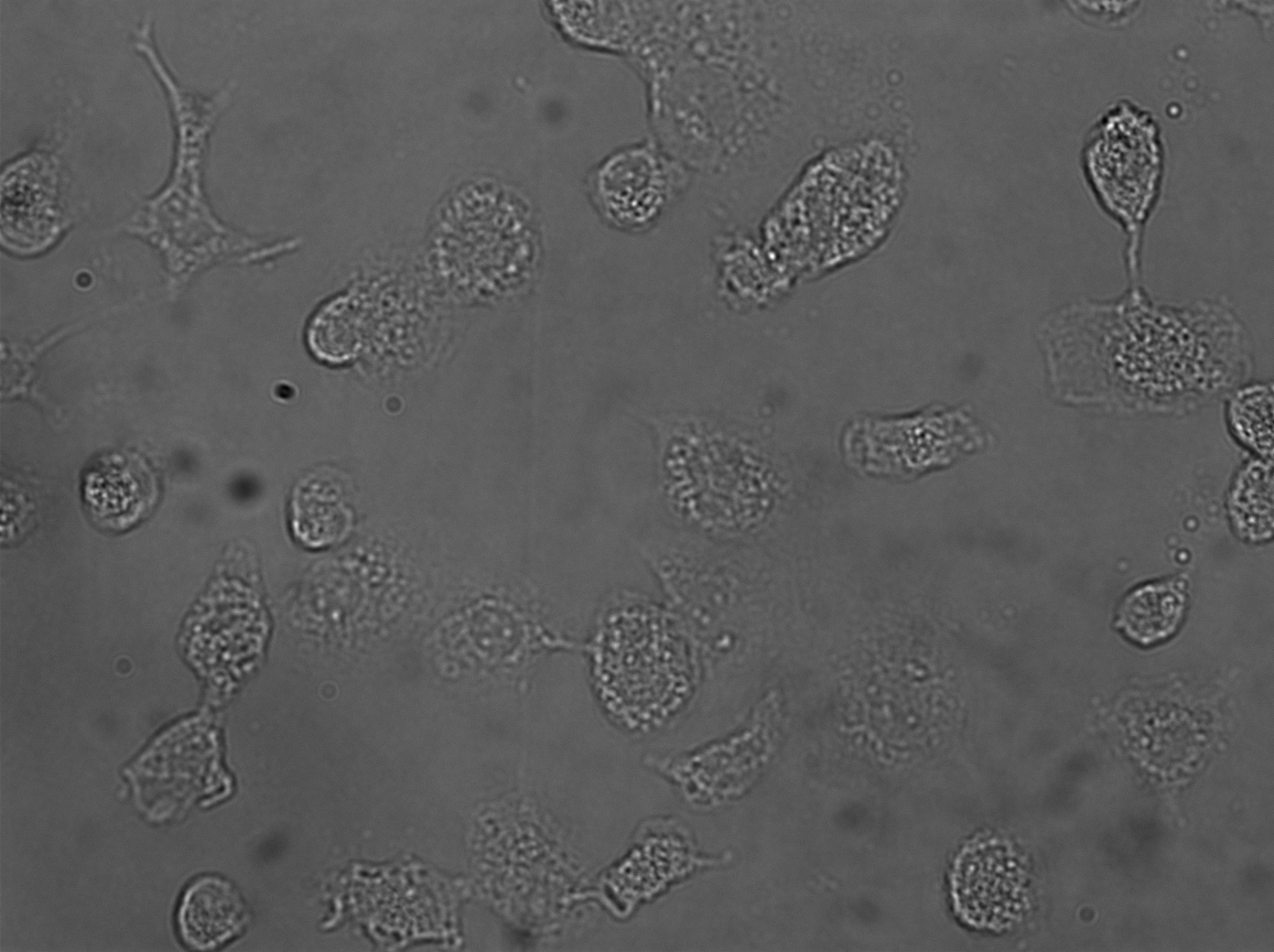

Supplement: File S2 — Transformed DAPI and Phase contrast (PC) image set of THP-1 macrophages infected by L. infantum parasites, and treated with increasing concentrations of glucantime (0-25-50-100 µg/ml). (ZIP) [file pntd.0002850.s004.zip › SI3/50/L20-PC.tif]

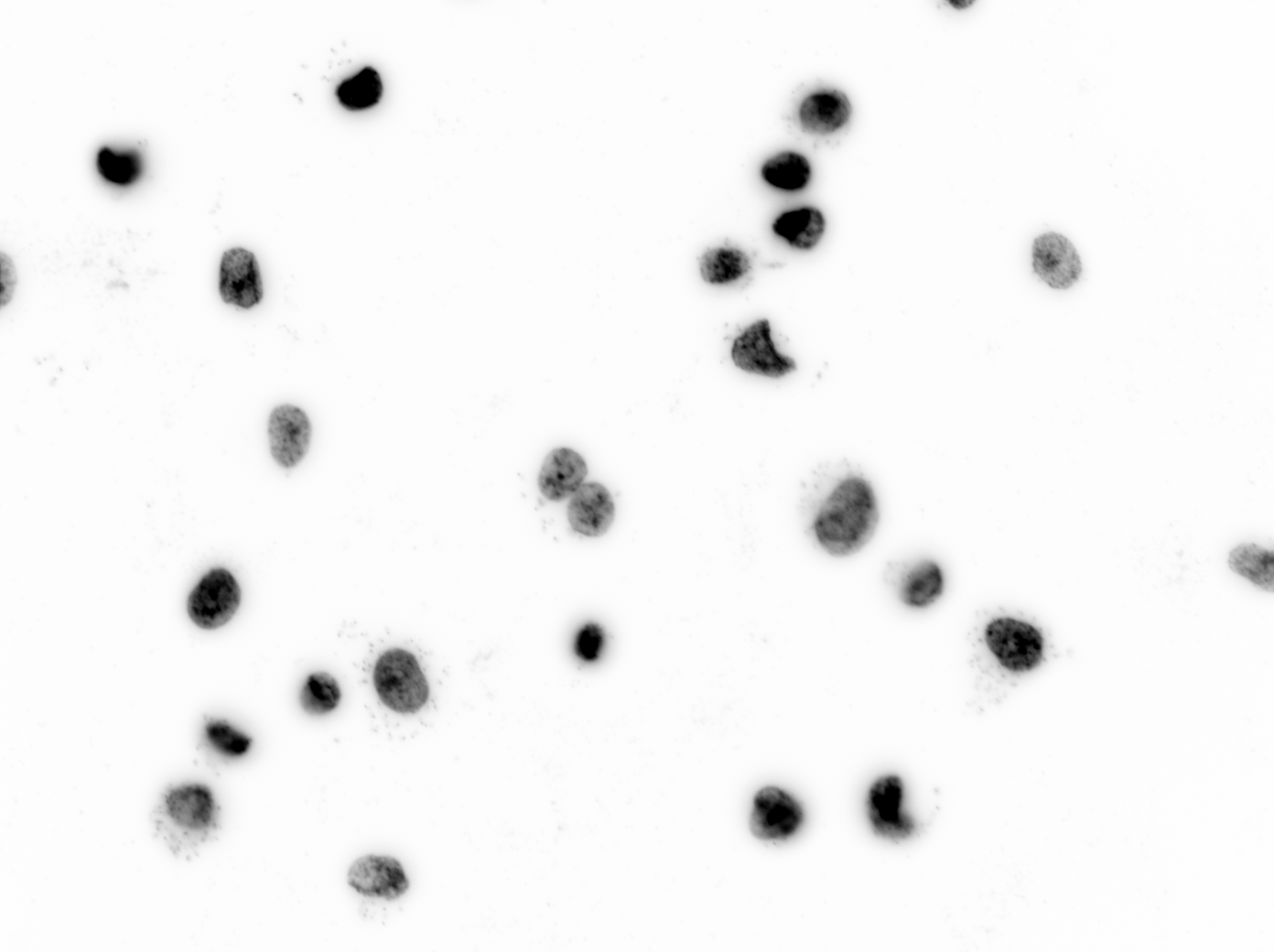

Supplement: File S2 — Transformed DAPI and Phase contrast (PC) image set of THP-1 macrophages infected by L. infantum parasites, and treated with increasing concentrations of glucantime (0-25-50-100 µg/ml). (ZIP) [file pntd.0002850.s004.zip › SI3/50/L21-DAPI.tif]

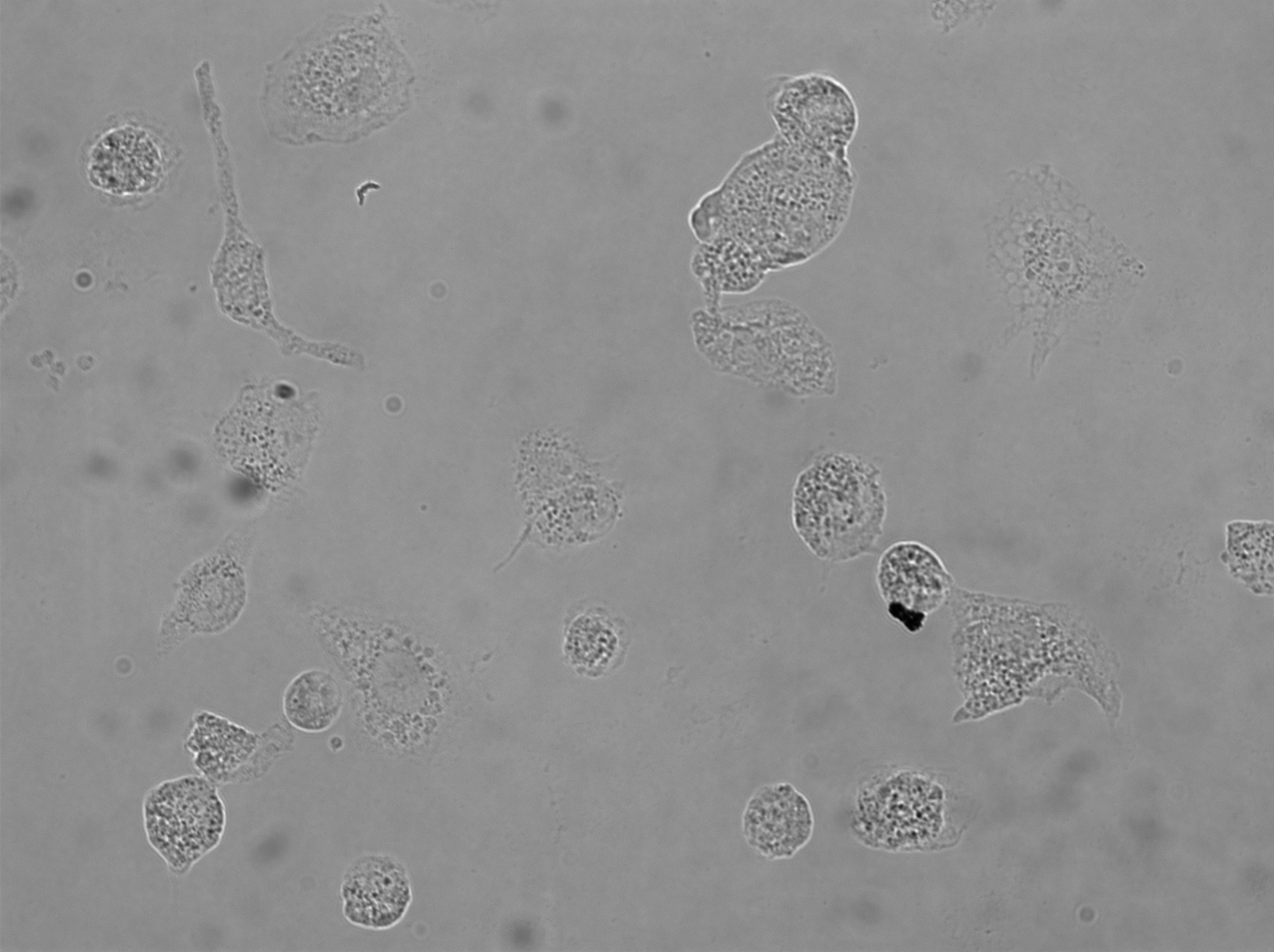

Supplement: File S2 — Transformed DAPI and Phase contrast (PC) image set of THP-1 macrophages infected by L. infantum parasites, and treated with increasing concentrations of glucantime (0-25-50-100 µg/ml). (ZIP) [file pntd.0002850.s004.zip › SI3/50/L21-PC.tif]

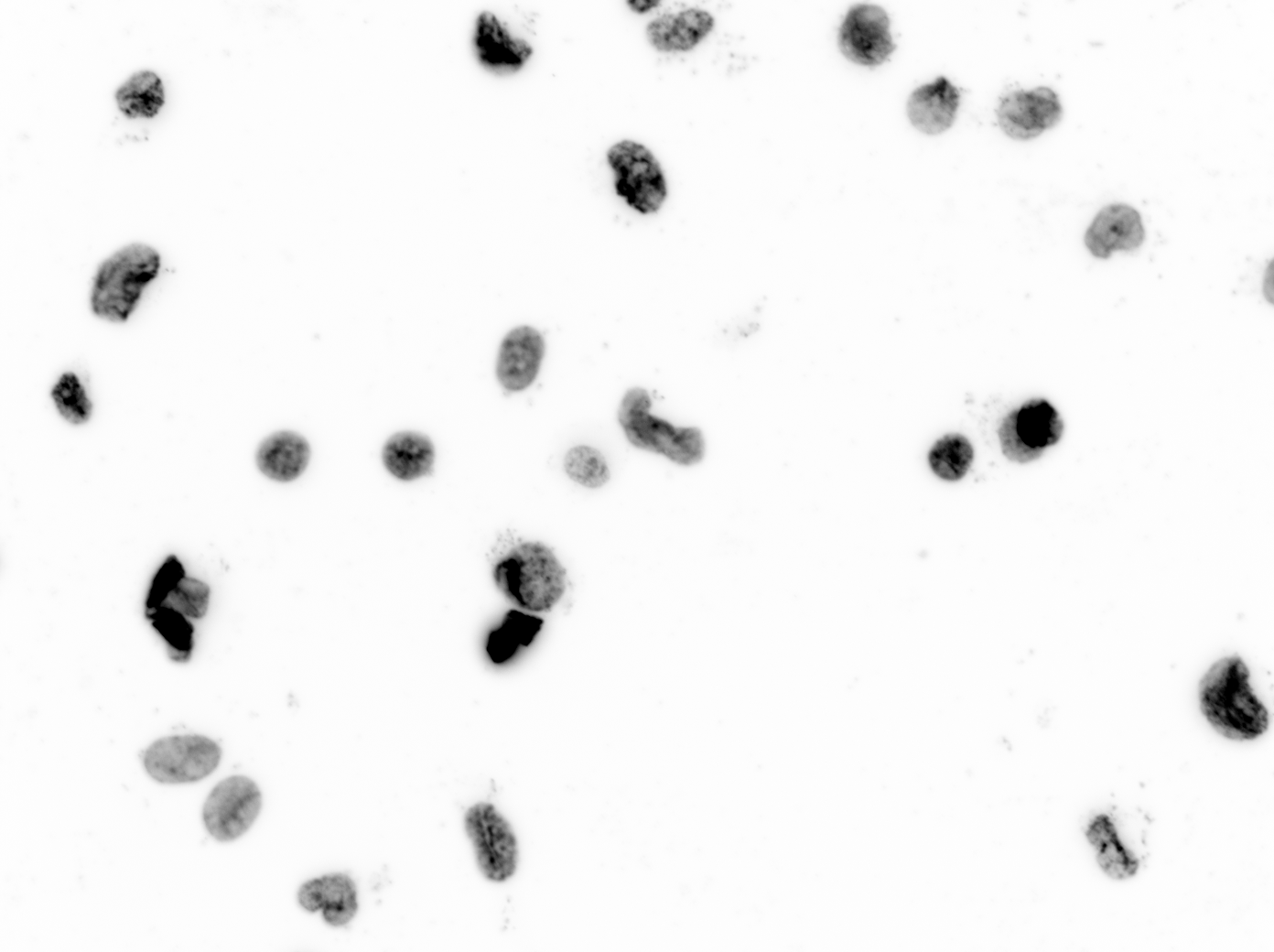

Supplement: File S2 — Transformed DAPI and Phase contrast (PC) image set of THP-1 macrophages infected by L. infantum parasites, and treated with increasing concentrations of glucantime (0-25-50-100 µg/ml). (ZIP) [file pntd.0002850.s004.zip › SI3/50/L22-DAPI.tif]

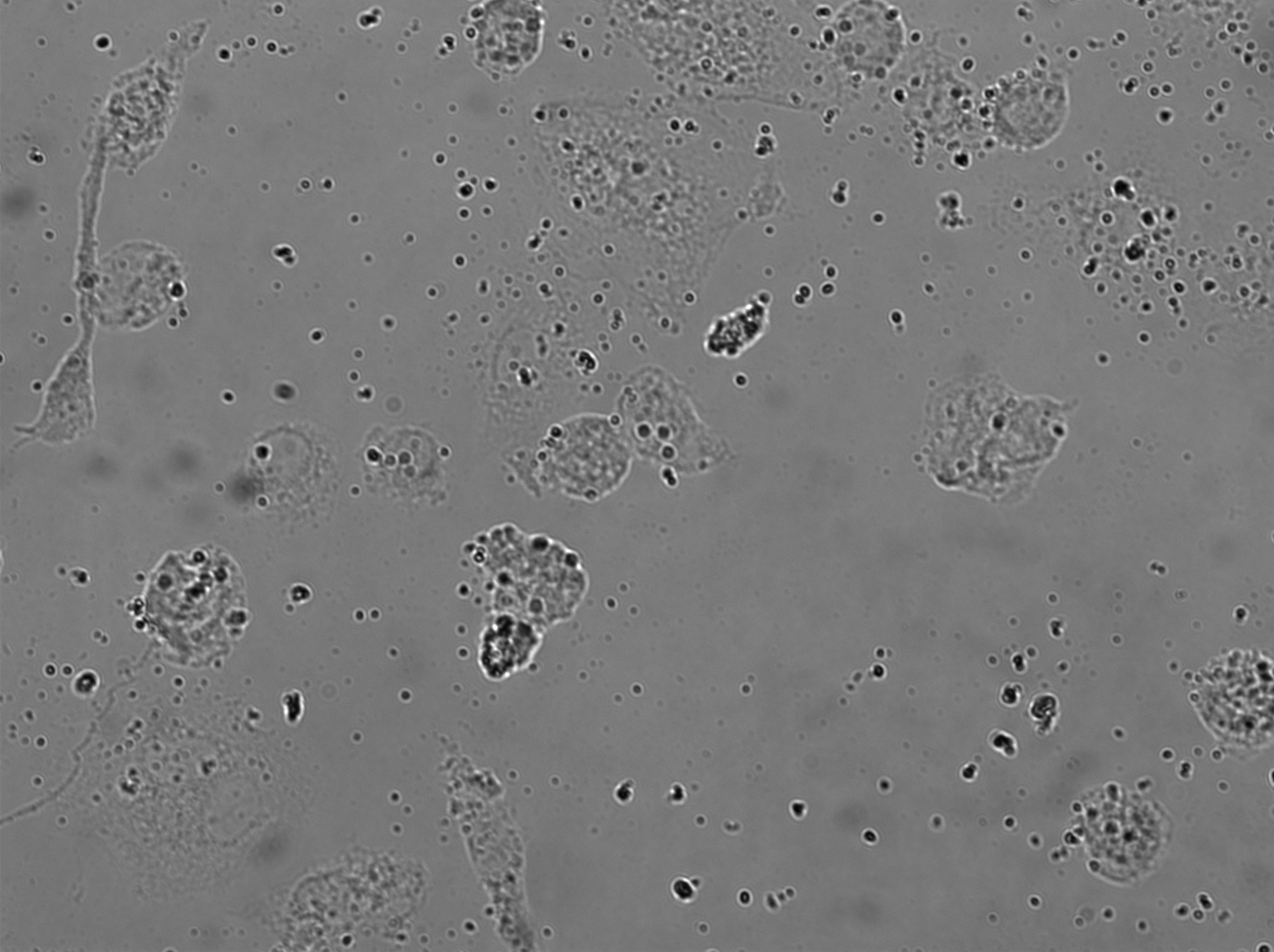

Supplement: File S2 — Transformed DAPI and Phase contrast (PC) image set of THP-1 macrophages infected by L. infantum parasites, and treated with increasing concentrations of glucantime (0-25-50-100 µg/ml). (ZIP) [file pntd.0002850.s004.zip › SI3/50/L22-PC.tif]

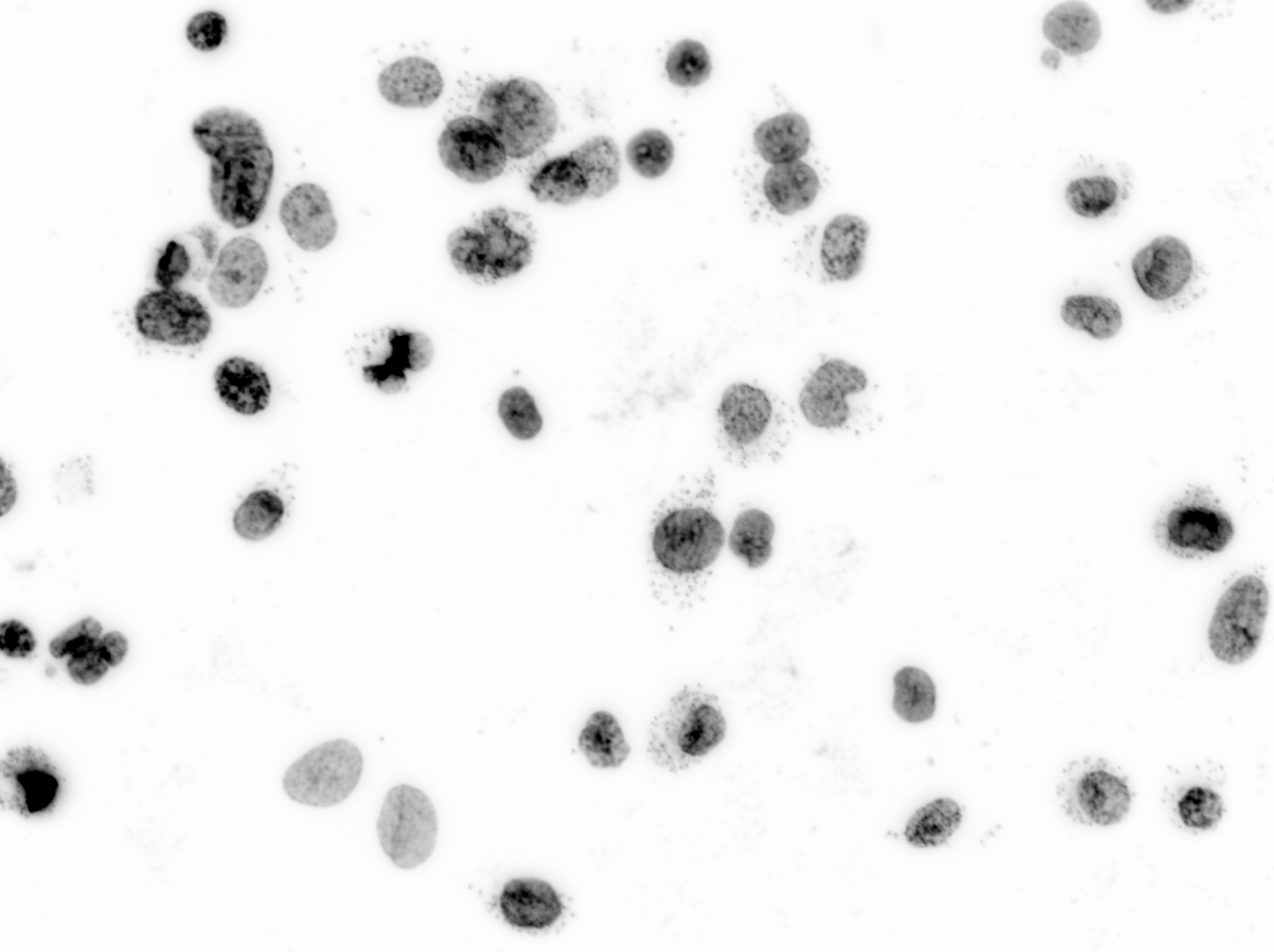

Supplement: File S2 — Transformed DAPI and Phase contrast (PC) image set of THP-1 macrophages infected by L. infantum parasites, and treated with increasing concentrations of glucantime (0-25-50-100 µg/ml). (ZIP) [file pntd.0002850.s004.zip › SI3/50/L23-DAPI.tif]

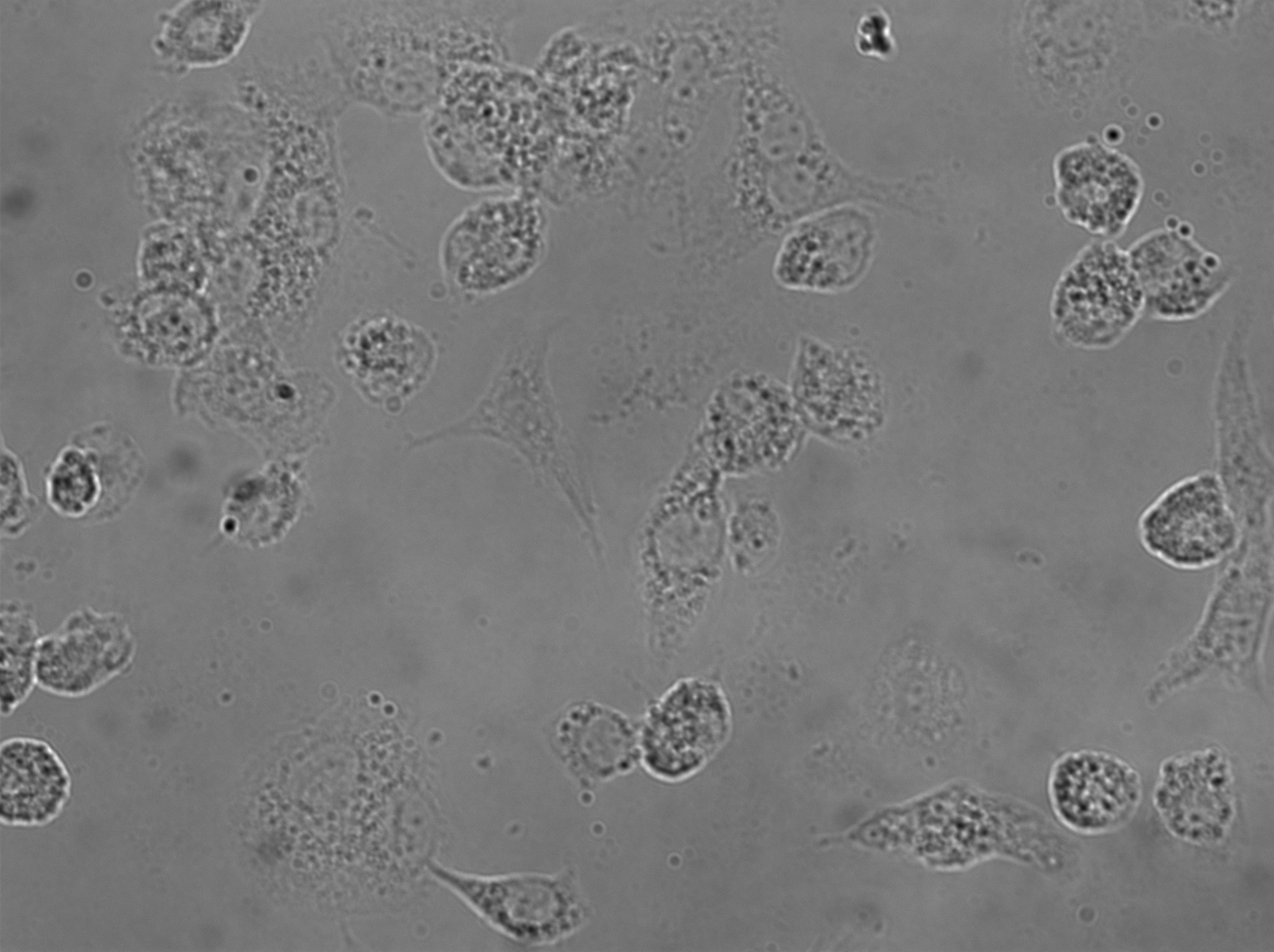

Supplement: File S2 — Transformed DAPI and Phase contrast (PC) image set of THP-1 macrophages infected by L. infantum parasites, and treated with increasing concentrations of glucantime (0-25-50-100 µg/ml). (ZIP) [file pntd.0002850.s004.zip › SI3/50/L23-PC.tif]

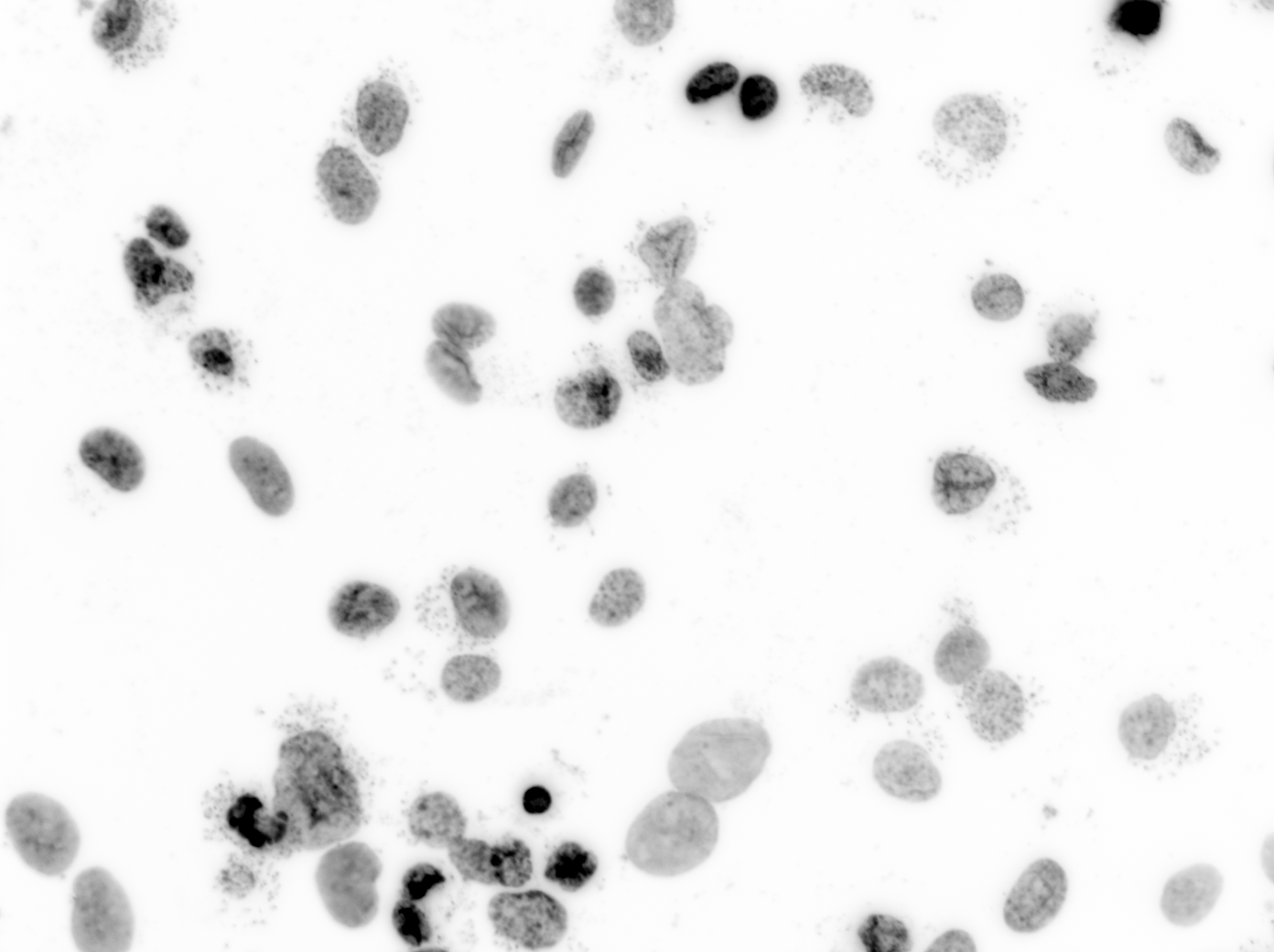

Supplement: File S2 — Transformed DAPI and Phase contrast (PC) image set of THP-1 macrophages infected by L. infantum parasites, and treated with increasing concentrations of glucantime (0-25-50-100 µg/ml). (ZIP) [file pntd.0002850.s004.zip › SI3/untreated cells/L1-DAPI.tif]

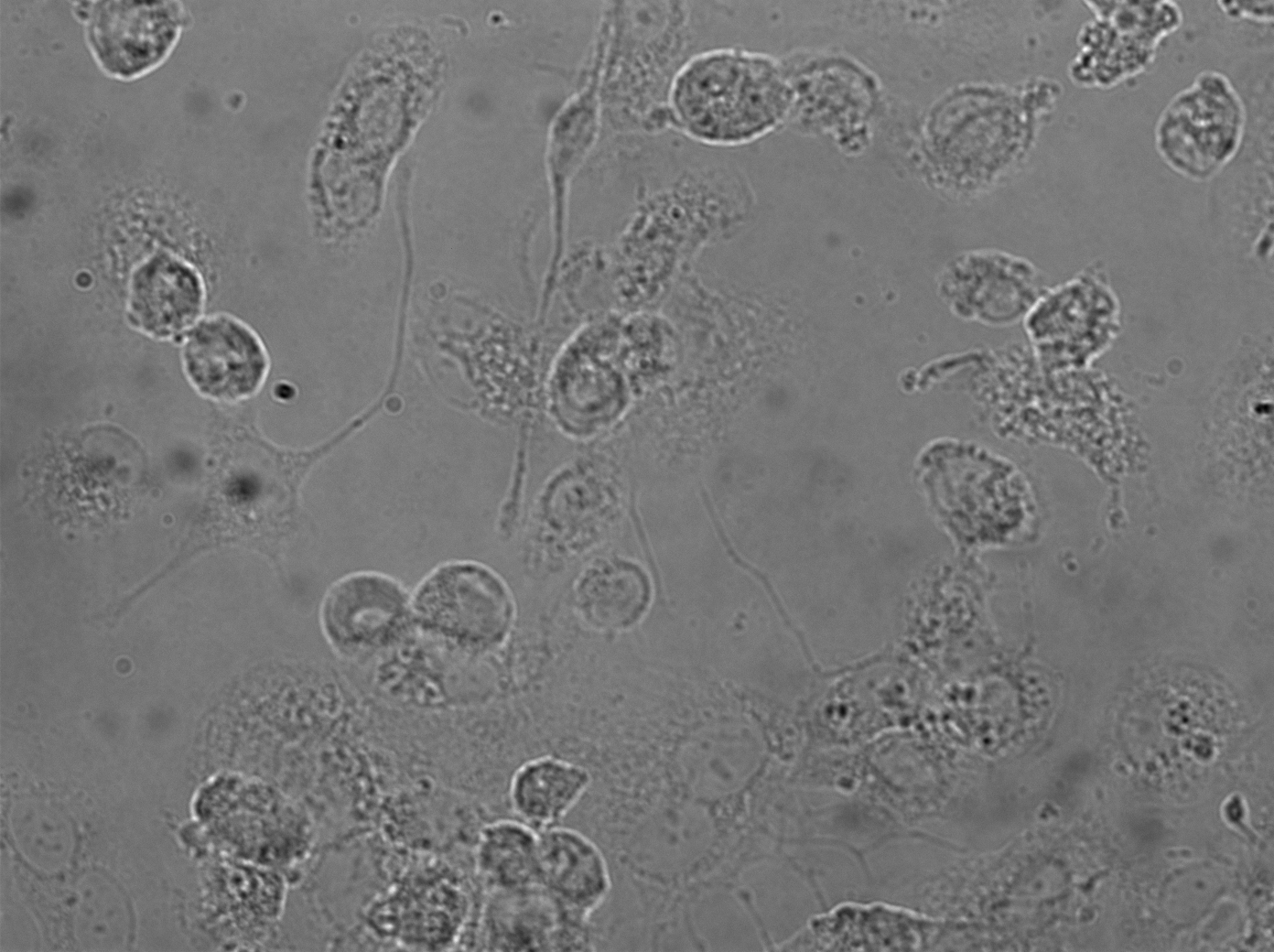

Supplement: File S2 — Transformed DAPI and Phase contrast (PC) image set of THP-1 macrophages infected by L. infantum parasites, and treated with increasing concentrations of glucantime (0-25-50-100 µg/ml). (ZIP) [file pntd.0002850.s004.zip › SI3/untreated cells/L1-PC.tif]

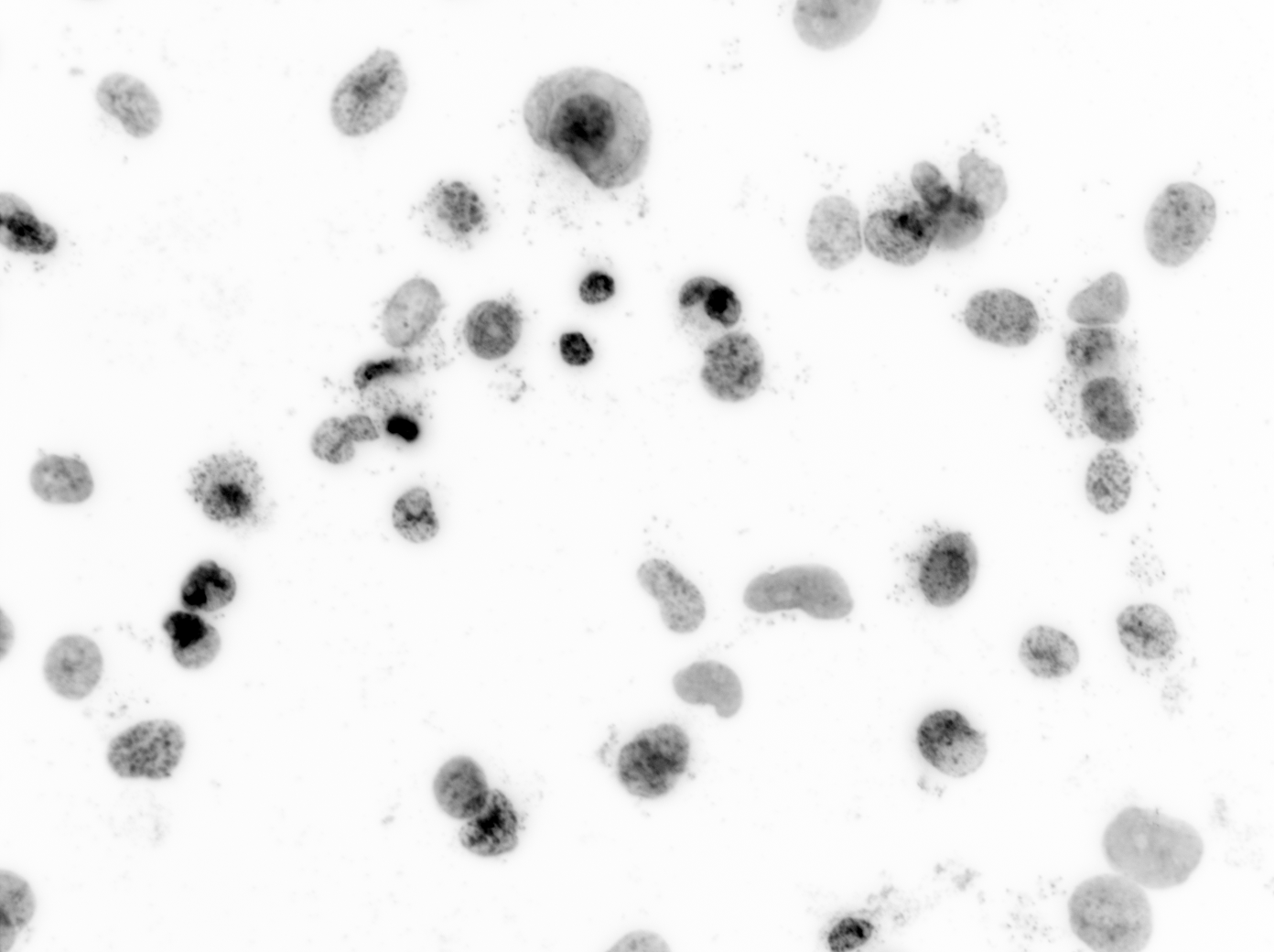

Supplement: File S2 — Transformed DAPI and Phase contrast (PC) image set of THP-1 macrophages infected by L. infantum parasites, and treated with increasing concentrations of glucantime (0-25-50-100 µg/ml). (ZIP) [file pntd.0002850.s004.zip › SI3/untreated cells/L2-DAPI.tif]

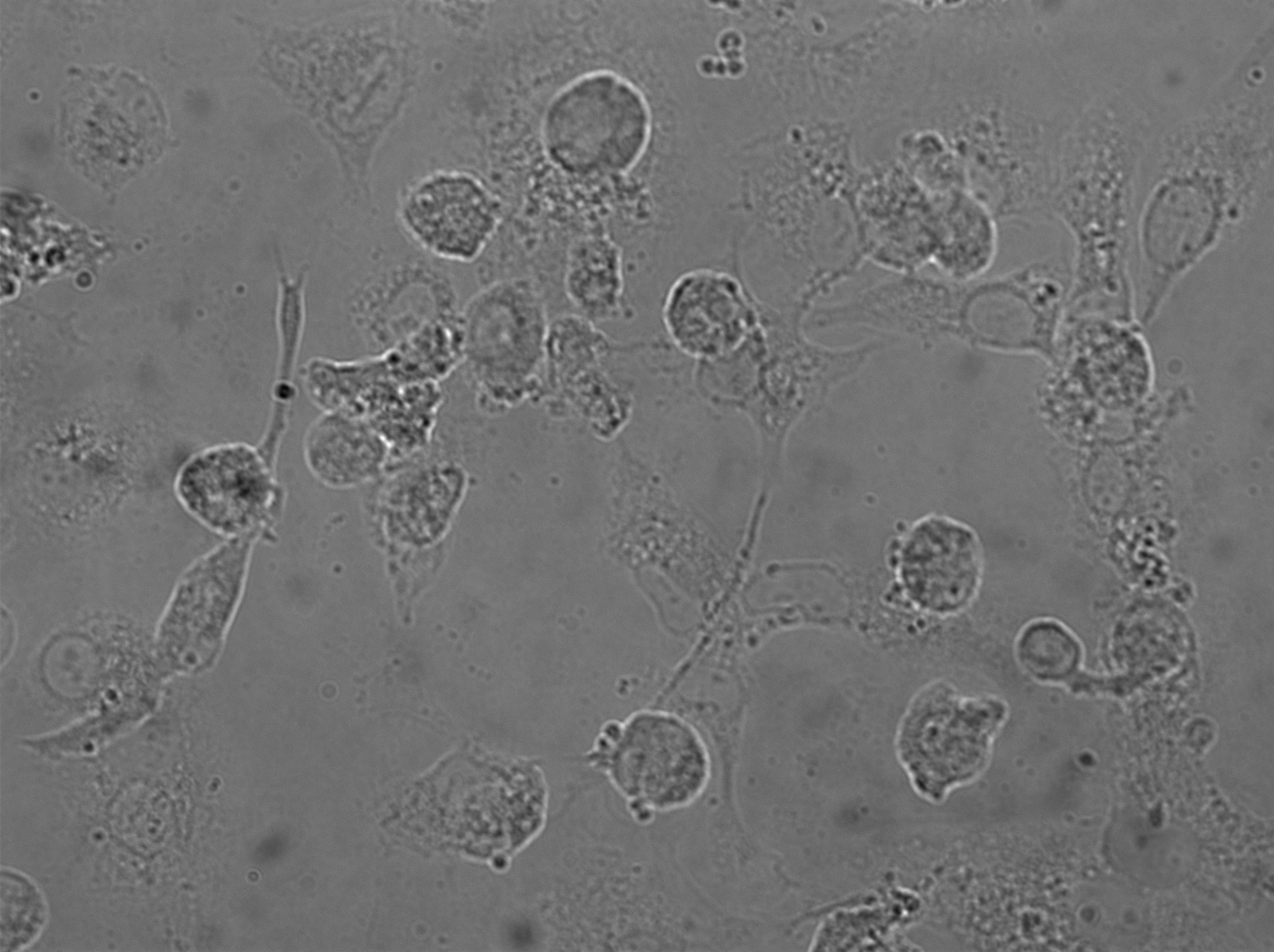

Supplement: File S2 — Transformed DAPI and Phase contrast (PC) image set of THP-1 macrophages infected by L. infantum parasites, and treated with increasing concentrations of glucantime (0-25-50-100 µg/ml). (ZIP) [file pntd.0002850.s004.zip › SI3/untreated cells/L2-PC.tif]

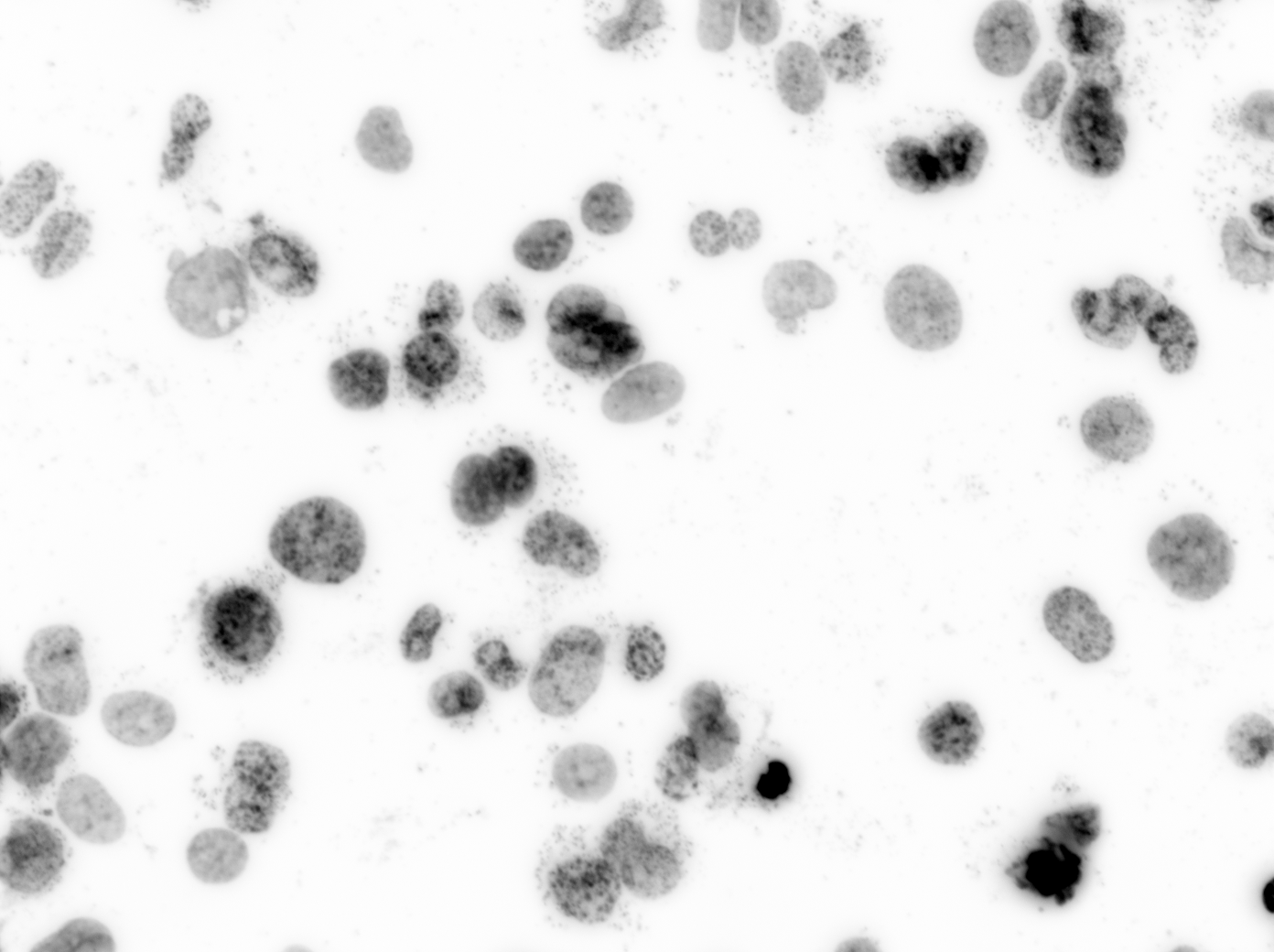

Supplement: File S2 — Transformed DAPI and Phase contrast (PC) image set of THP-1 macrophages infected by L. infantum parasites, and treated with increasing concentrations of glucantime (0-25-50-100 µg/ml). (ZIP) [file pntd.0002850.s004.zip › SI3/untreated cells/L3-DAPI.tif]

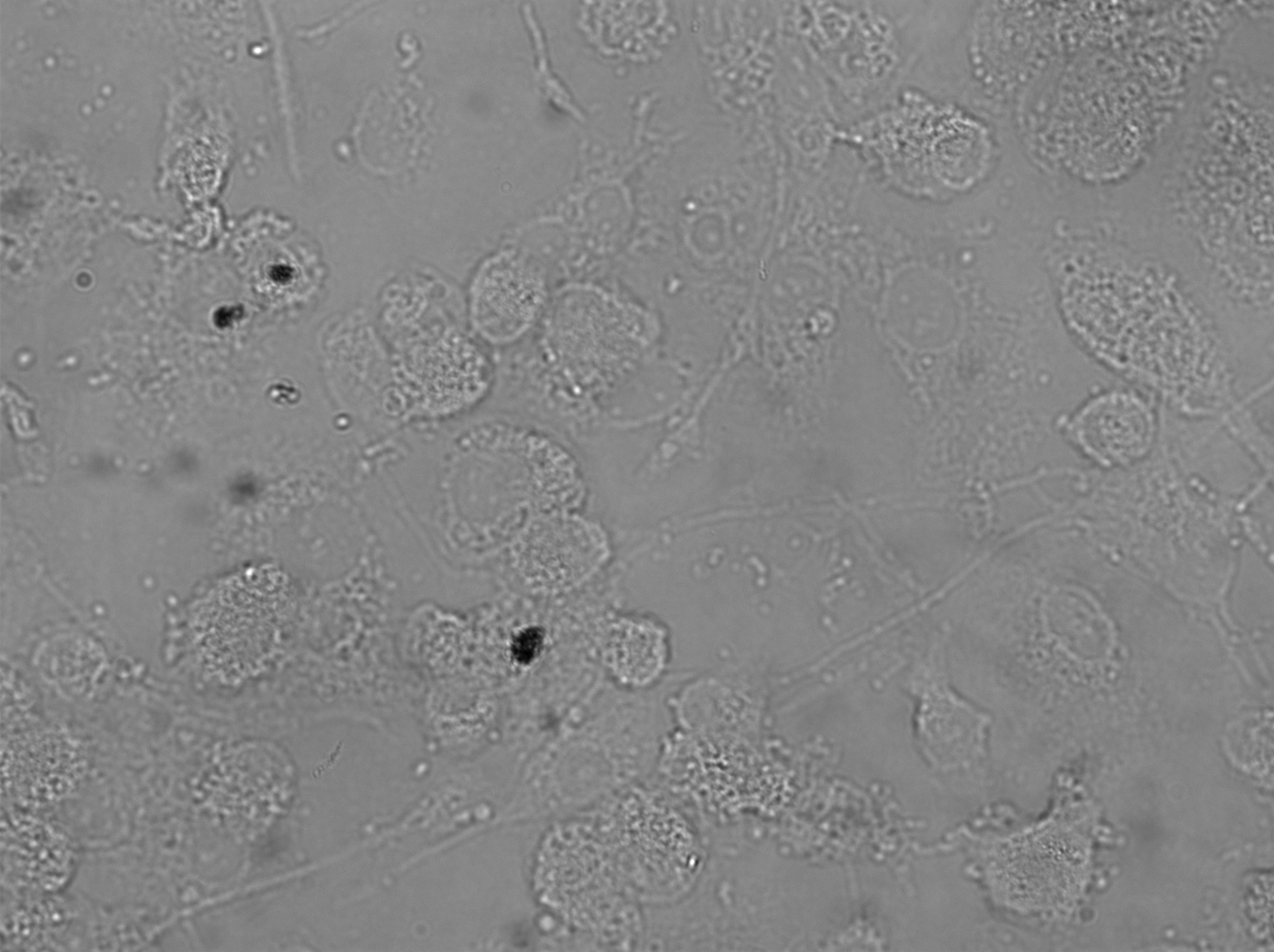

Supplement: File S2 — Transformed DAPI and Phase contrast (PC) image set of THP-1 macrophages infected by L. infantum parasites, and treated with increasing concentrations of glucantime (0-25-50-100 µg/ml). (ZIP) [file pntd.0002850.s004.zip › SI3/untreated cells/L3-PC.tif]

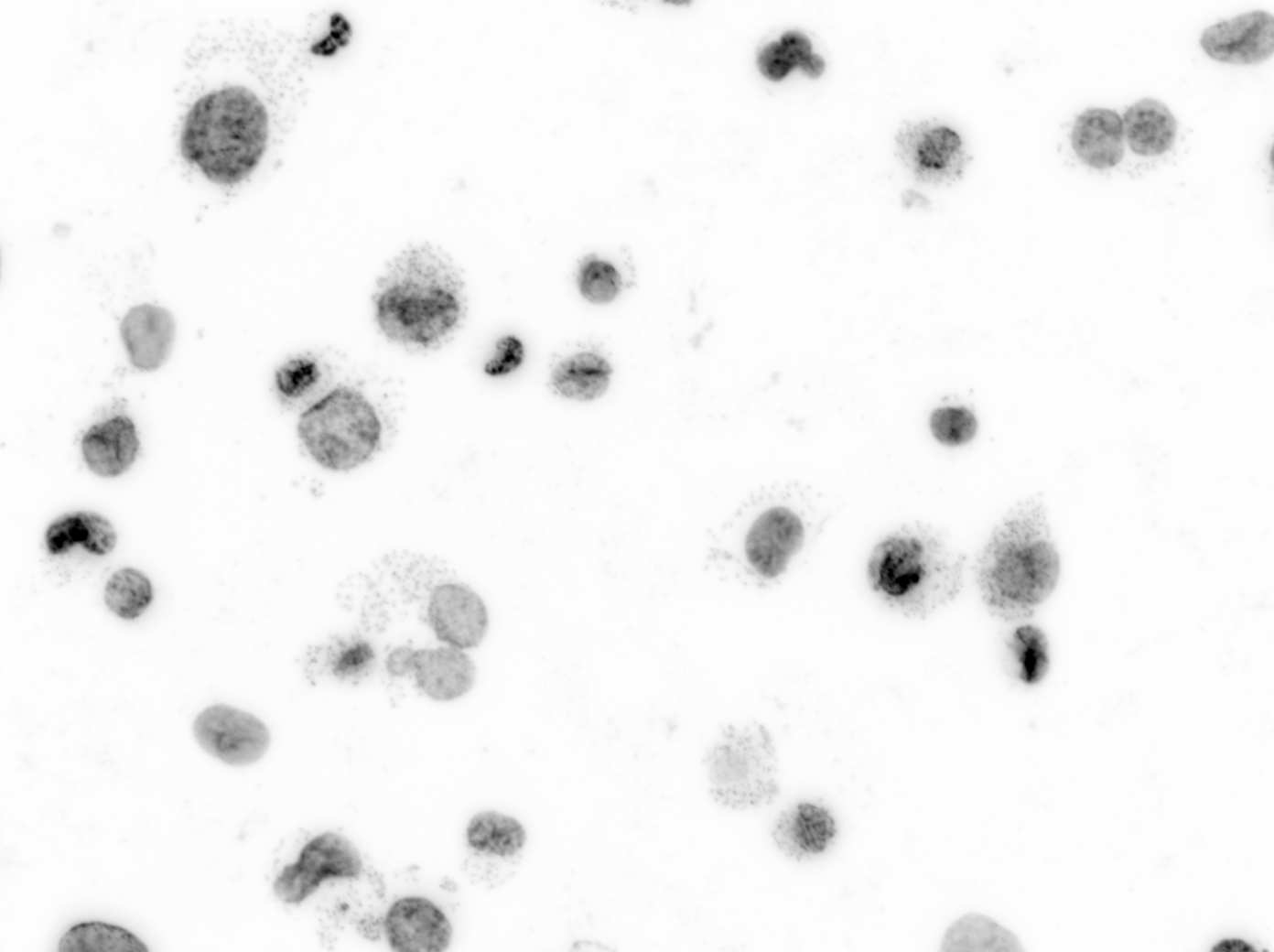

Supplement: File S2 — Transformed DAPI and Phase contrast (PC) image set of THP-1 macrophages infected by L. infantum parasites, and treated with increasing concentrations of glucantime (0-25-50-100 µg/ml). (ZIP) [file pntd.0002850.s004.zip › SI3/untreated cells/L4-DAPI.tif]

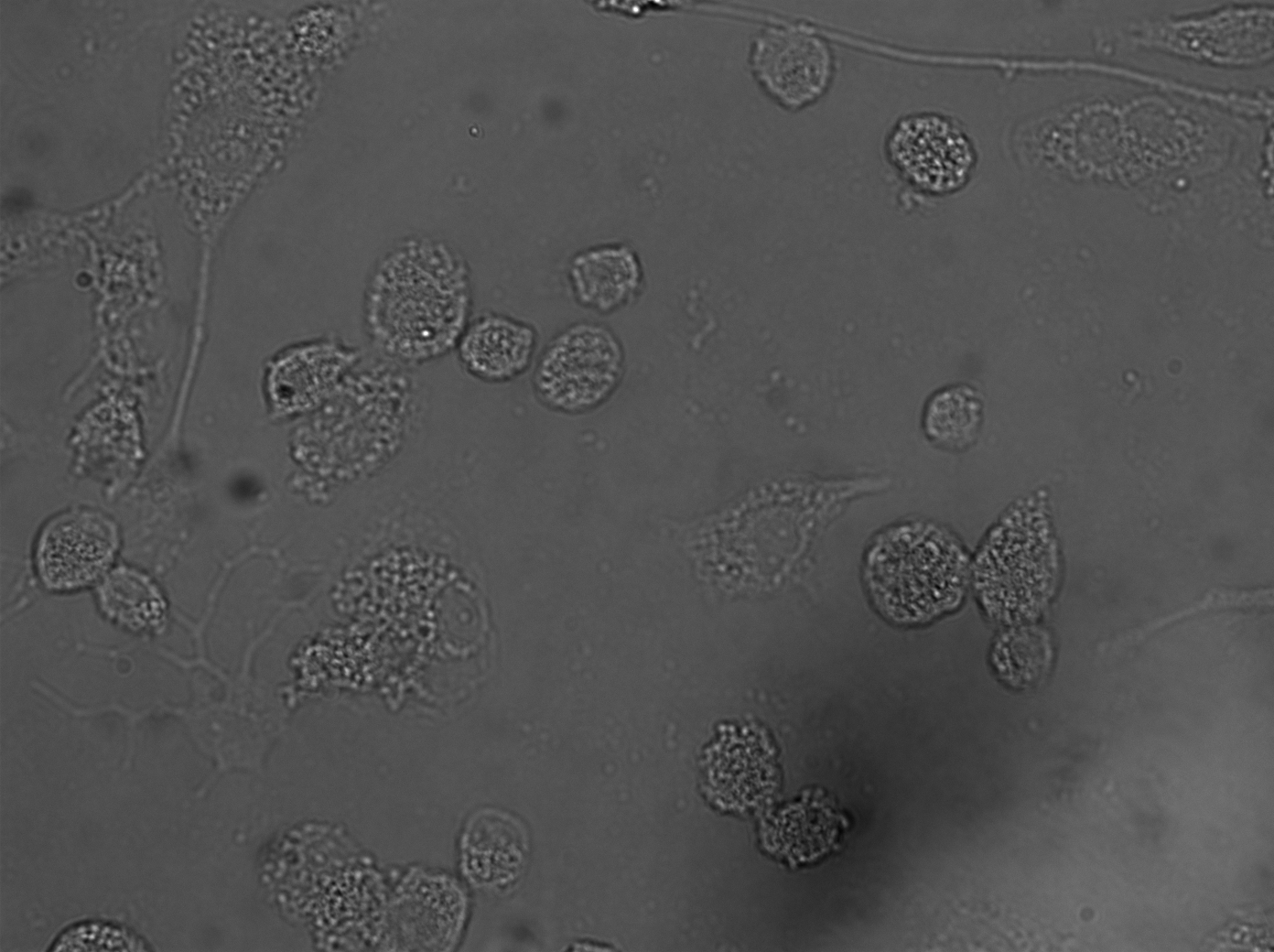

Supplement: File S2 — Transformed DAPI and Phase contrast (PC) image set of THP-1 macrophages infected by L. infantum parasites, and treated with increasing concentrations of glucantime (0-25-50-100 µg/ml). (ZIP) [file pntd.0002850.s004.zip › SI3/untreated cells/L4-PC.tif]

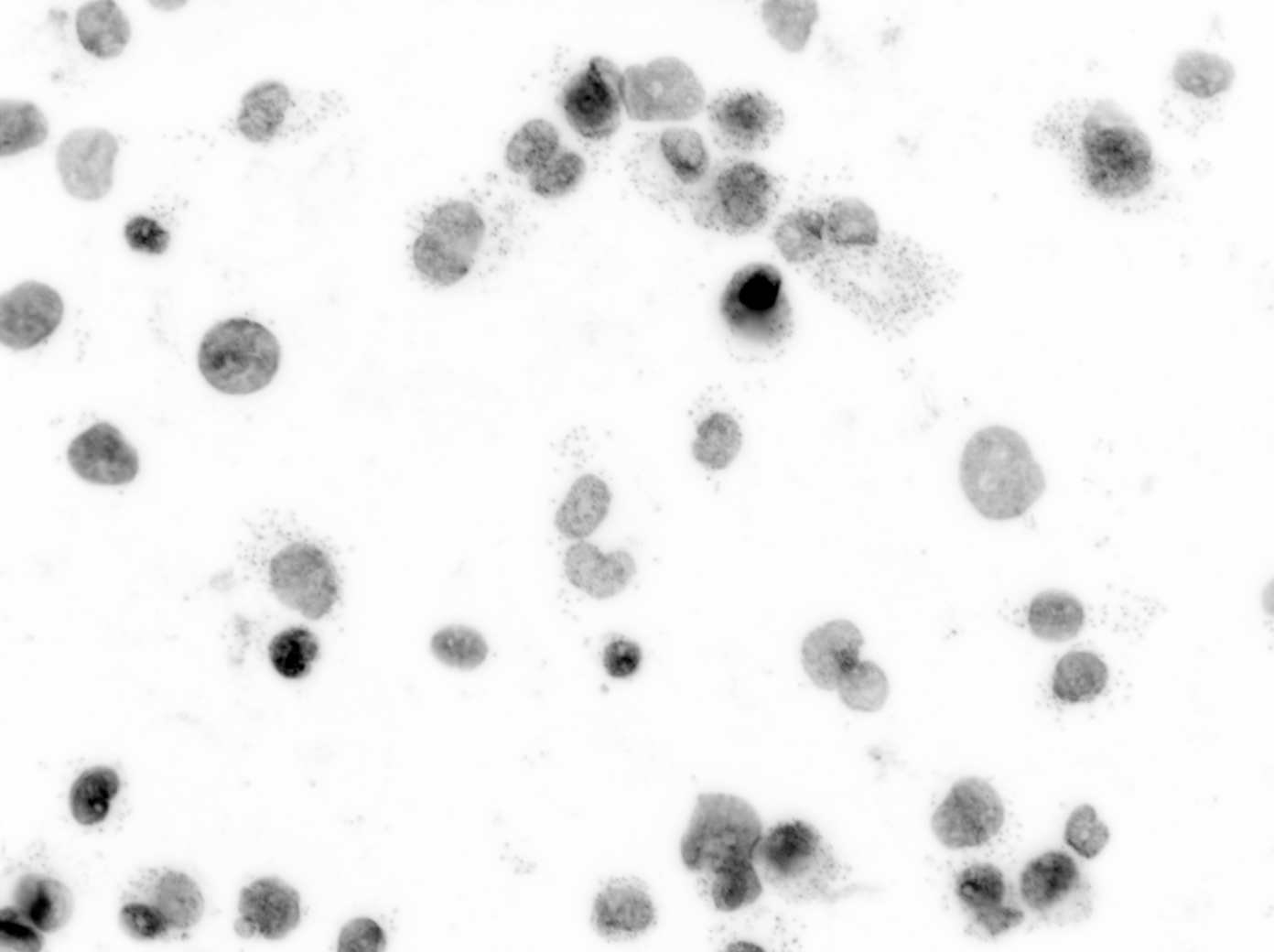

Supplement: File S2 — Transformed DAPI and Phase contrast (PC) image set of THP-1 macrophages infected by L. infantum parasites, and treated with increasing concentrations of glucantime (0-25-50-100 µg/ml). (ZIP) [file pntd.0002850.s004.zip › SI3/untreated cells/L5-DAPI.tif]

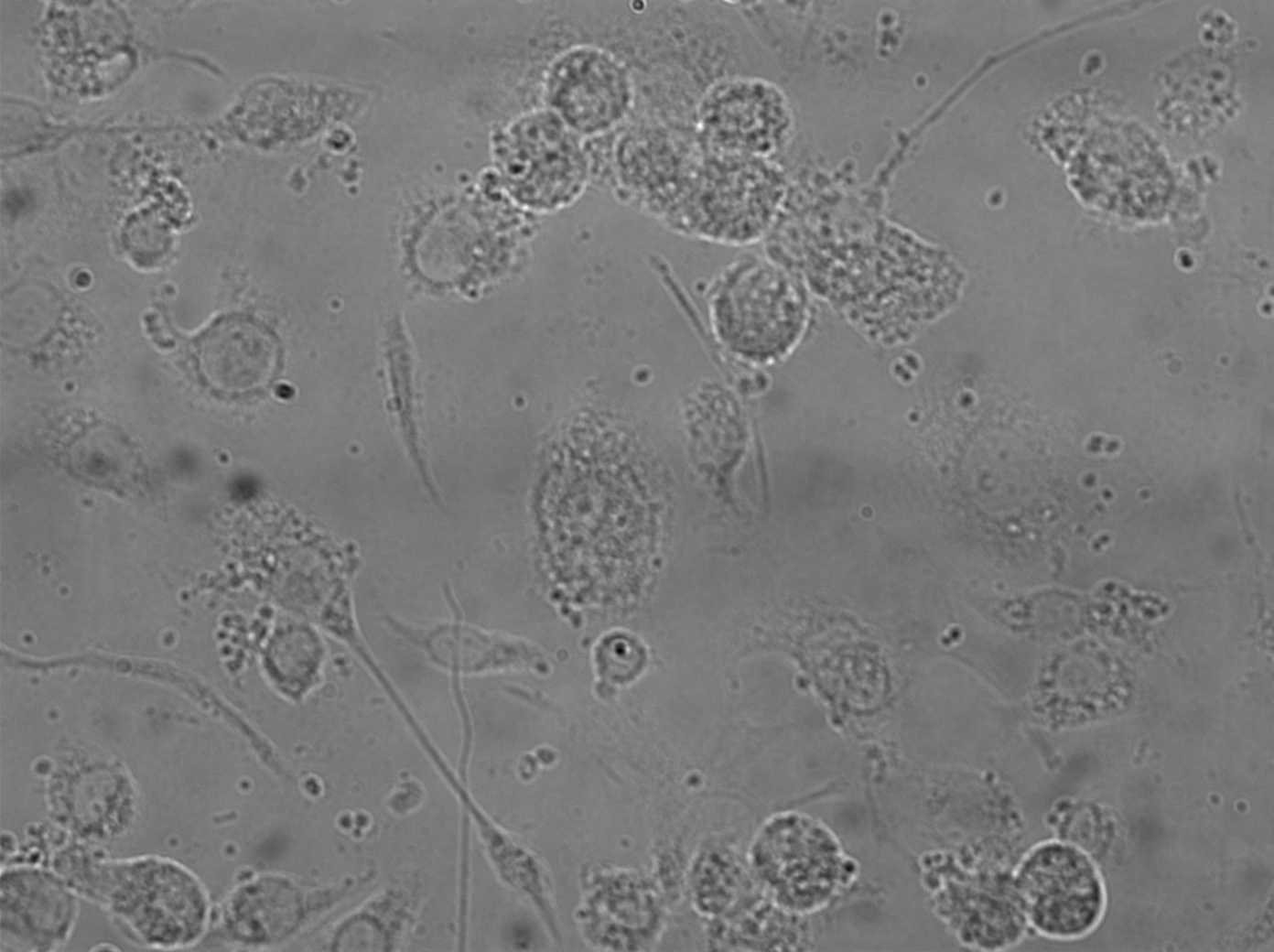

Supplement: File S2 — Transformed DAPI and Phase contrast (PC) image set of THP-1 macrophages infected by L. infantum parasites, and treated with increasing concentrations of glucantime (0-25-50-100 µg/ml). (ZIP) [file pntd.0002850.s004.zip › SI3/untreated cells/L5-PC.tif]

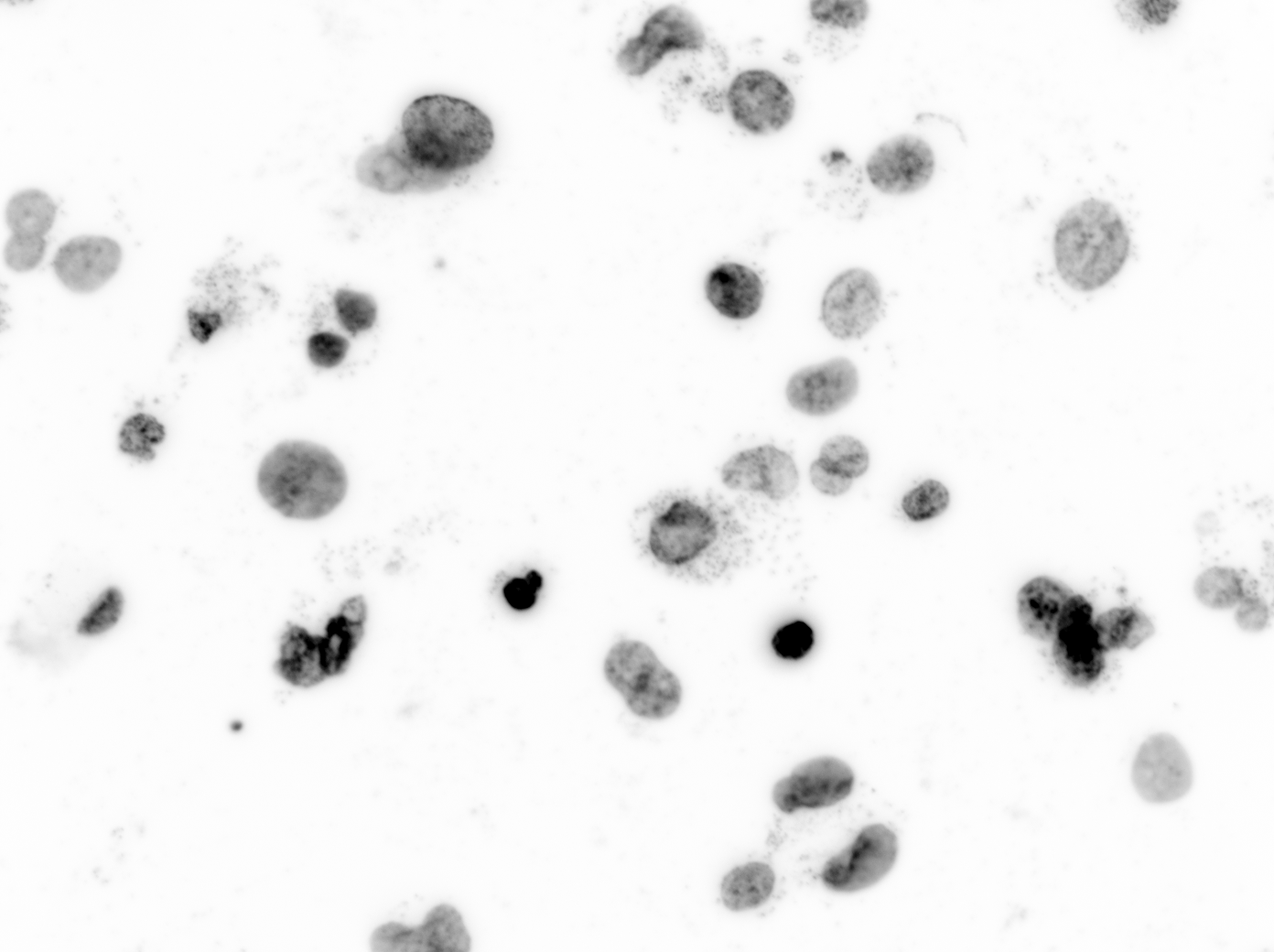

Supplement: File S2 — Transformed DAPI and Phase contrast (PC) image set of THP-1 macrophages infected by L. infantum parasites, and treated with increasing concentrations of glucantime (0-25-50-100 µg/ml). (ZIP) [file pntd.0002850.s004.zip › SI3/untreated cells/L6-DAPI.tif]

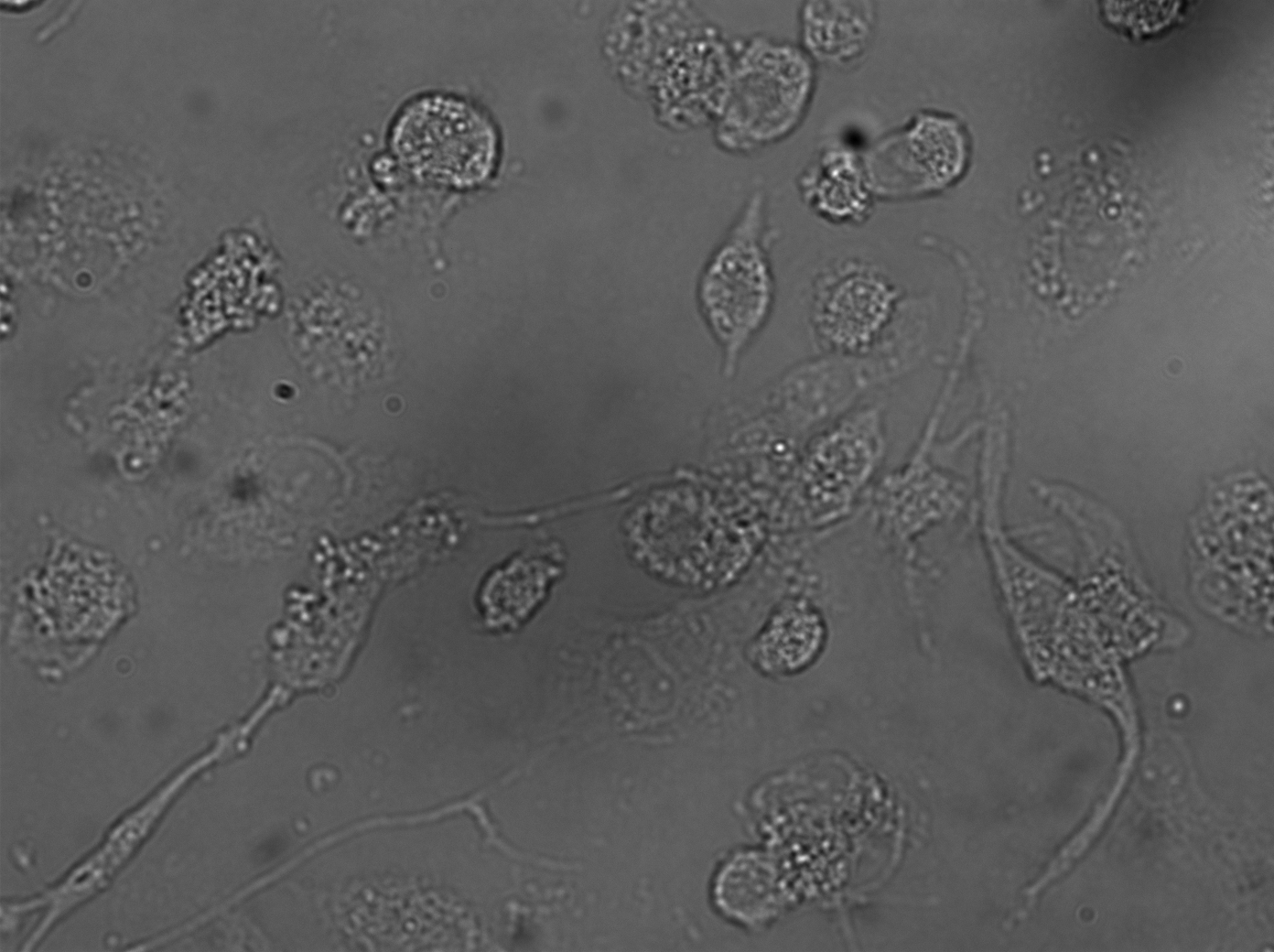

Supplement: File S2 — Transformed DAPI and Phase contrast (PC) image set of THP-1 macrophages infected by L. infantum parasites, and treated with increasing concentrations of glucantime (0-25-50-100 µg/ml). (ZIP) [file pntd.0002850.s004.zip › SI3/untreated cells/L6-PC.tif]

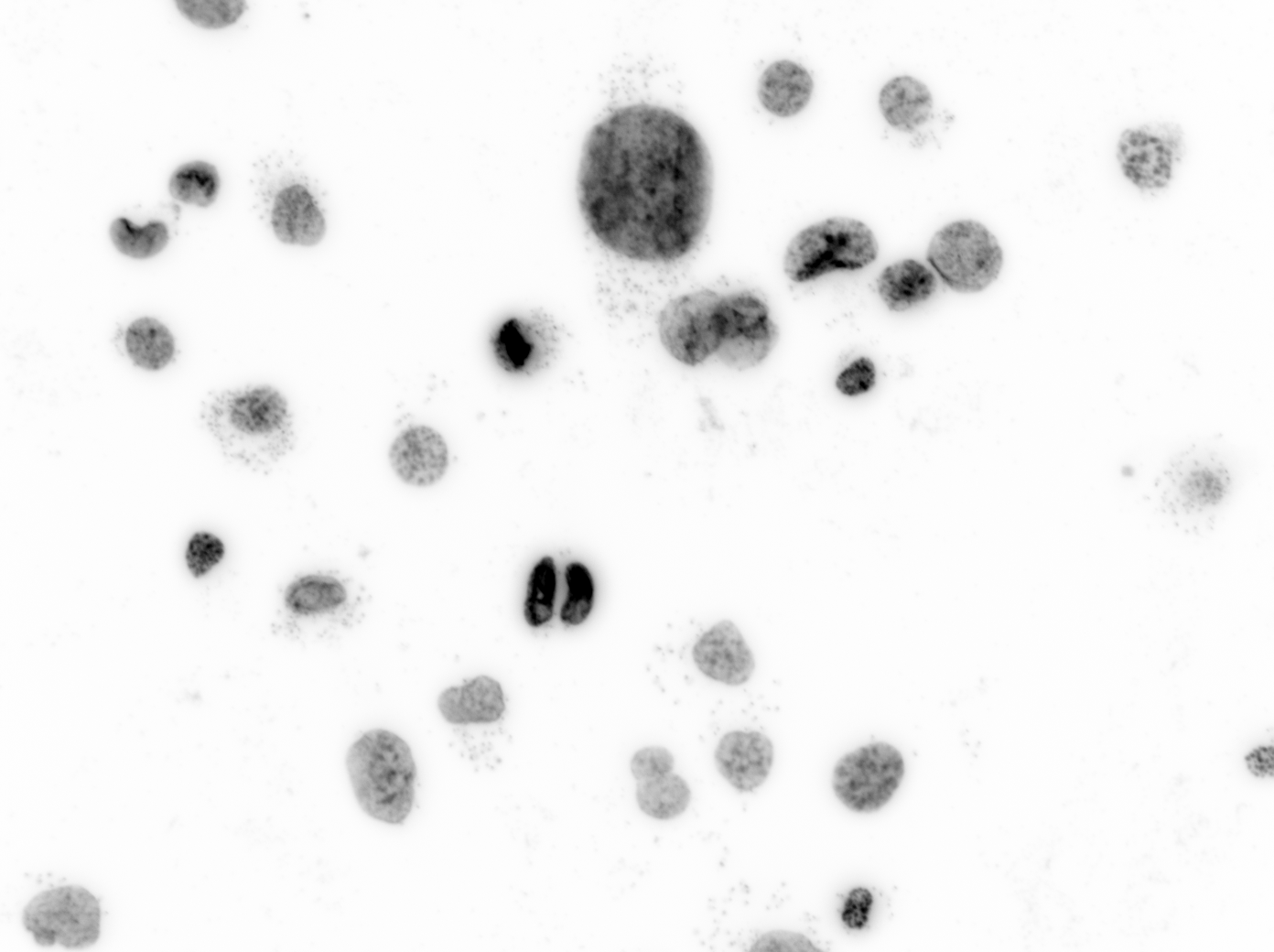

Supplement: File S2 — Transformed DAPI and Phase contrast (PC) image set of THP-1 macrophages infected by L. infantum parasites, and treated with increasing concentrations of glucantime (0-25-50-100 µg/ml). (ZIP) [file pntd.0002850.s004.zip › SI3/untreated cells/L7-DAPI.tif]

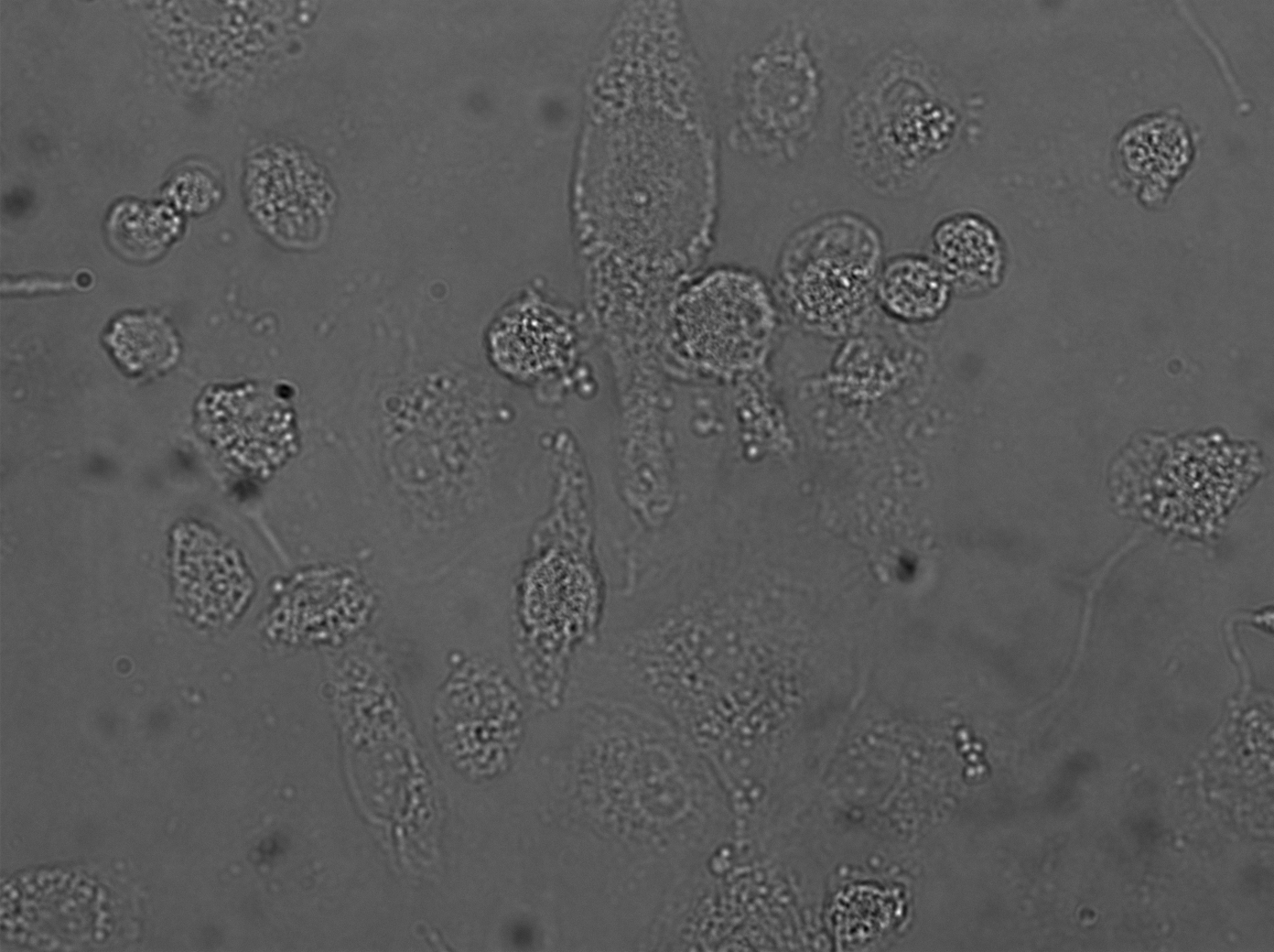

Supplement: File S2 — Transformed DAPI and Phase contrast (PC) image set of THP-1 macrophages infected by L. infantum parasites, and treated with increasing concentrations of glucantime (0-25-50-100 µg/ml). (ZIP) [file pntd.0002850.s004.zip › SI3/untreated cells/L7-PC.tif]

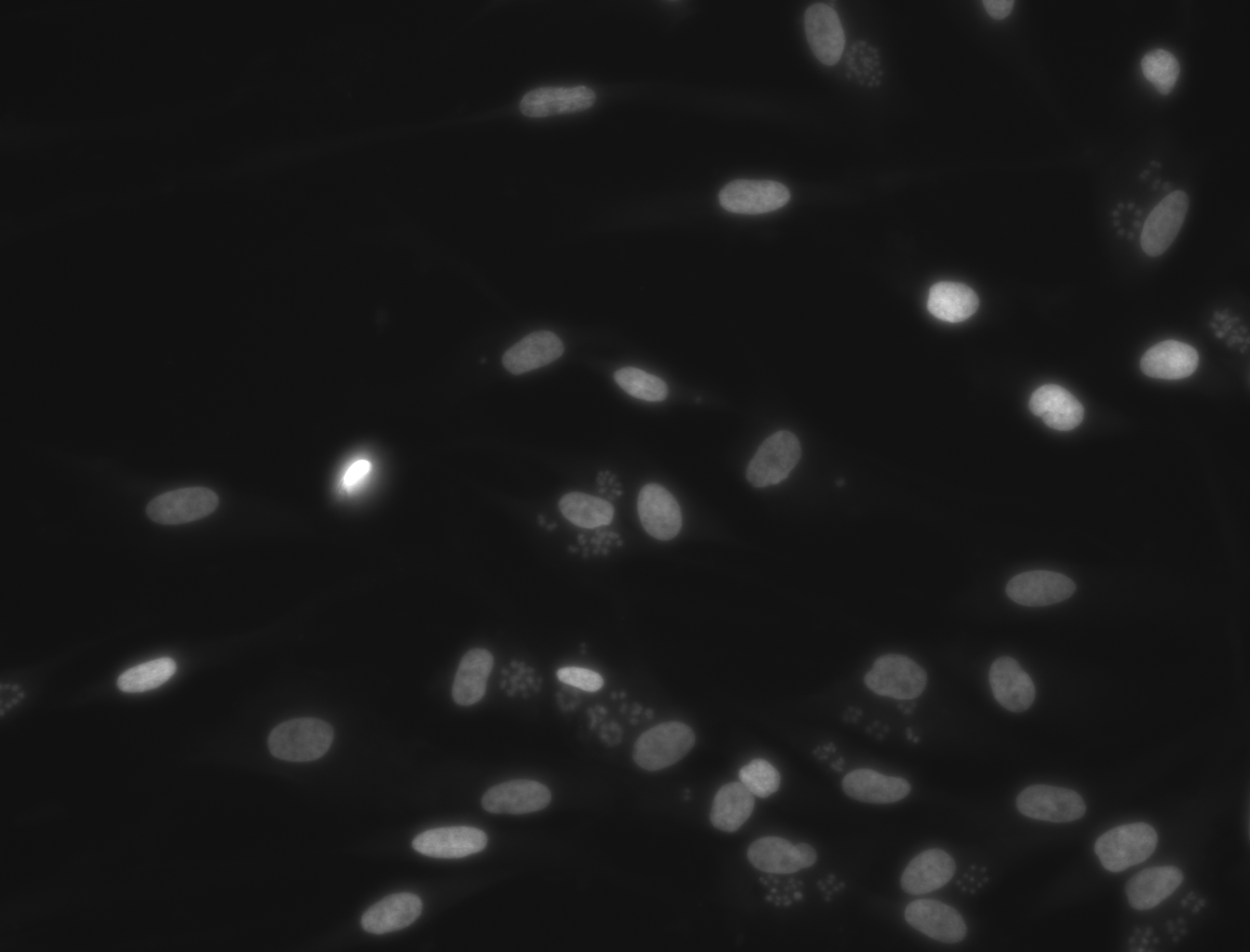

Supplement: File S3 — Representative images of HFF fibroblasts infected by T. gondii each coming as a pair with Hoechst fluorescent cell DNA stain (inverted) and the corresponding YFP-expressing RH parasites. (ZIP) [file pntd.0002850.s005.zip › Toxoplasma Hoechst/toxo1-DAPI 2.tif]

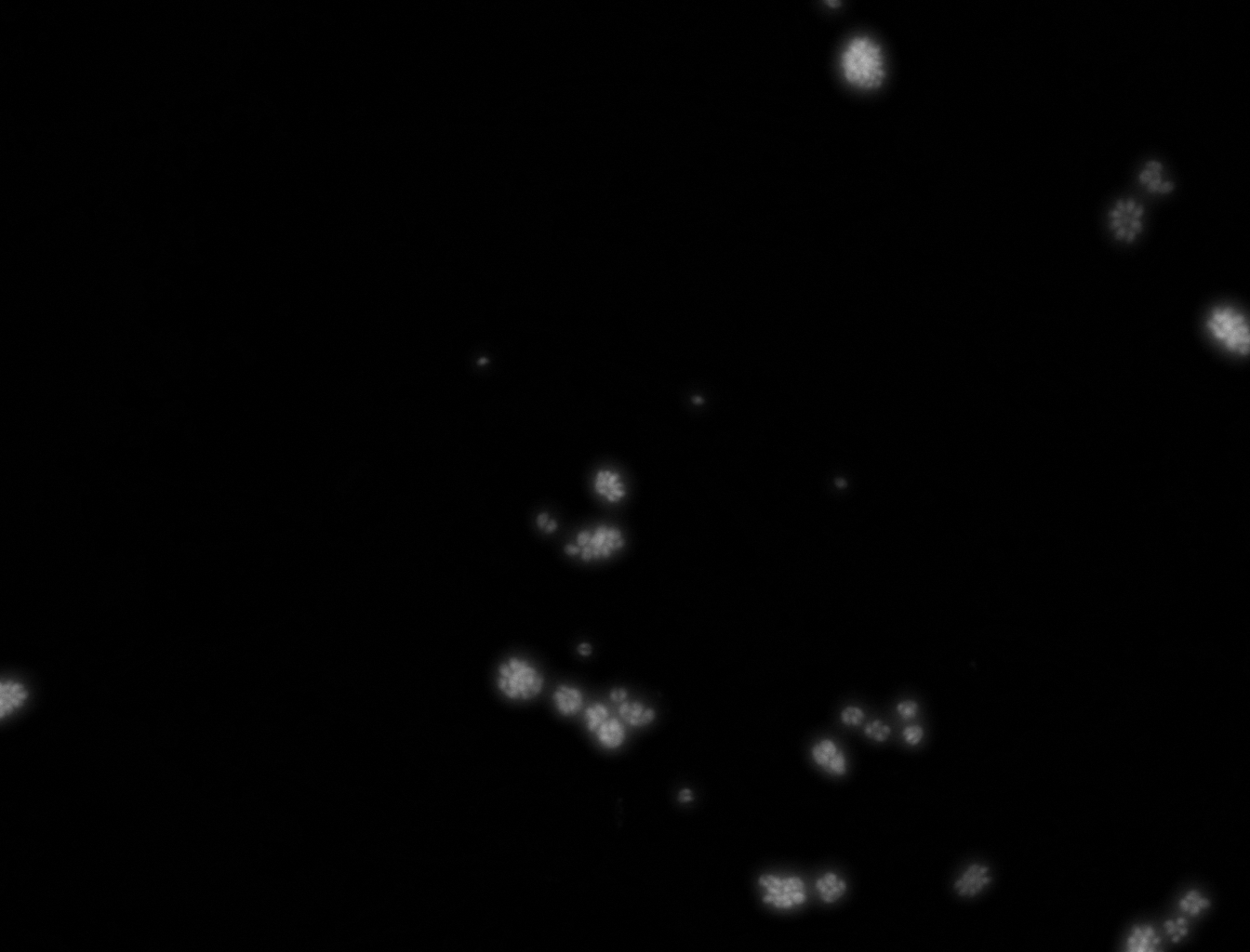

Supplement: File S3 — Representative images of HFF fibroblasts infected by T. gondii each coming as a pair with Hoechst fluorescent cell DNA stain (inverted) and the corresponding YFP-expressing RH parasites. (ZIP) [file pntd.0002850.s005.zip › Toxoplasma Hoechst/toxo1-YFP.tif]

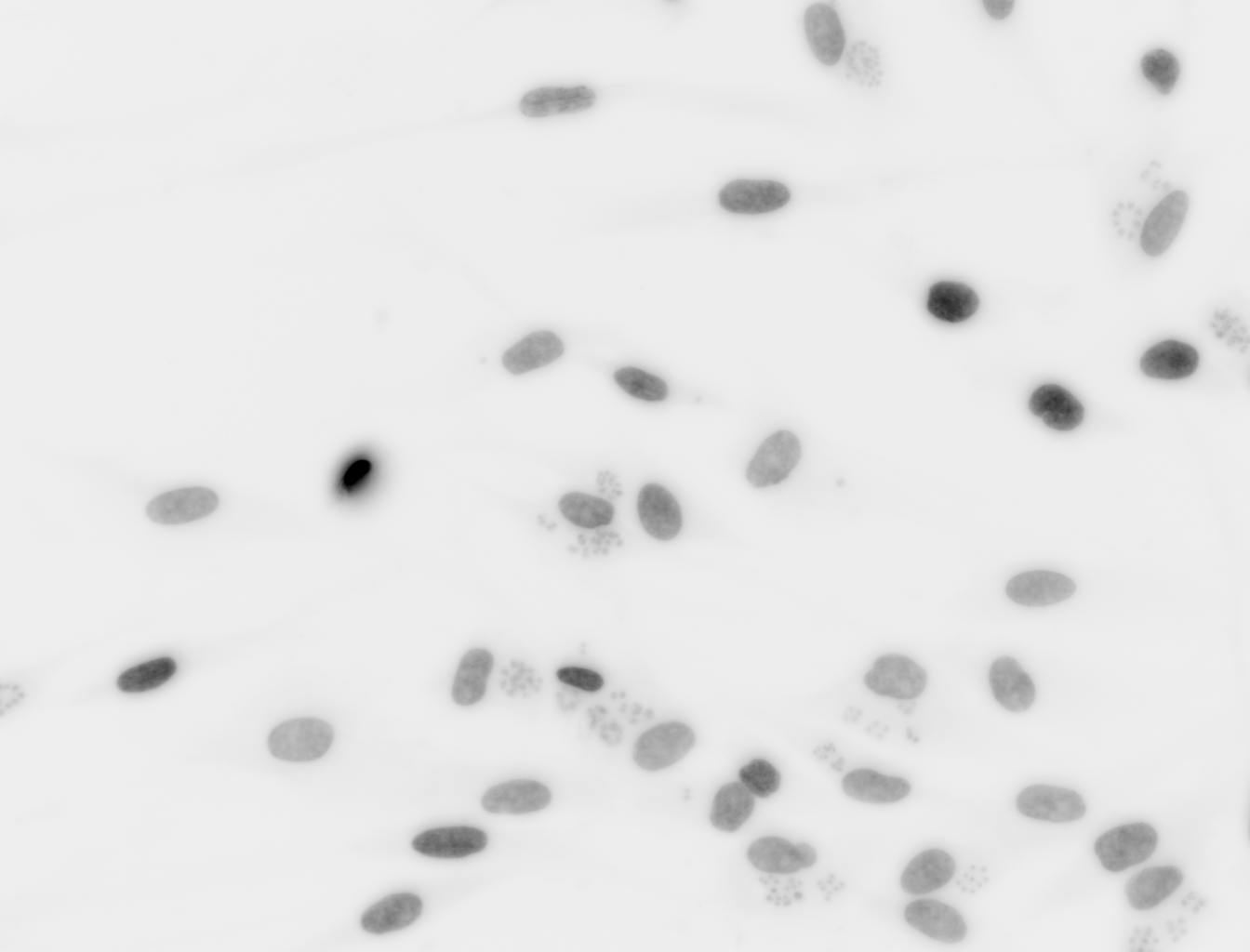

Supplement: File S3 — Representative images of HFF fibroblasts infected by T. gondii each coming as a pair with Hoechst fluorescent cell DNA stain (inverted) and the corresponding YFP-expressing RH parasites. (ZIP) [file pntd.0002850.s005.zip › Toxoplasma Hoechst/toxo1transformed-DAPI.tif]

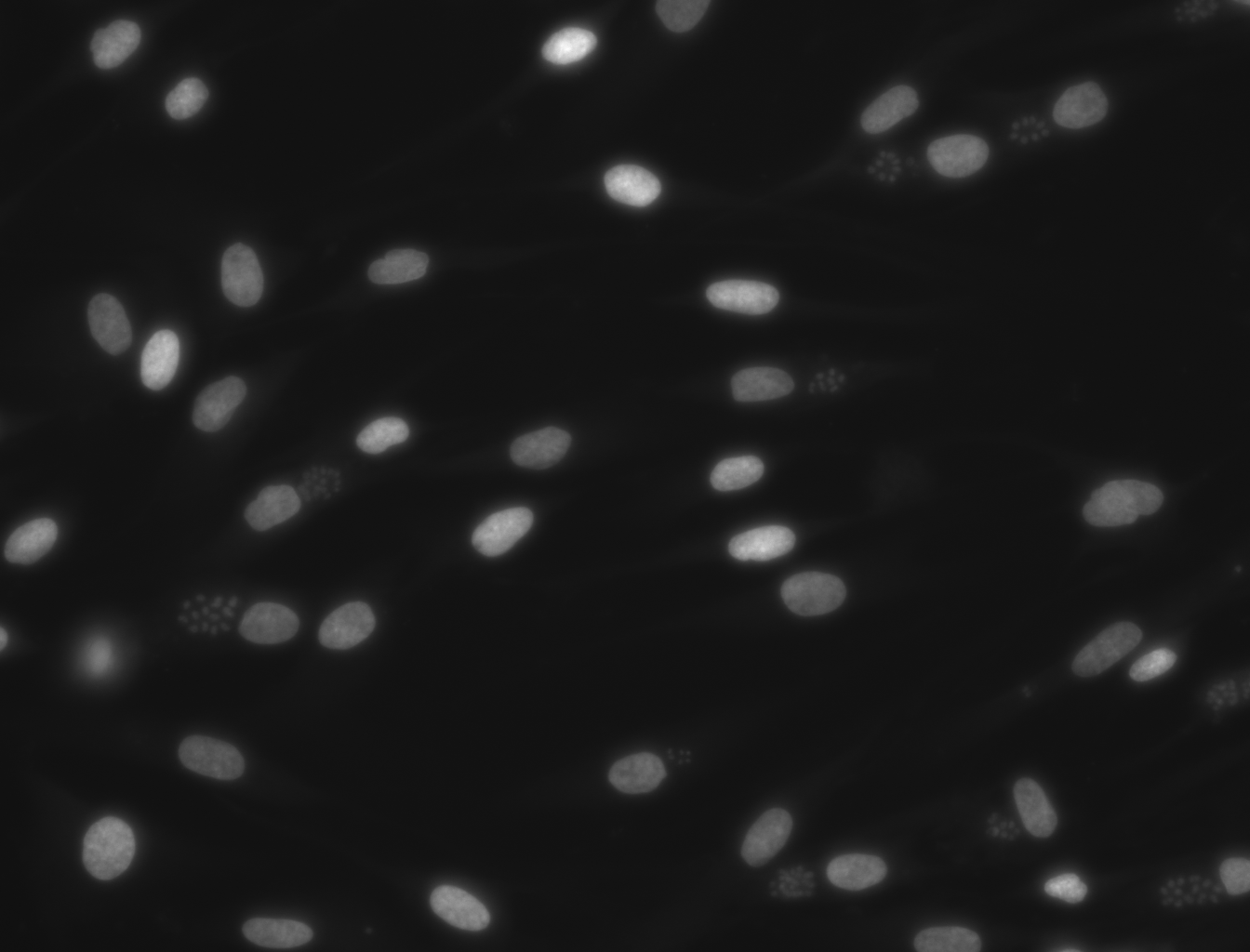

Supplement: File S3 — Representative images of HFF fibroblasts infected by T. gondii each coming as a pair with Hoechst fluorescent cell DNA stain (inverted) and the corresponding YFP-expressing RH parasites. (ZIP) [file pntd.0002850.s005.zip › Toxoplasma Hoechst/toxo2-DAPI.tif]

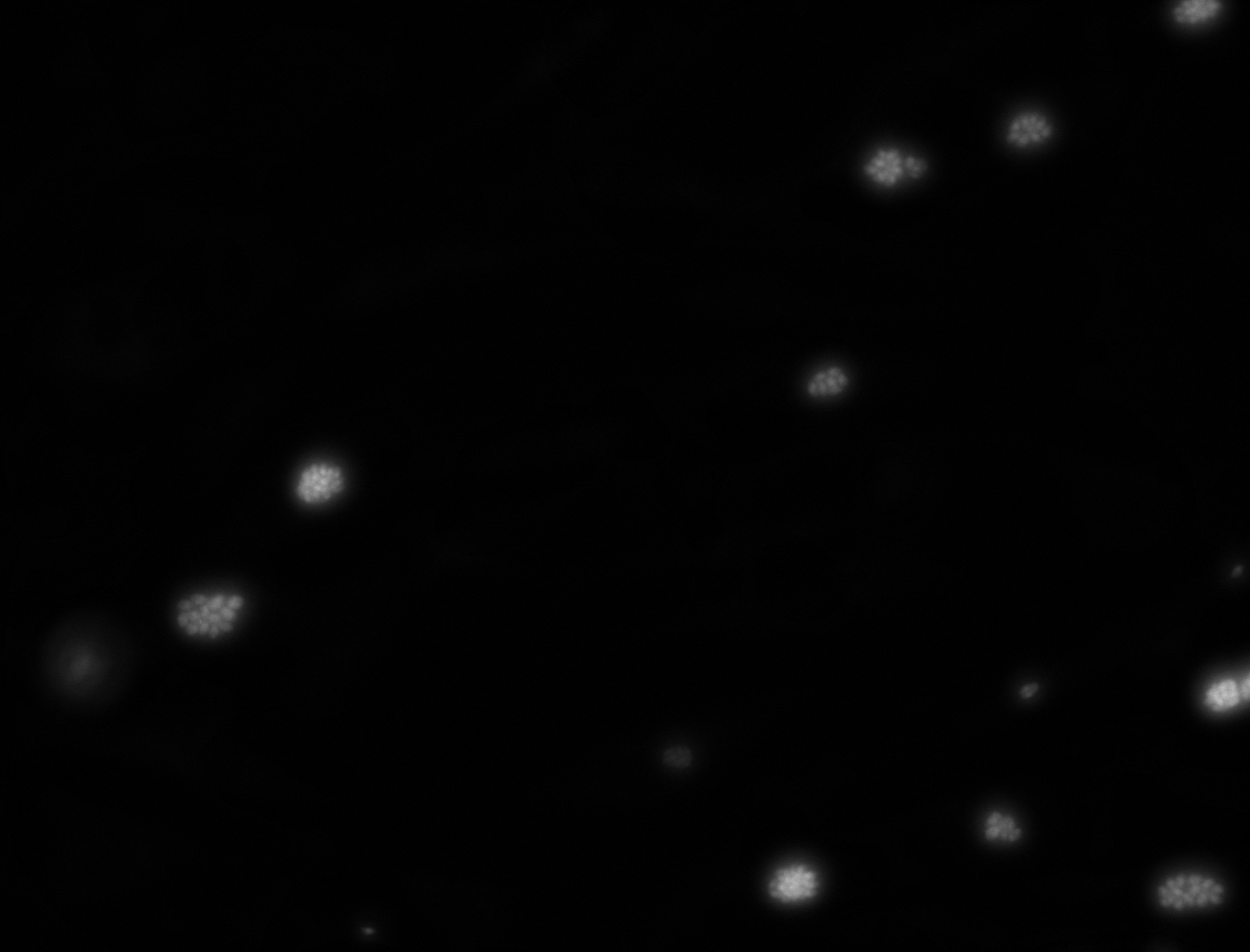

Supplement: File S3 — Representative images of HFF fibroblasts infected by T. gondii each coming as a pair with Hoechst fluorescent cell DNA stain (inverted) and the corresponding YFP-expressing RH parasites. (ZIP) [file pntd.0002850.s005.zip › Toxoplasma Hoechst/toxo2-YFP.tif]

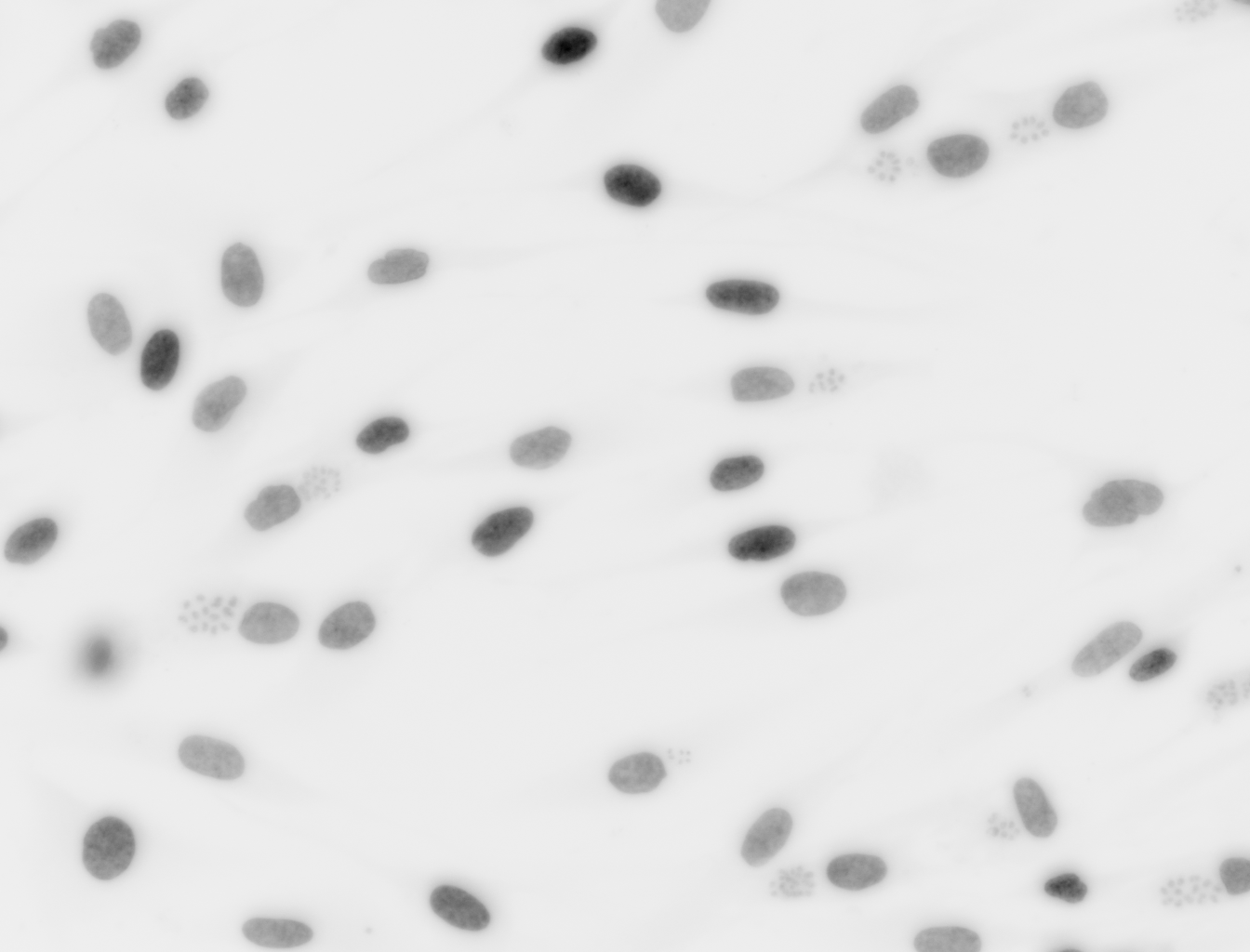

Supplement: File S3 — Representative images of HFF fibroblasts infected by T. gondii each coming as a pair with Hoechst fluorescent cell DNA stain (inverted) and the corresponding YFP-expressing RH parasites. (ZIP) [file pntd.0002850.s005.zip › Toxoplasma Hoechst/toxo2transformed-DAPI.tif]

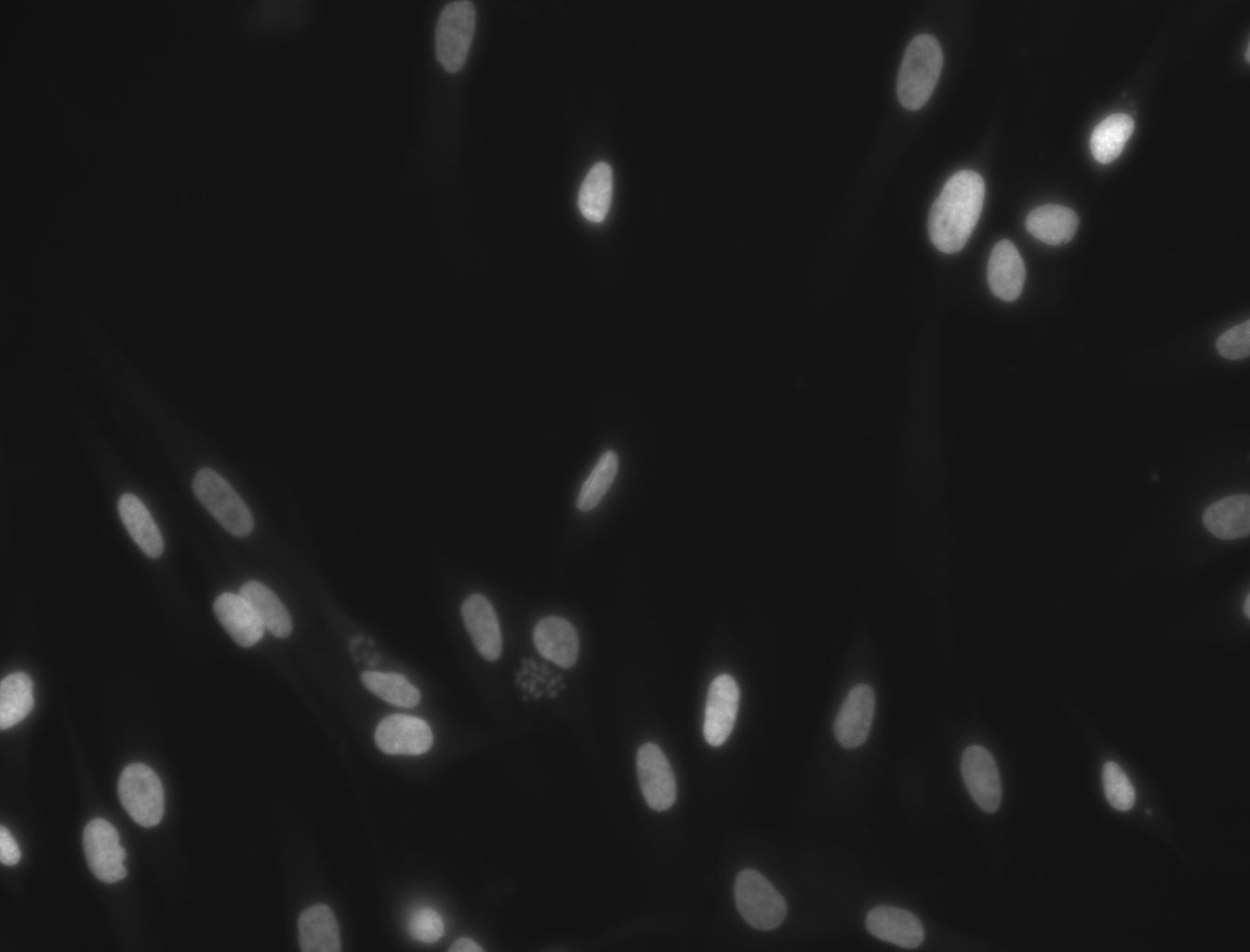

Supplement: File S3 — Representative images of HFF fibroblasts infected by T. gondii each coming as a pair with Hoechst fluorescent cell DNA stain (inverted) and the corresponding YFP-expressing RH parasites. (ZIP) [file pntd.0002850.s005.zip › Toxoplasma Hoechst/toxo3-DAPI.tif]

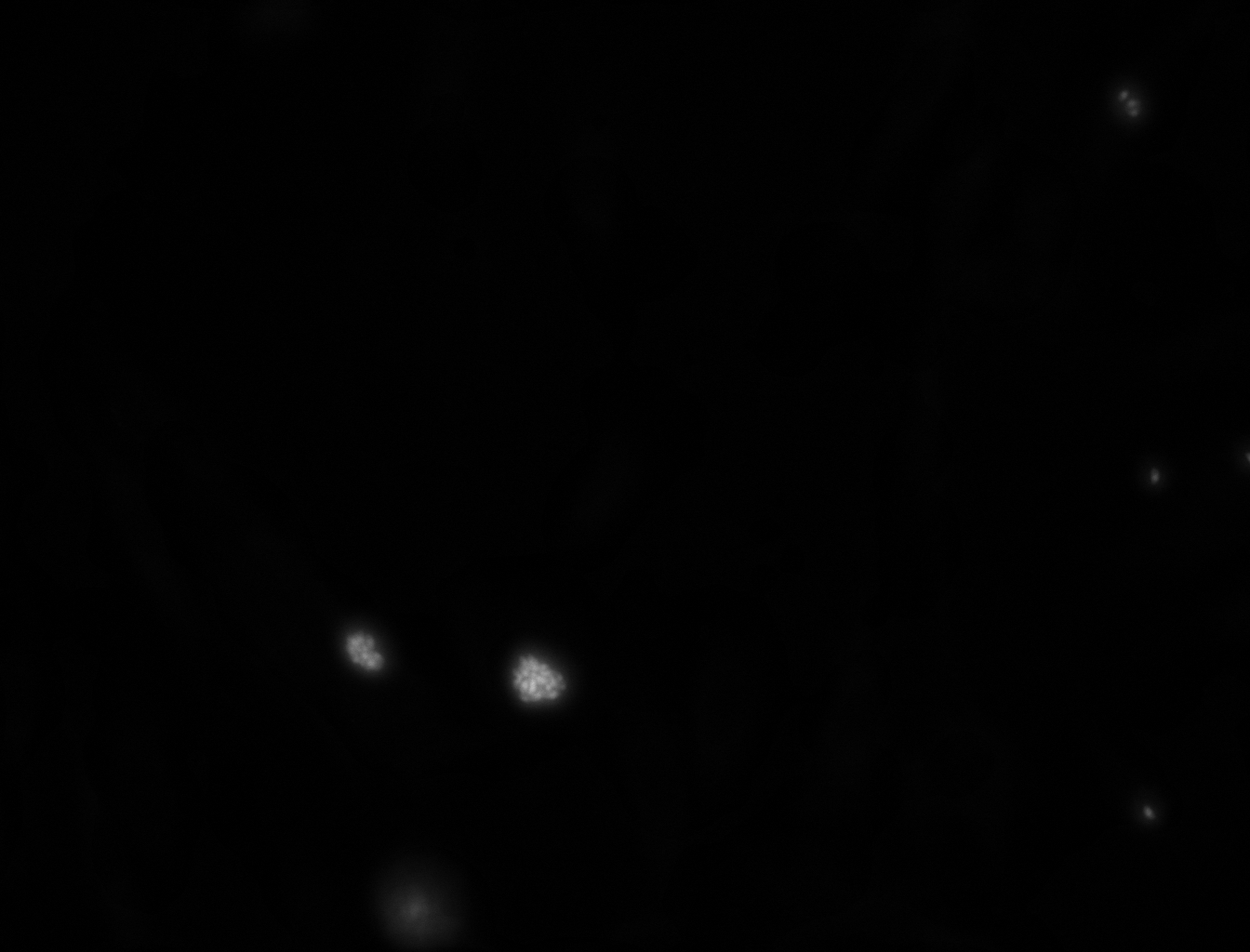

Supplement: File S3 — Representative images of HFF fibroblasts infected by T. gondii each coming as a pair with Hoechst fluorescent cell DNA stain (inverted) and the corresponding YFP-expressing RH parasites. (ZIP) [file pntd.0002850.s005.zip › Toxoplasma Hoechst/toxo3-YFP.tif]

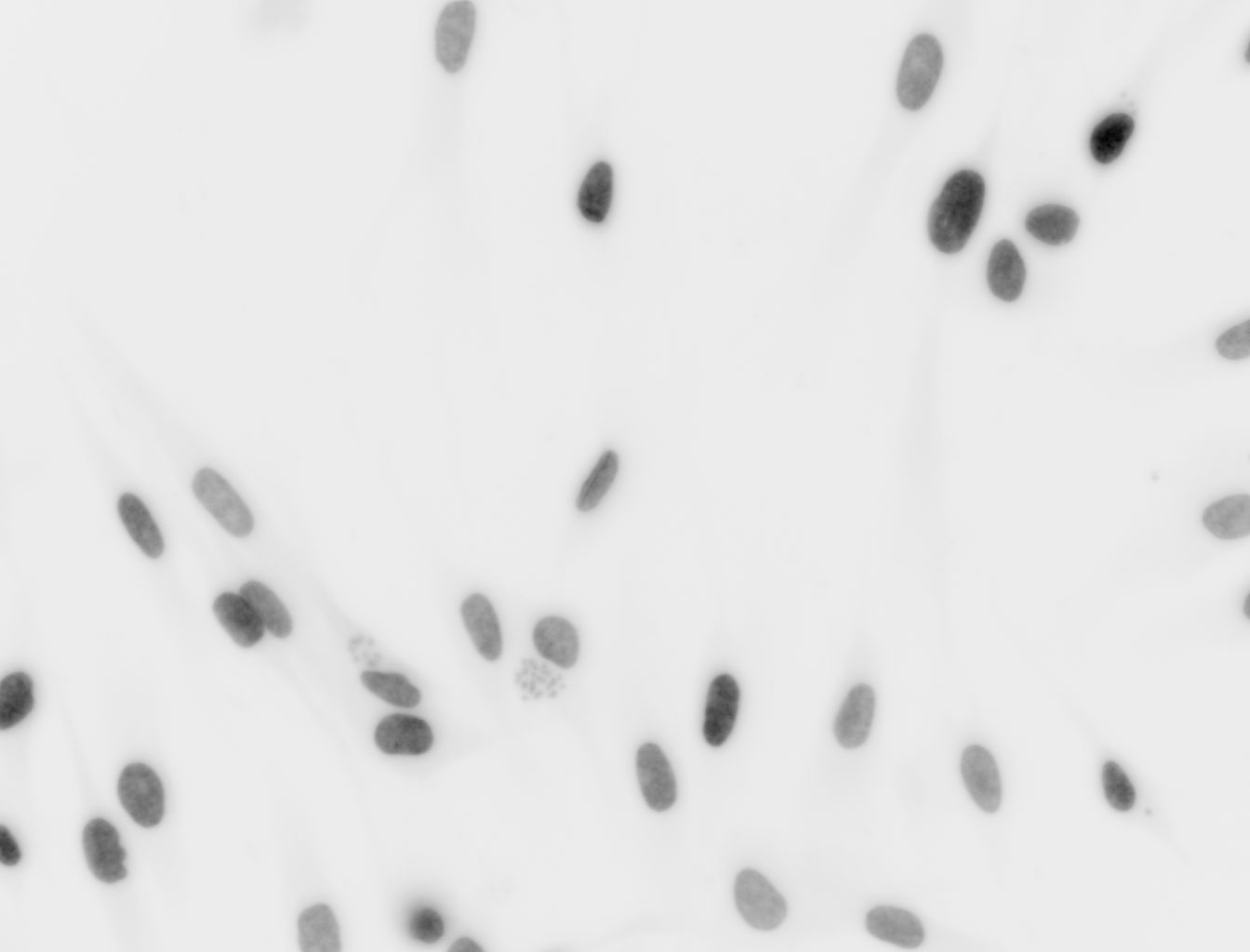

Supplement: File S3 — Representative images of HFF fibroblasts infected by T. gondii each coming as a pair with Hoechst fluorescent cell DNA stain (inverted) and the corresponding YFP-expressing RH parasites. (ZIP) [file pntd.0002850.s005.zip › Toxoplasma Hoechst/toxo3transformed-DAPI.tif]
