# Supplementary material for: INsPECT, an Open-Source and Versatile Software for Automated Quantification of (Leishmania) Intracellular Parasites
Source: PLoS Negl Trop Dis. 2014 May 15;8(5):e2850. doi: 10.1371/journal.pntd.0002850 (PMC4022486; doi:10.1371/journal.pntd.0002850)
Supplement: File S1 — INsPECT software user manual. (PDF) [file pntd.0002850.s003.pdf]

User's Guide for Image Processing Software INsPECT - Version 1.0

Author: Ehsan Yazdanparast

Contents

1 Introduction and Requirements 1
1.1 Introduction 1
1.2 Requirements 2
1.3 Input Images' Format and Protocols 2

2 Software Setup and Features 4
2.1 Software's General Flow 4
2.2 Cell Pipeline Parameters Setting 8
2.2.1 Parameters subpanel 8
2.2.2 Options subpanel 9
2.3 Parasite Pipeline Parameters Setting 10
2.3.1 Parameters subpanel 11
2.3.2 Options subpanel 11
2.4 Cytoplasm Pipeline Parameters Setting 12
2.4.1 Parameters subpanel 12
2.4.2 Options subpanel 13
2.5 Outputs 14

Chapter 1

Introduction and Requirements

This guide describes how to install and get started using INsPECT software. The following topics are covered:

- Software Introduction
- Requirements
- Input Images’ Format and Protocols
- Software Setup and Features

1.1 Introduction

INsPECT is a public domain Java-based Image Processing and analysis framework developed by Ehsan Yazdanparast. It runs as a Java application, on any computer with Java 1.6 or later installed on it. Application is available for Windows, Mac OS X and Linux.

The main functionality of this application is to process fluorescent DNA images (with or without corresponding phase contrast/DIC image sets) of *Leishmania* infected cells acquired in laboratory conditions and to generate visual and text results. Furthermore basic and common image processing operators are embedded in the software as an image viewer package.

INsPECT is being developed on Windows platform and all parts of the software are coded by the author using Java programming language.

1.2 Requirements

Before running the software, you make sure that your device has the minimum requirements for running this software.

Table 1.1: minimum requirements for running the INsPECT software

Resource	Value
Memory (RAM)	About 1GB
Hard Disk space	having high quality images and running in Full-Option mode: maximum 30MB for each input image
Processor	any x86 or x64
Operating System	Windows, Mac OS, Linux
User Permissions	Any user can run INsPECT

Notice: Due to high load of processes and to prevent software crash, it is highly recommended to run the program in devices with more RAM space.

• Try to close or idle ongoing processes while running the software to have timely results.

• In whatever platform, if you have any problems with opening the Jar file, or if the progress bar is not moving ahead, the system could not probably assign enough memory for the program heap space. In this case, you can try to run the program from the command line terminal and adjust memory allocation. When you open the terminal, you should first go to the directory in which Jar file exists, and then type this command:

Java -Xmx<maxHeapSpace>m -Xms<minHeapSpace>m -jar INsPECT.jar

For instance, the following command: **“Java -Xmx2000m -Xms128m -jar INsPECT.jar”** will start the INsPECT.jar program with minimum and maximum dedicated heap space of 128 megabytes and 2 giga bytes, respectively.

1.3 Input Images’ Format and Protocols

To feed the software with input, you need to put DNA fluorescent images (DAPI) and light microscopic images (DIC or Phase Contrast) or alternatively just DAPI images in *Input Folder*. To run the software properly, you should follow these instructions before putting images in *Input Folder*:

• All images should be in **8-bits** depth. You can simply transform input images using *ImageJ* software under the menu **image -> type -> 8-bit**

• DAPI images should be in **bright background**. If not, try **edit -> invert** on ImageJ.

• Transformed DAPI and corresponding light microscopic images pairs should be placed **exactly** after each other. Furthermore, for each pair, DAPI image should be placed **before** its corresponding DIC or Phase Contrast image (see Fig. 1.1).

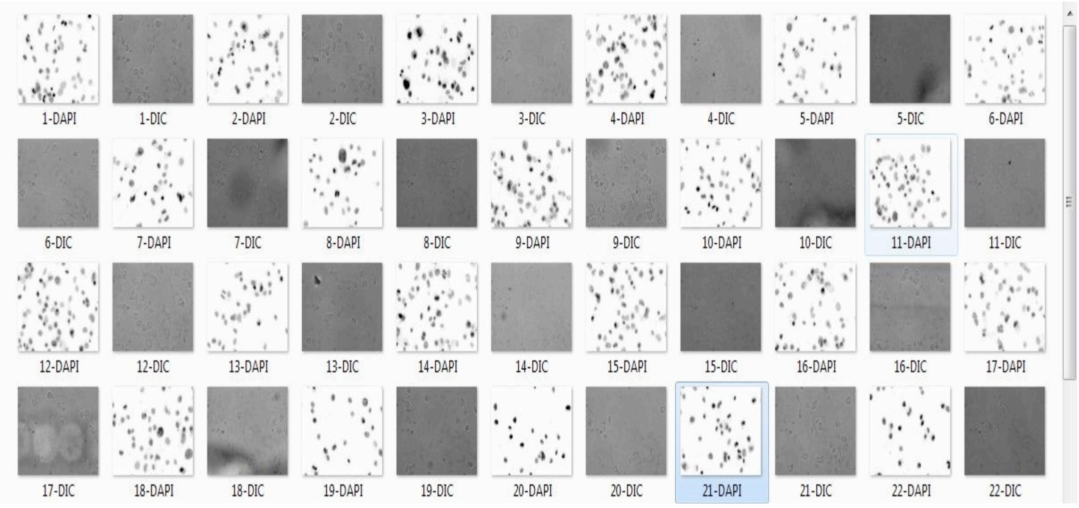

Figure 1.1: Right order of putting images in Input Folder

Chapter 2

Software Setup and Features

When you run the software, the main panel will be opened where you can access to options with both menu items and toolbar icons (see Fig. 2.1).

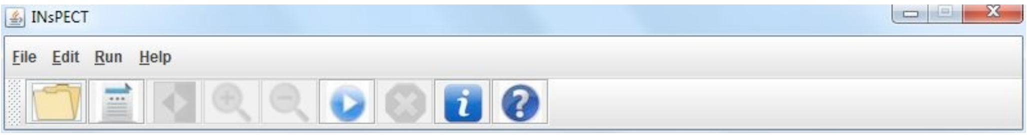

Figure 2.1: Main Panel of INsPECT

First of all, an *Image Viewer* with basic Image manipulation operators are embedded in the software. These functionalities could be accessed through the main frame of the software. Options are listed below:

- Open and Close images
- Zoom In/Zoom Out current image
- Invert current Image

2.1 Software's General Flow

To process input images and generate results and reports, two scenarios are embedded in the software (see Fig. 2.2):

1. **Automatic** running method in which pipeline parameters are assigned to input images automatically. In fact, according to experimental and analytical results, default values for each parameter are set in this method.

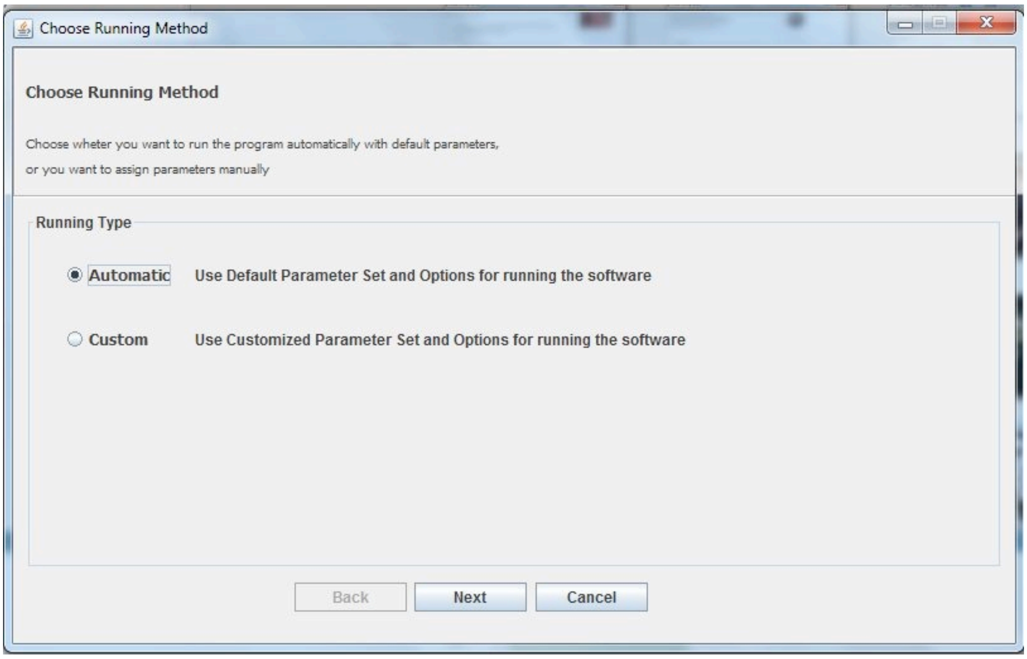

Figure 2.2: Running method selection panel: users can choose Custom and Automatic methods to run the software

2. **Custom** running method that allows the user to specify his/her intended parameters, step by step. If the user chooses this option, cell, parasite and cytoplasm pipeline parameters are taken in three steps from the user and then program will go to *Ready to Run* state.

Users are recommended to first use the software in *Automatic* mode, to test it and if the results are not satisfactory enough for them, they can then switch to *Custom* mode and feed the software with their parameters. *Automatic* mode's results could also be a very promising way for users to gain ideas regarding parameter ranges.

Using both scenarios, users also should specify some Running Parameters through a panel (see Fig. 2.3) as follows:

1. **Directories:** Select *Input Folder* that contains input image set and *Output Folder* to save the results and reports. Notice that all results, containing images and reports will be saved under *Output Folder.*
2. **Input Images' Type:** the software comes with two options for processing input image set. If you choose *DAPI and Phase Contrast* option, then you should put pairs of such images in *Input Folder*. Alternatively, with choosing *Just DAPI* option, software will process input DAPI images only.

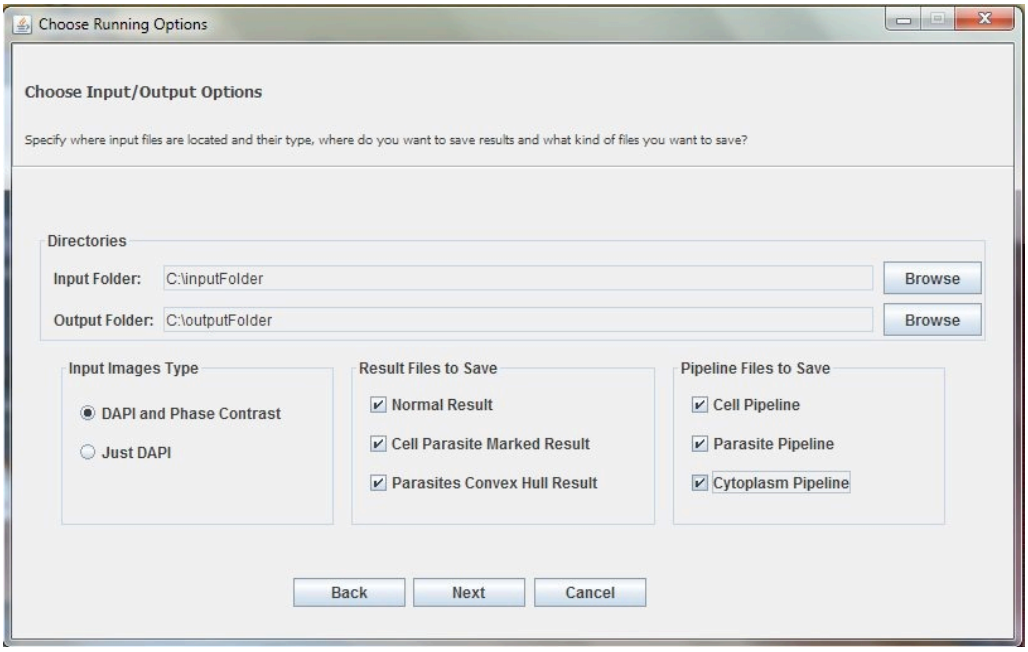

Figure 2.3: Running Options Panel: Users should specify running options for both automatic and custom scenarios

3. **Results Files to Save:** *Normal Result* is the processed image with markers and regions boundaries. *Cell Parasite Marked Result* is similar to *Normal Result*, except that cells and parasites are labeled using numbers in such images. Finally, *Parasites' Convex Hull Result* images show convex hull of related parasites for each cell. If checked, any of those results will be saved under *Output Folder* and in relevant sub directory for related input image(s).

4. **Pipeline Files to Save:** each checkbox, namely *Cell Pipeline*, *Parasite Pipeline* and *Cytoplasm Pipeline*, if checked, then related output images for that pipeline will be saved under *Output Folder* and in relevant sub directory for related input image(s). Notice that, if input images' type is chosen to be *Just DAPI*, *Cytoplasm Pipeline* checkbox will be disabled.

If you use the software in *Automatic* mode, after choosing above running options, the program is ready to run for you. And you can run the software in the next panel by clicking *Run* button (see Fig. 2.4 and Fig. 2.5).

If you choose *Custom* mode, then you need to specify parameters manually through three upcoming panels, called *Cell Pipeline Running Parameters*, *Parasite Pipeline Running Parameters* and *Cytoplasm Pipeline Running Parameters*.

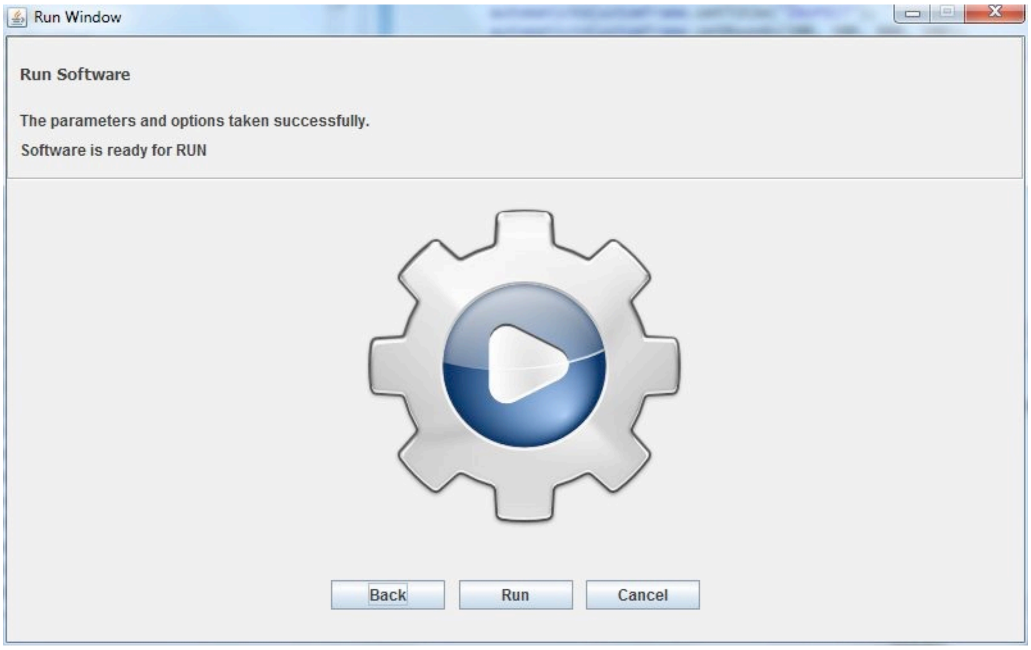

Figure 2.4: Ready to Run Panel: After taking parameters and options (manually or automatically), by clicking on Run button, software starts to process the input images

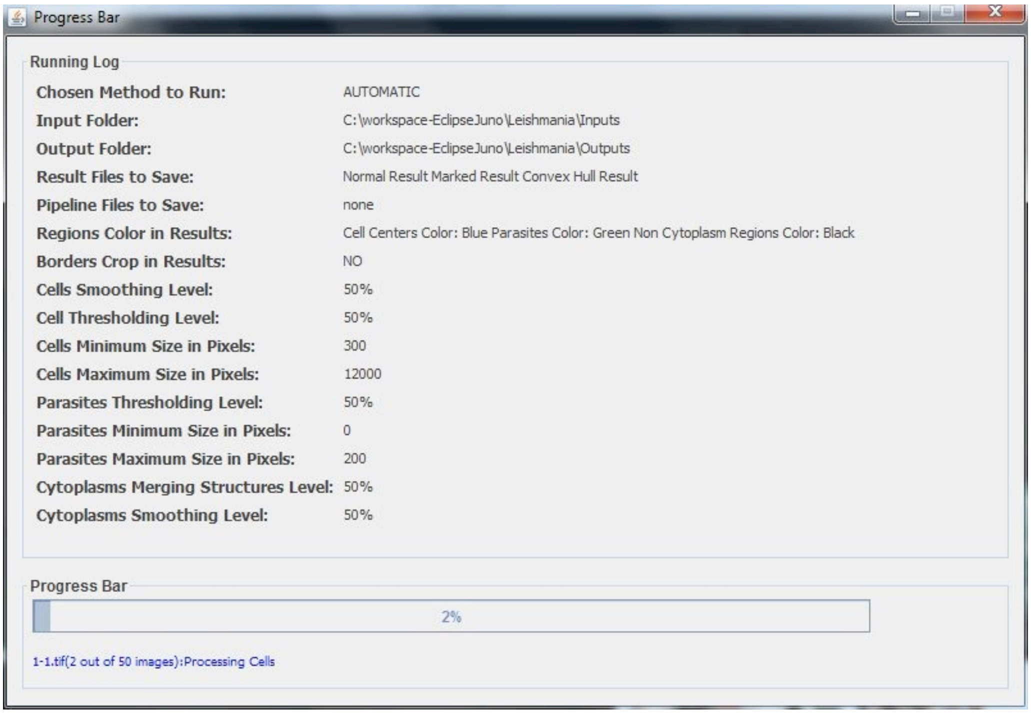

Figure 2.5: Runtime Log Panel: During Process time, users can trace flow of running software in this panel

Before explaining in details parameter settings of these three panels, it should be mentioned that *INsPECT* software is designed to biologists without image processing background. Therefore, for all needed parameters, more meaningful and touchable synonyms are defined, and numeric values are substituted with labels such as *Low*, *Medium* and *High*.

2.2 Cell Pipeline Parameters Setting

In *Cell Pipeline Running Parameters* panel, you can adjust parameters to detect cell nuclei in DAPI input images (see Fig. 2.6). It includes two subpanels, called *Parameters* and *Options*.

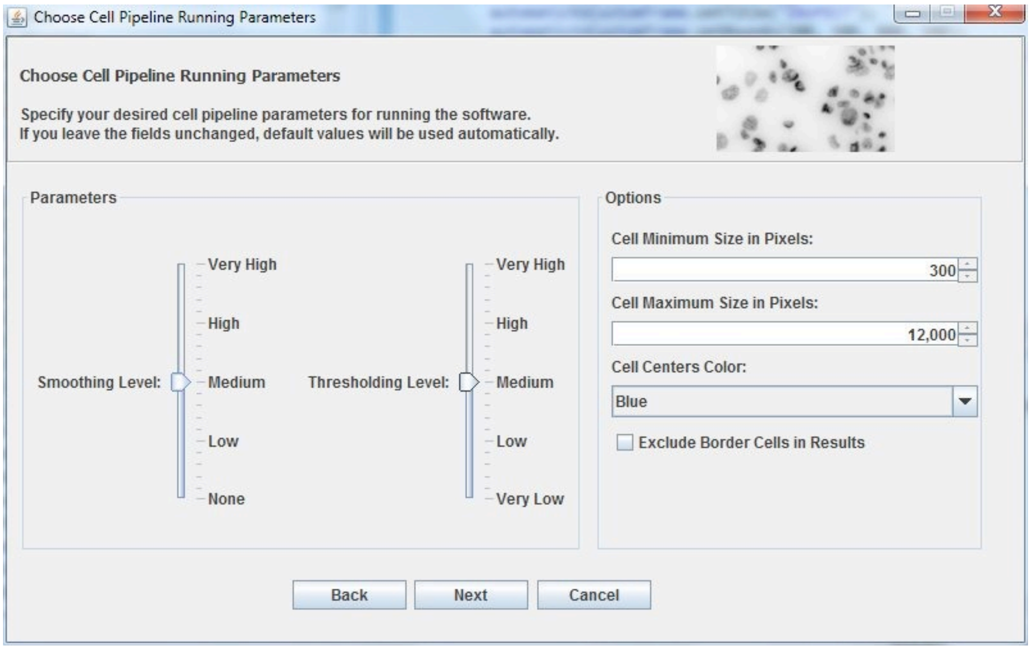

Figure 2.6: Cell Pipeline Running Parameters Panel

2.2.1 Parameters subpanel

- **Smoothing Level:** in terms of *Image Processing* operators it is equivalent to *Median Kernel Radius*. It is the main parameter to tell the algorithm how much it should smooth and denoise DAPI input images. The default value is set to numeric value 15 (which is labeled as *Medium*) and is logical when the quality of input DAPI image is reasonable and doesn't contain that much noise. If cells have very clear boundaries and noise does not exist across the image, *Smoothing Level* can be decreased. Alternatively, it can be increased for low quality images. None label is

equivalent to 0 and Very High to 35 numeric values respectively.

Notice: if you have low quality DAPI images and use high *Smoothing Levels*, algorithm will overestimate the volume of the cells. For such images, low *Smoothing Level* will also lead to misdetection of cell portions because of the existence of noise and other by-products.

• **Thresholding Level:** in terms of *Image Processing* operators it is equivalent to *Mean Adaptive Threshold Kernel Radius*. This parameter is used locally in subimages to make the binarization of cell portions and background area. The default value (which is labeled as *Medium*) is calculated automatically based on global standard deviation of current image in process and is set to 1.5 times of this value. This is logical when the density of cell is not too high and distribution of cells is not very unbiased across the image. For DAPI images with high cell density (majority of dark points), you can increase *Thresholding Level* to higher degrees. On the other hand, for DAPI images with lower cell density, you should decrease *Thresholding Level* to overcome unbiasness of foreground and background pixels. *Very low* label is equivalent to 1 numeric value and *Very High* label is equivalent to 140.

2.2.2 Options subpanel

• **Cell Minimum (Maximum) Size in Pixels:** these two parameters help algorithm to filter out those detected cell portions in final result which do not have sizes between *Minimum* and *Maximum*. The default values for minimum and maximum size are set to 300 and 12000 pixels, respectively.

Notice: Sometimes high density of parasites exist around some cells and denoising them would lead to miss some valuable border data of cells in final results. However, such kind of misdetected portions can be ignored by setting *Cell Minimum Size in Pixels* properly. Experiments with available image sets in hand showed that size 300 is a suitable choice to filter out those regions while preserving all other valid spots.

• **Cell Centers Color:** you can choose in which color you want to see the cells' centers' markers in final output.

• **Exclude Border Cells in Results:** Checking this option will allow the algorithm to ignore incomplete cell's nuclei in edges and borders in final visualized results and calculations.

2.3 Parasite Pipeline Parameters Setting

In Parasite *Pipeline Running Parameters panel*, you can adjust parameters for extracting parasites in DAPI input images (see Fig. 2.7). It includes two subpanels, called *Parameters* and *Options*.

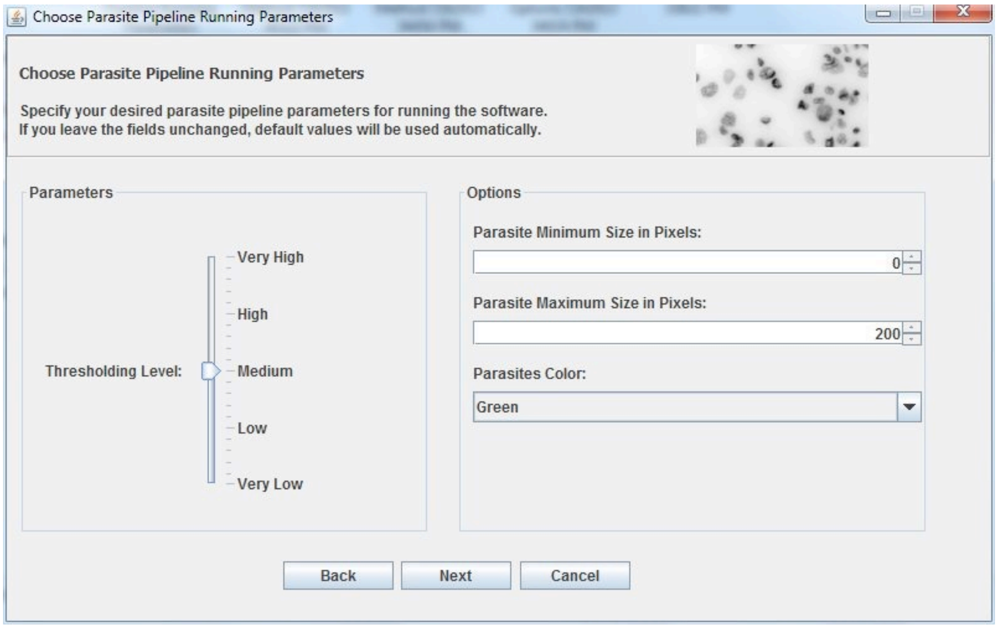

Figure 2.7: Parasite Pipeline Running Parameters Panel

2.3.1 Parameters subpanel

- **Thresholding Level:** in *Image Processing* terms, this parameter is binded to *Structuring Element Size of Black Top Hat Transform operator*. The size of the *Structuring Element* is highly dependent on nature of image structures we want to analyze, keep or suppress. Here, the components of the interest for suppressing are parasites nuclei. Therefore, increasing or decreasing the value of *Thresholding Level* will enable algorithm to suppress those components more or less. The default value is set to 2 (labeled as *Medium*) and works quite fine for most of DAPI stained images we had to analyze. However, for any reason, if parasites nuclei are bigger (or smaller) than normal, then you could increase (or decrease) *Thresholding Level*. In this case, *Structuring Element* will be bigger (or smaller) and as a result, it will suppress bigger (or smaller) candidate areas for parasites. *Very Low* label is equivalent to 1 numeric value and *Very High* label is equivalent with numeric value 4.

Notice: Decreasing *Thresholding Level* is not recommended since using this option, often

leads to overdetection of parasites. However, if users are aware of the fact that in reality, such density of parasites exists around cell portions, they can use this option too.

2.3.2 Options subpanel

- **Parasite Minimum (Maximum) Size in Pixels:** these two parameters help the algorithm to filter out those detected parasites which do not meet requirements of size range. The default size values for *Minimum* and *Maximum* is set to 0 and 100 pixels, respectively.

Notice: in some images, there may exist detected parasites with very small sizes, namely 1 to 10 pixels. It is recommended for users not alter minimum size for parasites unless they are confident that such tiny details are not parasites and could be any other by-products.

- **Parasites Color:** you can choose in which color you want to see the parasites markers in the final output.

2.4 Cytoplasm Pipeline Parameters Setting

In *Cytoplasm Pipeline Running Parameters* panel, you can adjust parameters to extract cytoplasm traces from Phase Contrast or DIC input images (see Fig. 2.8). It includes two subpanels, called *Parameters* and *Options*.

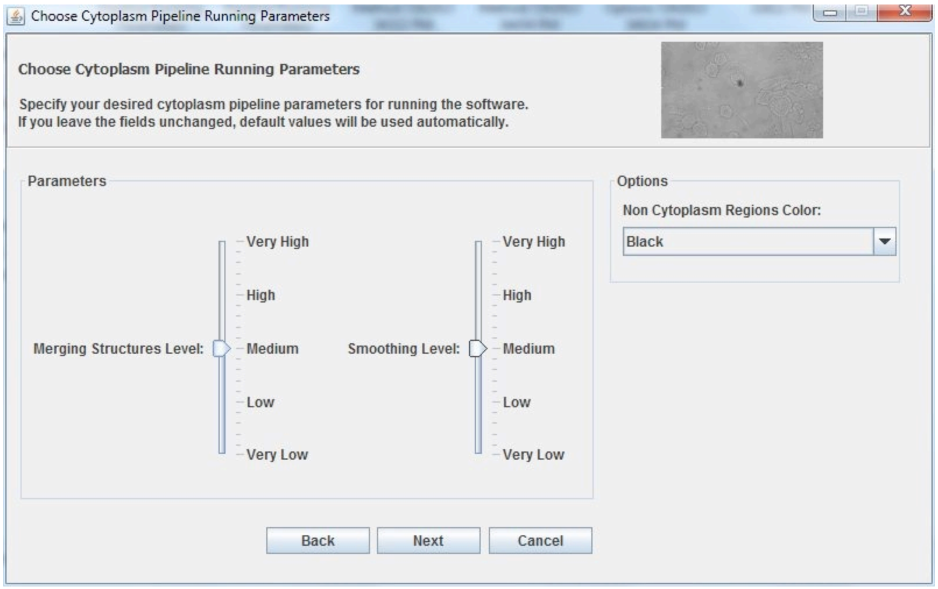

Figure 2.8: Cytoplasm Pipeline Running Parameters Panel

Notice: If you have already chosen the option *Just DAPI* to run the software, then this panel will not be shown to you since processing Phase Contrast or DIC input images are no longer the concern.

2.4.1 Parameters subpanel

- **Merging Structures Level:** in terms of *Image Processing* operators, this parameter is equivalent to *Structuring Element Size of Morphological Closing* operator. This operator is used to merge and fill the structures of detected cytoplasmic areas after smoothing and thresholding. The default value is set to 7 (which is labeled as *Medium*) and is acceptable when Phase Contrast or DIC input image is not suffering from very high illumination variance (so the thresholded cytoplasm traces need to be merged more). If cytoplasmic areas cannot be well detected in a straightforward manner, then increasing *Merging Structures Level* value could help to at least find merged portions for cytoplasms. On the other hand, if quality of DIC or Phase Contrast images are good and traces are visible and easily detectable, then decreasing *Merging Structures Level* value helps the algorithm to find more exact traces around cells. *Very Low* label is equivalent to 1 numeric value and *Very High* label is equivalent with a numeric value of 14.

Notice: DIC or Phase Contrast input images suffer from high illumination variance. To compensate such effect, we use *Merging Structures Level* parameter to have at least more smooth and connected regions. Accordingly, altering this value could sacrifice exact detection of some cytoplasmic regions.

- **Smoothing Level:** in terms of *Image Processing* operators, this parameter is equivalent to *Median Filter Kernel Radius* and it is used to smooth the *Morphological Closing* result, fill small holes and discard small detected portions. The default value is set to 50 (which is labeled as *Medium*). This parameter also tries to compensate the effects of bad quality of cytoplasm traces and its task is more or less similar to *Merging Structures Level* parameter. *Very Low* label is equivalent to 1 numeric value and *Very High* label is equivalent with a numeric value 50.

2.4.2 Options subpanel

- **Non Cytoplasm Regions Color:** you can choose in which color you want to see the non cytoplasmic regions in the final output. The regions encapsulated inside

cytoplasm regions will be shown in original image in results.

2.5 Outputs

Upon completion of the running of the software, visual and text results will be saved under *Output Folder* directory. Visual outputs are already explained in section 2.1. In each run, software also generates two report files, called *report* and *cell parasites report*.

The *report* file contains general outcomes of processing each pair (or individual). *FileName*, *Total Number of Cells*, *Number of Infected Cells*, *Total Number of Parasites*, *Total Number of Intracellular Parasites*, *Percentage of Infected Cells*, The *Mean Number of Parasites per Cell* and *Parasitic Index* (Percentage of *Infected Cells* x *Mean Number of Parasites Per Cell*) are the parameters recorded in this file for each image pair (or individual).

The *Cell Parasites Report* keeps the record of each cells' features in image pairs (or individual). You can find these fields in this report: *FileName*, *Cell Number*, *Cell Area*, *Cell Volume*, *Total Number of Parasites*, *Number of Intracellular Parasites* and *Number Of Extracellular Parasites*.

Notice: *Cell Parasites Report* could be helpful when one wants to investigate individual cells. On the other hand, *report* file is useful for generic analysis.
